# Extended Reality Interventions for Osteoarthritis of the Knee and Recovery After Total Knee Arthroplasty: Systematic Review and Meta-Analyses

**DOI:** 10.2196/84753

**Published:** 2026-09-08

**Authors:** Caleb Kalinowski, Elizabeth Goldsmith, Maylen Anthony, Adrienne Landsteiner, Kristen Ullman, Nicholas Zerzan, Tonya Rich, Collin Calvert, Timothy J Wilt, Wei Duan-Porter, David Ewart

**Affiliations:** 1 Veterans Affairs Evidence Synthesis Program, Center for Care Delivery Outcomes Research, Minneapolis Veterans Affairs Health Care System, One Veterans Drive, Minneapolis, MN, 55417, United States, 1 612-467-1919; 2 Department of Medicine, University of Minnesota Medical School, Minnneapolis, MN, United States; 3 Department of Orthopedic Surgery, University of Minnesota Medical School, Minnneapolis, MN, United States; 4 Rehabilitation and Engineering Center for Optimizing Veteran Engagement and Reintegration, Minneapolis Veterans Affairs Health Care SystemMinneapolis, MN, United States; 5 Department of Family Medicine and Community Health, University of Minnesota Medical School, Minnneapolis, MN, United States; 6 Center for Care Delivery Outcomes Research, Minneapolis Veterans Affairs Health Care SystemMinneapolis, MN, United States; 7 University of Minnesota School of Public Health, Minnneapolis, MN, United States; 8 Minneapolis Veterans Affairs Health Care SystemMinneapolis, MN, United States

**Keywords:** knee pain, knee osteoarthritis, total joint arthroplasty, total knee arthroplasty, extended reality, virtual reality, rehabilitation

## Abstract

**Background:**

Nonpharmacologic interventions are important for treating knee pain due to osteoarthritis or after total knee arthroplasty (TKA), and extended reality (XR) technology may enhance treatments for these indications.

**Objective:**

This systematic review aimed to evaluate XR interventions for pain due to knee osteoarthritis (KOA) or for recovery after TKA.

**Methods:**

Databases were searched through May 2023 and updated in December 2025. Eligible trials evaluated XR interventions to treat KOA pain or after TKA. We classified interventions by depth of immersion and clinical mechanism. We used the Grading of Recommendations Assessment, Development, and Evaluation (GRADE) criteria to determine the certainty of evidence for prioritized outcomes. Meta-analyses were performed when ≥3 studies evaluated similar comparisons, outcomes, and time points.

**Results:**

Eligible trials addressed KOA (k=12) or recovery after TKA (k=9). Sample sizes ranged from 36 to 306 participants, and most studies had a follow-up of ≤3 months. Nineteen studies assessed pain-related functioning and pain intensity, and 5 assessed adverse events (AEs). For KOA, 10 studies examined interactive digital rehabilitation (IDR), and 2 examined virtual reality (VR)–digitally augmented exercise (DAE). IDR for KOA may result in better pain-related functioning (low certainty of evidence [COE]; pooled standardized mean difference [SMD] −0.59, 95% CI −1.11 to −0.06; prediction interval [PI] −1.72 to 0.55; k=5) and lower pain intensity at 6‐8 weeks (low COE; pooled SMD −0.46, 95% CI −0.92 to 0.00; PI −1.39 to 0.47; k=4). VR-DAE for KOA (k=2) produced inconsistent results (very low COE). For post-TKA studies, 5 examined IDR, 2 examined VR-DAE, 1 examined VR-distraction, and 1 examined VR-psychoeducation. Post-TKA IDR may result in better pain-related functioning (low [k=4] and moderate COE [k=1]) but little to no difference in pain intensity (low-moderate COE; pooled SMD at 3‐4 months −0.12, 95% CI −0.75 to 0.52; PI –1.63 to 1.27; k=3). VR-psychoeducation probably results in lower pain at 4 weeks (moderate COE; k=1), and VR-distraction may result in 6 months (low COE; k=1), whereas VR-DAE produced mixed findings (k=2; very low COE). IDR was not associated with AEs, and VR may not be associated with AEs for KOA (high and low COE), though AE reporting was uncommon (k=5) and evidence was very uncertain for post-TKA.

**Conclusions:**

IDR may augment treatment for KOA and post-TKA recovery, and VR may benefit post-TKA rehabilitation. This review is the first to stratify by level of immersion, clinical mechanism, and follow-up duration and to systematically evaluate AEs. IDR may be ready for integration into KOA care, while use after TKA needs more evidence. Randomized controlled trials with implementation outcomes could determine how XR interventions can be used for KOA, whereas trials evaluating efficacy and AEs are needed before their use for post-TKA.

## Introduction

### Background

Osteoarthritis of the knee is a painful chronic condition that is highly prevalent and disabling [1-3]. In 2008, the prevalence was estimated to be 13.9% among adults aged 25 years and older and 33.6% in those aged 65 years and older, corresponding to approximately 27 million Americans with knee osteoarthritis (KOA) [4]. KOA prevalence is rising [5], as are rates of total knee replacement [6,7]. The direct and indirect economic costs of treating KOA are substantial and include surgical costs associated with TKA, disability among people with painful and limiting disease, and loss of work-related productivity. Direct and indirect lifetime costs for persons diagnosed with KOA were estimated in 2013 to be US $140,300 [8].

Nonpharmacologic therapies (eg, exercise) are first-line treatments for KOA due to their benefits and low risks, particularly compared with opioids and invasive procedures [9-12]. Pharmacologic therapies are also frequently used for KOA but carry well-described adverse effects [13]. When these therapies yield insufficient improvement in symptoms, patients may undergo invasive procedures, such as injections using corticosteroids or viscosupplementation, or surgical procedures, such as total knee arthroplasty (TKA), all of which are associated with risks of AEs. Nondrug treatments (eg, exercise) often require long-term adherence, making patient engagement a key factor in their effectiveness and durability. Similar nondrug treatments are also used in post-TKA rehabilitation as an integral part of postprocedure recovery.

Extended reality (XR) technology can deliver pain interventions using various clinical mechanisms, including pain self-management education, psychological skills, passive distraction, and digitally augmented exercise (DAE) [14,15]. The popularity of XR technologies, decreasing costs, increased availability, and innovations in XR hardware and software have contributed to increased interest in this relatively new application. XR can be categorized according to the level of immersion experienced by the user in the digital environment [15]. Virtual reality (VR) presents the highest (full) level of immersion within an interactive digital environment and describes a state in which the user is fully enclosed within an artificial virtual space in which the visual sense is sealed off from the physical environment [15]. For example, users may undergo guided relaxation training taking place in digitally created settings, such as a peaceful forest or relaxing seaside environment, that are visually separated from the real-world physical environment. Full immersion is typically achieved with the use of a headset. Nonimmersive technologies have been used to facilitate DAE and have historically been evaluated in the context of XR but do not incorporate the user’s physical world into the visual experience and are referred to as interactive digital rehabilitation (IDR) for the purposes of this report. These screen-based technologies are best showcased in gaming systems like Microsoft Kinect and Nintendo Wii (Nintendo Co, Ltd) [16]. XR interventions have demonstrated utility for reducing acute pain through distraction (eg, during dental procedures) [17], but whether these interventions improve outcomes when integrated into treatment plans for chronic musculoskeletal pain conditions remains unclear. Prior systematic reviews of XR for KOA [18,19] and post-TKA rehabilitation [20,21] have often pooled heterogeneous XR technologies without stratifying results by level of immersion. Results at varying follow-up durations have also been pooled, and there has been a lack of systematic evaluation of adverse events (AEs). An updated synthesis is warranted given the rapid expansion of the XR evidence base in recent years and the increasing availability of XR devices in clinical and home settings.

### Key Questions

In this review, the objective was to synthesize available evidence on the benefits and harms of XR interventions for the treatment of chronic KOA pain and symptomatic recovery after TKA, with results stratified by level of immersion (VR vs IDR), clinical mechanism, and follow-up duration and with certainty of evidence summarized using the Grading of Recommendations Assessment, Development, and Evaluation (GRADE) criteria.

We present findings on KOA and post-TKA that were part of a larger systematic review undertaken to evaluate the evidence on XR therapies for treating chronic and acute pain conditions [22]. Thus, the results included here focus on the following key question: What are the benefits and harms of XR interventions for the treatment of chronic knee pain due to osteoarthritis or for recovery after TKA?

## Methods

### Registration

A preregistered protocol for the larger review can be found on PROSPERO (International Prospective Register of Systematic Reviews; registration number CRD42023439903). This review is reported in accordance with the PRISMA (Preferred Reporting Items for Systematic Reviews and Meta-Analyses) 2020 statement [23], with search reporting following the PRISMA-S (Preferred Reporting Items for Systematic Reviews and Meta-Analyses Literature Search Extension) [24]. The PRISMA 2020 and PRISMA-S checklists are provided as Checklist 1. There were no deviations from the registered protocol with respect to the eligible populations, interventions, comparators, outcomes, or analytic approach reported in this manuscript.

### Eligibility Criteria

Eligible studies evaluated XR interventions for adults with symptomatic KOA or those who had undergone TKA. Prioritized outcomes were pain-related functioning or interference, pain intensity or severity, and AEs. Other eligible outcomes included pain global change, pain catastrophizing, and kinesiophobia, quality of life, opioid dose or use, physical performance, and adherence. AEs were defined broadly as any untoward medical event occurring during or following the intervention and, for XR interventions, specifically included cybersickness (nausea, dizziness, eye strain, or disorientation related to visual-vestibular mismatch), motion sickness, musculoskeletal injury, and falls. All results compatible with each outcome as they pertained specifically to KOA or post-TKA recovery at any timepoint were sought.

### Database Searches and Article Selection

MEDLINE, Embase, Scopus, CINAHL, and PsycINFO were searched from inception to May 2023 using subject headings and keywords for VR, exergaming, and pain, along with additional terms for conditions in which pain is a predominant symptom (eg, arthritis). Duplicate records were removed prior to abstract screening using deduplication tools in EndNote (Clarivate) [25] and DistillerSR (Evidence Partners) [26]. Titles and abstracts were screened in DistillerSR by 2 separate reviewers (2 separate reviewers for each reference: combination of CK, DDP, DE, CC, MA, AL, KU, NZ) with agreement from both required to exclude. Included abstracts were moved to full-text review, with eligibility again requiring consensus of 2 separate reviewers. Disagreements were resolved with input from additional team members. Excluded references are shown in Multimedia Appendix 1. Database searches were not adapted from any previous review. Relevant systematic reviews identified during the database searches were hand-searched for additional articles. No search peer-review process was undertaken, and no additional sources were purposively searched or consulted. Searches were updated in December 2025 using the original search terms, with the exception of the population of interest, which was narrowed to focus solely on KOA and TKA. Multimedia Appendices 2 and 3 provide detailed study eligibility criteria and complete search strategies. The study screening process and literature flow are depicted using the PRISMA 2020 flow diagram [23].

### Data Abstraction

Data were abstracted by 1 reviewer and verified by a second reviewer. Abstracted data included participant characteristics and inclusion/exclusion criteria; intervention characteristics (technology and devices used, content and goals of intervention); study design and settings; and findings for primary and secondary outcomes (eg, baseline and follow-up means and SDs, change in scores and SDs of changes, and *P* values calculated by studies). Data were abstracted into forms constructed in DistillerSR.

### Risk of Bias Assessment

**Figure 1.**
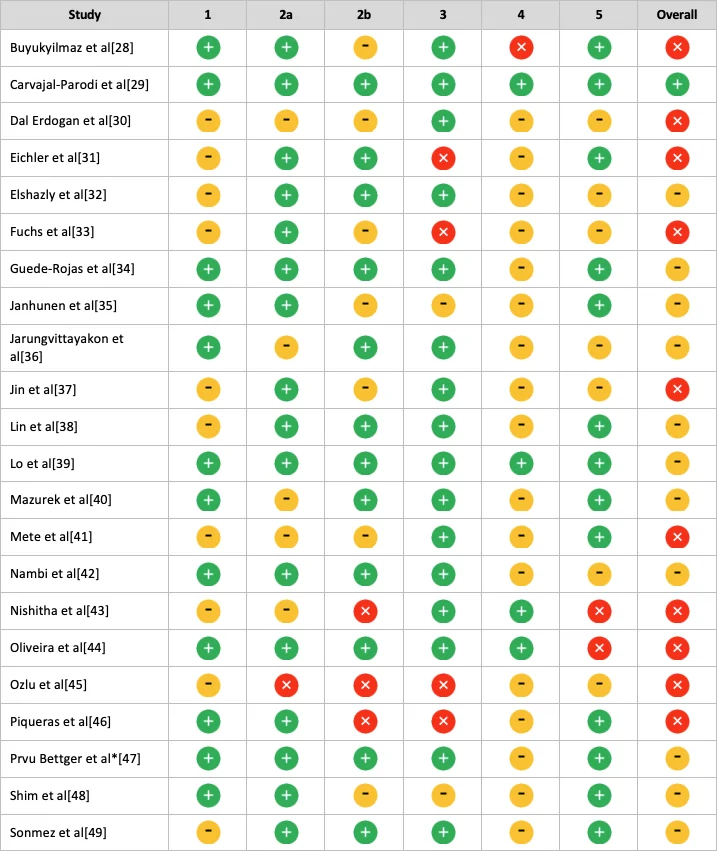
Patient-reported outcome measures reported by included studies [28-49].

Risk of bias (RoB) assessments were conducted independently by 2 researchers using the Cochrane Risk of Bias 2.0 tool [27], which was also adapted to DistillerSR. Discrepancies were resolved by consensus, with input from other parties for resolution as needed. RoB assessments for each included study [28-49] are shown in Figure 1.

### Synthesis and Effect Measures

We synthesized findings from eligible studies qualitatively and, where ≥3 sufficiently similar studies provided appropriate outcome data at comparable time points, performed random-effects meta-analyses using the Hartung-Knapp-Sidik-Jonkman method [50-52] using *meta, metafor,* and *pimeta* packages and R (version 4.3.1; R Foundation for Statistical Computing) to generate forest plots [53]. We examined intervention, comparator, and participant characteristics to determine whether studies were similar, including first grouping studies by clinical indication (ie, osteoarthritis, knee pain, or post-TKA), intervention classification as VR or IDR (according to the framework proposed by Rauschnabel et al [15] and Spiegal et al [16]) and clinical mechanism (distraction or DAE). Distraction refers to the effect of directing attentional resources of the user away from unpleasant symptoms or stimuli, involving techniques such as music, imagery, or relaxation [54,55]. DAE entails an interactive process through which the user is given instruction or a demonstration of a target exercise before performing the exercise themselves [46]. Author-reported XR equipment and devices are shown in Multimedia Appendix 4.

For efficacy outcomes (measures described in Table 1), we assessed between-group differences in mean changes of continuous outcomes (ie, difference in change scores [Diff Δ]), preferentially as standardized effect sizes (Diff Δ/SD of change) [56]. To calculate Diff Δ, we subtracted the mean change in the comparator group from the mean change in the XR intervention group (Δ_XR_–Δ_C_). Thus, for outcome measures in which lower scores are better (eg, pain intensity or severity), a negative value for Diff Δ indicates greater improvement in the XR intervention group. Table 1 provides a description of the standardized measures reported by studies and the interpretation of scores (eg, higher score is better or worse).

**Table 1.** Patient-reported outcome measures reported by included studies.

Outcome category Measure name	Scoring range (number of items and domains)
Pain-related functioning	
WOMAC^a^	0‐96 (lower is better) 24 items (3 domains: physical function; pain; and stiffness)
KOOS^b^	0‐100 (higher is better) 42 items (5 domains: pain, ADL^c^, sports and recreation, QoL^d^, and symptoms)
OKS^e^	0‐48 (higher is better) 12 items
Pain severity or intensity	
VAS^f^	0‐10 (lower is better)
NRS^g^	0‐10 (lower is better)
Health-related quality of life	
WHOQOL-BREF^h^	0‐100 (higher is better) 26 items (4 domains: physical health; psychological health; social relationships; and environment)
CDC^i^ Health-Related Quality of Life	Multiple indices
EQ-5D	0‐1 (higher is better)
Kinesiophobia	
TSK^j^	17‐68 (lower is better) 17 items (4 domains: fear of injury, dysfunctional self, perceived danger for heart problems, and avoidance of exercise)

a WOMAC: Western Ontario and McMaster Universities Osteoarthritis Index.

b KOOS: Knee Injury and Osteoarthritis Outcome Score.

c ADL: activities of daily living.

d QoL: quality of life.

e OKS: Oxford Knee Score.

f VAS: Visual Analog Scale.

g NRS: Numeric Rating Scale.

h WHOQOL-BREF: World Health Organization Quality of Life Brief Version.

i CDC: Centers for Disease Control and Prevention.

j TSK: Tampa Scale for Kinesiophobia.

In determining whether there were between-group differences in efficacy outcomes, we preferentially examined standardized Diff Δ and applied recommended thresholds (eg, <0.2 indicates no between-group difference) [56]. If standardized Diff Δ was not reported and could not be calculated (eg, due to lack of SD or confidence intervals), we considered study analyses and interpretations of findings, as well as actual Diff Δ values, to determine whether there were between-group differences. No sensitivity analyses were conducted.

### Reporting Bias Assessment

Reporting bias was not formally assessed quantitatively (eg, funnel plot asymmetry tests) because no individual meta-analytic comparison included ≥10 studies, below which such tests are considered unreliable [57]. We instead considered the potential for missing studies qualitatively, including inspection of trial registries and reference lists of prior systematic reviews identified during the searches.

### Certainty of Evidence Assessments

We assessed certainty of evidence (COE) for 3 prioritized outcomes, including pain-related functioning or interference, pain intensity or severity, and AEs. We rated the COE for these outcomes separately for VR and IDR interventions by clinical mechanism (DAE, distraction, or psychoeducation) and by condition (ie, KOA pain and post-TKA pain and rehabilitation). We used GRADE methodology [58] to rate overall COE as high, moderate, low, or very low (Multimedia Appendix 5). For each prioritized outcome, we used the GRADEpro Guideline Development Tool (GDT) [59] to systematically evaluate 5 domains: study limitations, imprecision, inconsistency, indirectness, and other considerations. For presentation of anticipated absolute effects, data from exemplar studies used to illustrate the range and direction of findings at time points were selected on the basis of (1) lower RoB ratings and (2) the largest sample size. Certainty of evidence tables are found in Multimedia Appendices 6 and 7.

## Results

### Overview of Included Studies

**Figure 2.**
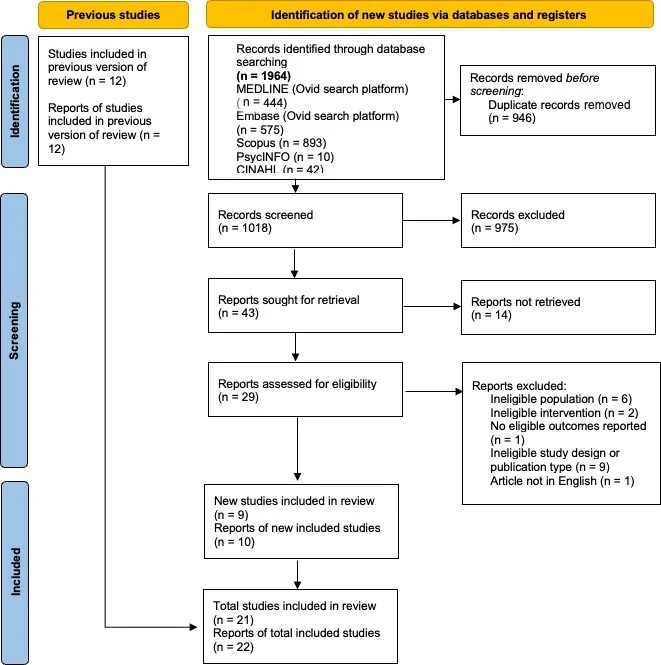
Study selection flow diagram. ISRCTN: International Standard Randomized Controlled Trial Number; TKA: total knee arthroplasty.

We identified 21 studies [28-49] (reported in 22 publications) that evaluated the use of XR interventions for patients with KOA (k=12) or post-TKA (k=9; Figure 2), as summarized in Table 2. The studies were mostly small to moderate in size (k=18 with a total number of participants n≤100), with a few that were larger (k=2 with n=101‐200 and k=1 with n>200). Follow-up duration ranged from 2 weeks to 6 months with most studies (k=16) having ≤3 months of follow-up. The primary clinical mechanism was consistent throughout the studies, with 19 studies [28-32,34-39,41-49] using DAE and 1 study [33] using distraction and 1 [40] using psychoeducation. No studies reported on pain, global change, or opioid use. All but one study [47] was conducted outside of the United States (k=20), and most included middle-aged (k=10 aged 30‐64 years) and older adults (k=7 aged ≥65 years). One study included only young adults with posttraumatic osteoarthritis [42], and mean age was not reported in k=3 studies [39,43,46]. Studies were rated as having a high RoB (k=10) [28,30,31,33,37,41,43-46], some concerns for RoB (k=11) [32,34-36,38-40,42,47-49], or a low RoB (k=1) [29]. Detailed RoB assessments are shown in Figure 1. Detailed trial characteristics are found in Multimedia Appendices 8 and 9.

**Table 2.** Characteristics of studies on knee osteoarthritis (KOA) and rehabilitation posttotal knee arthroplasty (TKA).

Characteristic and subcategory		KOA^a^ VR^b^ (k=2)	KOA IDR^c^ (k=10)	Post-TKA^d^ VR (k=4)	Post-TKA IDR (k=5)
Mechanism of XR^e^ intervention					
Distraction		—^f^	—	1 (25)	—
Psychoeducation		—	—	1 (25)	—
Digitally augmented exercise (DAE^g^)		2 (100)	10 (100)	2 (50)	5 (100)
Outcomes reported					
Pain-related functioning or interference		2 (100)	9 (90)	4 (100)	4 (80)
Pain intensity or severity		2 (100)	8 (80)	4 (100)	4 (80)
Adverse events		1 (50)	3 (30)	—	1 (20)
Quality of life		1 (50)	2 (20)	—	3 (60)
Kinesiophobia/pain catastrophizing		—	1 (10)	—	—
Physical performance		1 (50)	8 (80)	3 (75)	5 (100)
Adherence		1 (50)	1 (10)	—	—
Sample size					
<50		—	3 (30)	1 (25)	—
50‐75		1 (50)	6 (60)	3 (75)	2 (40)
76‐100		1 (50)	1 (10)	—	—
101‐200		—	—	—	2 (40)
>200		—	—	—	1 (20)
Region					
Europe		1 (50)	4 (40)	1 (25)	3 (60)
North America		—	—	—	1 (20)
Middle East		—	2 (20)	1 (25)	—
Asia		1 (50)	2 (20)	2 (50)	1 (20)
South America		—	2 (20)	—	—
Follow-up duration (days)					
<30 days		1 (50)	5 (50)	1 (25)	—
30‐90 days		1 (50)	5 (50)	2 (50)	3 (60)
>90 days		—	—	1 (25)	2 (40)
Mean/median age (years)					
<30		—	1 (10)	—	—
30‐64		1 (50)	8 (80)	—	1 (20)
≥65		—	1 (10)	3 (75)	4 (80)
Not Reported		1 (50)	—	1 (25)	—

a KOA: knee osteoarthritis.

b VR: virtual reality.

c IDR: interactive digital rehabilitation.

d TKA: total knee arthroplasty.

e XR: extended reality.

f Not applicable.

g DAE: digitally augmented exercise.

Below, we provide results first for KOA studies and then for rehabilitation post-TKA. Findings for each condition are grouped according to intervention type (VR or IDR) and clinical mechanism. Detailed results are found in Multimedia Appendices 10 and 11.

### KOA

Twelve trials evaluated XR interventions for chronic knee pain due to osteoarthritis. Studies involved VR (k=2) [39,45] or IDR (k=10) [28-30,32,34,36,38,41,42,44,49] interventions compared with conventional therapy. Trial characteristics and main findings are summarized in Table 3.

**Table 3.** Summary of findings for knee osteoarthritis interventions. Western Ontario and McMaster Universities Osteoarthritis Index (WOMAC) scoring scales vary across included studies (eg, 0-96 Likert, 0-240, or 0-2400 visual analog/numerical); raw difference-in-change (Diff Δ) values for WOMAC are therefore not directly comparable across studies using different scale versions. Standardized mean differences used in the meta-analyses are unaffected by scale version.

Author, Year; Country; RoB^a^	Key participant characteristics	Study arms, N randomized (N analyzed)	Setting and duration	Outcomes			
				Pain-related functioning	Pain intensity or severity	Adverse events	Other eligible outcomes^b^
VR intervention trials							
Lo et al [39]; China; some concerns	Knee osteoarthritis (ACR^c^ criteria), moderate-to-severe knee pain for ≥3 months; mean age NR^d^; 33%-43% women	2 Arms: Smartphone VR app capturing outdoor garden scenes via headset, plus standard care; N=15 (15) Standard care; N=15 (15)	Home; 12 weeks	WOMAC^e^ (total) Baseline mean (SD): VR: 902.9 (454.8) Comparator: 752.0 (549.3) Diff Δ^f^: 6-wk: 82.6; *P*=.66 12-wk: –61.3; *P*=.69	NRS^g^ Baseline mean (SD): VR: 5.9 (1.9) Comparator: 4.8 (1.5) Diff Δ^f^: 6-wk: –0.04; *P*=.95 12-wk: –0.42; *P*=.64^g^	Cybersickness: 5 events in VR^h^ group	Quality of Life EQ-VAS^i^
Özlü et al [45]; Turkey; high	Knee osteoarthritis, Kellgren-Lawrence grade 2-3 (ACR criteria); mean age 53-54 years; 49%-68% women	2 Arms: Oculus headset games focusing on lateral movements and trunk flexion to interact with virtual targets, and therapeutic ultrasound and TENS^j^ treatment; N=41 (35) Conventional treatment, ultrasound, and TENS; N=41 (38)	Clinic; 3 weeks	WOMAC (total) Baseline mean (SD): VR: 31.7 (6.8) Comparator: 33.0 (7.9) Diff Δ^f^^,^^k^: 3-wk: –8.7 7-wk: –5.1	VAS Baseline mean (SD): VR: 5.6 (0.9) Comparator: 5.8 (0.7) Diff Δ^f^: 3-wk: –0.7 7-wk: –1.1	NR	Physical performance 6-minute walk Berg Balance Scale
IDR^l^ intervention trials							
Buyukyilmaz et al [28]; Turkey; high	Knee osteoarthritis (ACR criteria), Kellgren-Lawrence grade 2‐3, osteoarthritis-induced pain ≥6 months; mean ages 59‐60, 100% women	2 Arms: Becure Balance System (Wii-based exergame with balance board), plus conventional PT^m^; N=28 Conventional PT; N=28	Clinic; 8 weeks	WOMAC (total) Baseline mean (SD): VR: 47.1 (12.1) Comparator: 53.0 (11.2) Diff Δ^f^ 4-wk: NR 8-wk: NR	NR	NR	Physical performance ROM^n^ knee flexion Knee flexion/extension strength (kg-force) Joint position sense (15°, 45°, and 75°) TUG
Carvajal-Parodi et al [29,34]; Chile; low	Mild-to-moderate KOA and/or HOA (ACR criteria), Kellgren-Lawrence grade 2‐3; mean age 69; 83% women	2 Arms: Nintendo Switch exergame on 43-in television; N=30 Comparator; N=30	Clinic; 4 weeks	NR	VAS Baseline mean (SD): VR: 46.7 (19.7) Comparator: 46.3 (24.1) Diff Δ^f^: 4-wk: NR	“No adverse events were reported”	Physical performance TUG
Dal Erdogan et al [30]; Turkey; high	Knee osteoarthritis, Kellgren-Lawrence stage 2‐3, mean ages 60‐61, 78% women	2 Arms: BTS Nirvana virtual reality rehabilitation device analyzing patient movements; N=18 Comparator; N=18	Clinic; 3 weeks	WOMAC (total) Baseline mean (SD): VR: 65.0 (6.9) Comparator: 61.9 (7.9) Diff Δ^f^: 3-wk: NR WOMAC (pain) Baseline mean (SD): VR: 10.3 (1.8) Comparator: 10.3 (1.8) Diff Δ^f^: 3-wk: NR	VAS (movement) Baseline mean (SD): VR: 6.1 (1.0) Comparator: 6.4 (1.2) Diff Δ^f^: 3-wk: NR VAS (rest) Baseline mean (SD): VR: 6.1 (1.2) Comparator:6.1 (1.2) Diff Δ^f^: 3-wk: NR	NR	NR
Elshazly et al [32]; Saudi Arabia; some concerns	≥3 mo osteoarthritis, can walk ≥30 ft without assistance, and not in any sports or PT; mean ages 58‐60, women NR	3 Arms: Game involving standing and taking steps on virtual platform (device NR), N=20 (20) Sensorimotor training (SMT), N=20 (20) Walking program, N=20 (20)	Clinic; 8 weeks	WOMAC (total) Baseline mean (SD): IDR: 71.7 (3.4) SMT—71.7 (2.8) Walking: 71.9 (3.1) Diff Δ (IDR-SMT)^f^: 4-week: –13.5 8-week: –19.5 Diff Δ (IDR-Walking)^f^: 4-week: –14.1 8-week: –29.9	VAS Baseline mean (SD): IDR: 6.8 (0.9) SMT: 6.6 (1.2) Walking: 6.68 (0.84) Diff Δ (IDR–SMT)^f^: 4-week: –1.9 8-week: –1.8 Diff Δ (IDR-Walking)^f^: 4-week: –1.8 8-week: –2.0	NR	Quality of life CDC^o^ Health Related Quality of life Physical performance position sense
Jarungvittayakon et al [36]; Thailand; some concerns	Knee osteoarthritis (ACR criteria), Kellgren-Lawrence grade 2‐3; mean ages 59.9‐62.1; 79%‐82% women	2 Arms: Wearable sensor exergame (mobile app with knee sensors for airplane game requiring knee flexion/extension), plus standard conservative treatment; N=28 Standard conservative treatment (quadriceps strengthening, medications, self-care advice); N=28	Home; 6 weeks	KOOS^p^ Baseline mean (SD): VR: 63.0 (16.9) Comparator: 65.4 (17.2) Diff Δ^f^: 6-wk: 7.5	VAS (in motion) Baseline mean (SD): VR: 5.5 (2.0) Comparator: 6.5 (2.4) Diff Δ^f^: 6-wk: −1.3, *P*=.0002 VAS (at rest) Baseline medians: VR: 2 (IQR NR) Comparator: 2 (IQR NR) Diff Δ^f^: 6-wk: –1.0; *P*=.025	NR	Physical performance ROM Flexion ROM Extension Arc of motion TUG
Lin et al [38]; Taiwan; some concerns	Knee osteoarthritis (ACR criteria), Kellgren and Lawrence grade ≥2, able to walk >15 m, and not needing NSAIDs^q^; mean ages 56‐58, 43‐60% female	2 Arms Games involving interaction with virtual targets through lower limb and trunk movements (via sensor pad for feet), in addition to temperature therapy and TENS; N=40 (40) Temperature therapy, TENS, and conventional exercise program (stretching, stabilization exercises, etc); N=40 (40)	Clinic; 4 weeks	WOMAC (physical function) Baseline mean (SD): Intervention: 505.1 (328.4) Comparator: 581.0 (383.8) Diff Δ^f^: 2-week: 39.1 4-week: 60.3 8-week: 90.0 16-week: 82.8	WOMAC (pain) Baseline mean (SD): Intervention: 161.2 (114.7) Comparator: 170.2 (121.3) Diff Δ^f^: 2-week: 0.7 4-week: 3.9 8-week: –19.9 16-week: 4.9	“No adverse effects observed in either group”	Quality of life WHOQOL^r^-BREF Physical performance Biodex stability system 10 meter walk time Stair ascent, descent time
Mete and Sari [41]; Turkey; high	Knee osteoarthritis, Kellgren and Lawrence grade 2‐3; median ages 57‐60, 77‐88% women	2 Arms Games involving control of on-screen avatars through knee flexion and extension, via special device (MarVAJED) with sensors for joint positions and provided auditory and visual feedback, in addition to comparator treatment; N=32 (30) Conventional treatment with ultrasound, TENS, temperature therapy, and muscle strengthening exercises; N=32 (30)	Clinic; 6 weeks	WOMAC (total) Baseline medians (IQR): Intervention: 19.7 (18.2-21) Comparator: 15.1 (9.3-18) Diff Δ: not calculable^s^	WOMAC (pain) Baseline medians (IQR): Intervention: 6 (5.37-7.12) Comparator: 4.5 (4.3-6) Diff Δ not calculable^s^ VAS (at rest) Baseline medians (IQR): Intervention: 32.2 (20.8-40.0) Comparator: 36.3 (30.0-40.0) Diff Δ not calculable^s^	NR	Kinesiophobia TSK^t^ Physical performance Pedalo Balance Score Knee flexion and extension ROM Knee proprioception at 30°, 60° Peak torque of knee flexion and extension at 120° and 240°
Nambi et al [42]; Saudi Arabia; some concerns	Male soccer players with posttraumatic osteoarthritis ≥3 months following ACL^u^ injury (verified by orthopedic surgeon) and pain rating 4‐8; mean ages 22‐23, sex/gender not reported	3 Arms: Games using ProKin system that required knee movements to interact with visual targets; N=20 (18-20) Sensorimotor training (SMT); N=20 (18-20) Control: N=20 (19-20)	Clinic; 4 weeks	WOMAC (total) Baseline mean (SD): Intervention: 72.3 (4.2) SMT: 72.5 (4.5) Control: 71.2 (3.8) Diff Δ (VR–SMT)^f^: 4-week: –22.1 8-week: –9.8 3-month: –14.0 Diff Δ (VR–control)^f^: 4-week: –29.3 8-week: –31.0 3-month: –25.1	VAS Baseline mean (SD): Intervention: 7.2 (0.5) SMT: 7.4 (0.4) Control: 7.3 (0.4) Diff Δ (VR–SMT)^f^: 4-week: –2.3 8-week: –0.8 3-month: –0.8 Diff Δ (VR–control)^f^: 4-week: –3.1 8-week: –1.6 3-month: –3.2	NR	NR
Oliveira et al [44]; Brazil; high	Knee osteoarthritis, Kellgren-Lawrence grades 2‐4, independent ambulation; mean ages 62‐63, 70%‐85% women	2 Arms: Xbox 360 with Microsoft Kinect 360 sensor training; N=20 Kinesiotherapy plus postural exercises; N=20	NR; 8 weeks	WOMAC (total) Baseline mean (SD): VR: 36.2 (13.4) Comparator: 38.4 (14.4) Diff Δ^f^: 8 wk: -0.6	VAS Baseline mean (SD): VR: 7.5 (1.2) Comparator: 8.0 (1.5) Diff Δ^f^: 8-wk: 0.2	NR	Physical performance Anticipatory postural adjustments (latency, amplitude, and time to max)
Sonmez et al [49]; Turkey; some concerns	Knee osteoarthritis, Kellgren-Lawrence grade 1‐3, able to walk >15 m; mean age 60.1 (both groups), 75% women	2 Arms: Microsoft Kinect Xbox 360 exergame; N=20 Physiotherapy plus hot pack, TENS; N=20	Clinic; 3 weeks	WOMAC (function) Baseline mean (SD): VR: 19.5 (5.9) Comparator: 20.4 (4.2) Diff Δ^f^: 3 wk: −4.7	WOMAC (pain) Baseline mean (SD): VR: 6.3 (3.5) Comparator: 7.5 (4.4) Diff Δ^f^: 3-wk: −0.8	“No adverse events were observed in either group”	Physical performance TUG^v^ Proprioception (right and left)

a RoB: risk of bias.

b Results for other eligible outcomes are found in Multimedia Appendix 10.

c ACR: American College of Rheumatology.

d NR: not reported.

e WOMAC: Western Ontario and McMaster Universities Osteoarthritis Index.

f Diff Δ calculated by review team, unable to standardize as no SD for change reported.

g NRS: numeric rating scale.

h VR: virtual reality.

i VAS: Visual Analog Scale.

j TENS: transcutaneous electrical nerve stimulation.

k Diff Δ: difference in change scores.

l IDR: interactive digital rehabilitation.

m PT: physical therapy.

n ROM: range of motion.

o CDC: Centers for Disease Control and Prevention.

p KOOS: Knee Injury and Osteoarthritis Outcome Score.

q NSAIDs: nonsteroidal anti-inflammatory drugs.

r WHOQOL-BREF: World Health Organization Quality of Life Brief Version.

s Diff Δ not reported and cannot be calculated using provided result.

t TSK: Tampa Scale for Kinesiophobia.

u ACL: anterior cruciate ligament.

v TUG: Timed Up and Go test.

#### VR-DAE

VR-DAE may result in better pain-related functioning and greater decreases in pain intensity when compared with conventional therapy, though evidence is inconsistent across studies (Multimedia Appendix 6). Two studies [39,45] evaluated VR-DAE versus conventional therapy for KOA pain, both using head-mounted displays to deliver interactive exercise in virtual environments. Özlü et al [45] (Turkey, some concerns RoB; N=82) compared 3 weeks of Oculus VR gaming (Fish Game and Monkey Game targeting balance and proprioception via lateral trunk flexion and stepping, 15 minutes/session, 5 days/week) plus conventional therapy to conventional therapy alone (therapeutic ultrasound and transcutaneous electrical nerve stimulation [TENS]). Lo et al [39] (China, some concerns RoB; N=60) evaluated a 12-week home-based smartphone VR application (VRiKnee; VR Shinecon headset) delivering guided quadriceps strengthening exercises with visual feedback (virtual flowers blooming with successful movement) compared with the same exercises without VR.

Özlü et al [45] reported that the VR group showed greater improvement in Western Ontario and McMaster Universities Osteoarthritis Index (WOMAC) total at 3 weeks (Diff Δ −8.7 [low COE]) and 7 weeks (Diff Δ −5.0), in contrast with Lo et al [39], who presented WOMAC total scores showing no difference between groups at either 6 weeks (Diff Δ 82.6 [very low COE]) or 12 weeks (Diff Δ −61.3 [low COE]), with confidence intervals crossing zero at both time points. With respect to pain intensity, Visual Analog Scale (VAS) scores in the study by Özlü et al [45] favored the VR group at 3 weeks (Diff Δ −0.7 [low COE]) and 7 weeks (Diff Δ −1.1). Lo et al [39] reported pain scores using NRS and found no difference at 6 weeks (Diff Δ −0.04 [very low COE]) or 12 weeks (Diff Δ −0.42 [moderate COE]). Lo et al [39] reported 5 events of cybersickness in the VR group; no other AEs were assessed. Özlü et al [45] did not report on AEs. For nonprioritized outcomes, Özlü et al [45] assessed physical performance and found inconsistent results: no difference between groups in the 6-minute walk test (Diff Δ −0.9 m at both 3 and 7 weeks) but greater improvement in the VR group on the Berg Balance Scale at both time points (Diff Δs of 1.9 and 2.9, respectively). Additionally, Lo et al [39] assessed quality of life (EQ-VAS), finding no between-group differences (Table 3).

#### IDR-DAE

IDR may provide a medium-term benefit on pain-related functioning (very low to low COE) and pain intensity (low COE) compared with conventional therapy, but results in little to no difference in reported AEs (low to high COE), and the evidence for early and late time points was very uncertain (Multimedia Appendix 6). Ten studies presented in 11 publications [28-30,32,34,36,38,41,42,44,49] evaluated IDR for pain due to KOA. IDR programs lasted 3‐10 weeks and compared IDR interventions using Microsoft Kinect (k=3) [32,44,49], Nintendo Switch (k=1) [29], Wii Balance Board (k=1) [28], or proprietary sensor-based systems (k=5) [30,36,38,41,42] versus conventional exercise therapies (Table 3). Studies were small (total n=40‐80) and enrolled primarily middle-aged to older adults with primary KOA, with the exception of one that enrolled young male athletes with posttraumatic osteoarthritis [42]. Nine studies [28-30,32,34,38,41,42,44,49] were clinic-based and one was home-based [36]. Studies were rated high (k=4) [28,30,41,44], some concerns (k=5) [32,36,38,42,49], or low (k=1) [29] RoB.

**Figure 3.**
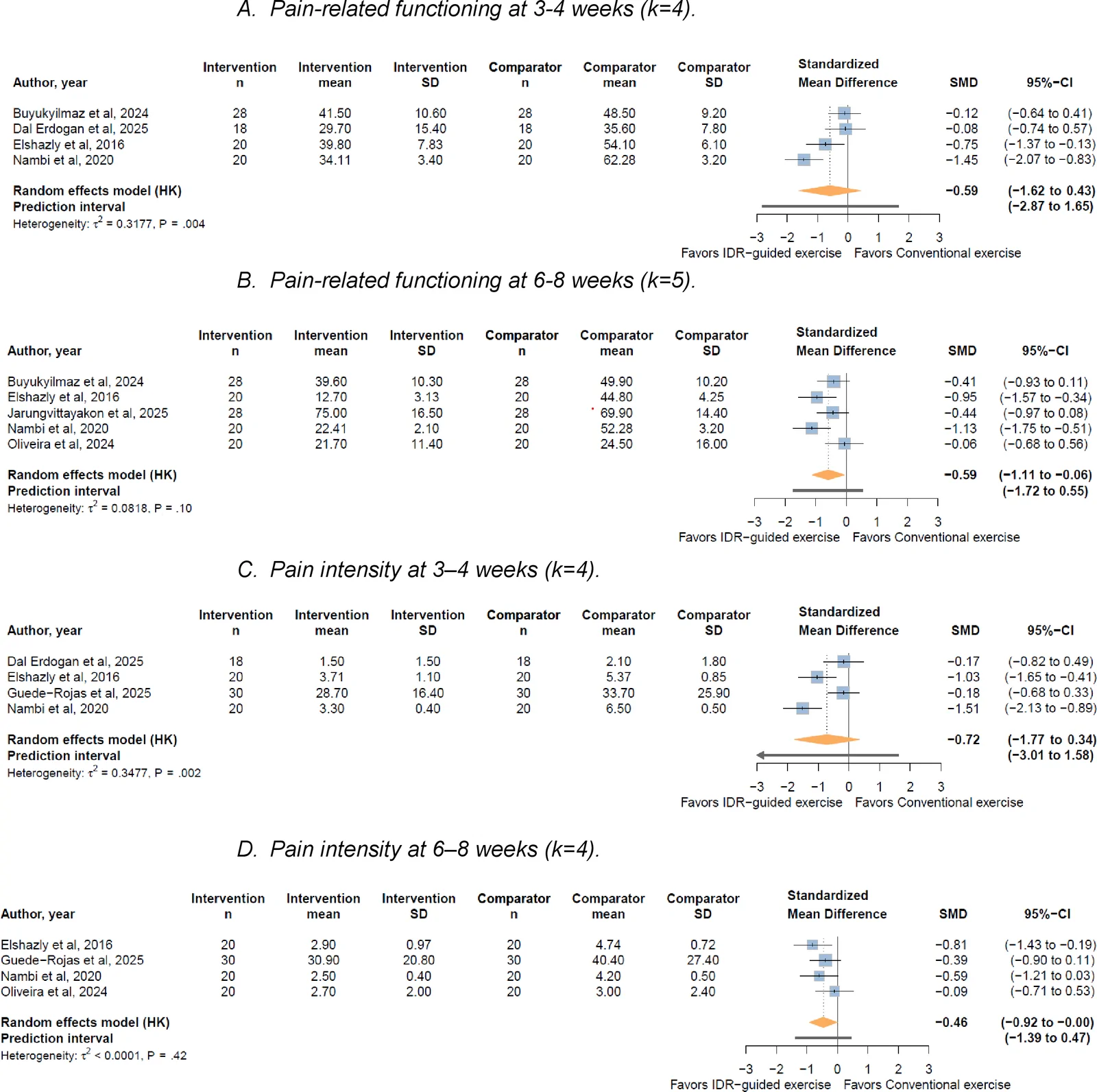
Forest plots of random-effects meta-analyses of interactive digital rehabilitation (IDR) versus conventional exercise for knee osteoarthritis. IDR: interactive digital rehabilitation; SMD: standardized mean difference [28,30,32,34,36,42,44].

Nine studies [28,30,32,36,38,41,42,44,49] assessed pain-related functioning using WOMAC total (k=7), WOMAC domain scores (k=1), or Knee Injury and Osteoarthritis Outcome Score (KOOS; k=1). Pooled results (Figure 3) showed a nonsignificant trend favoring IDR (SMD −0.59, 95% CI −1.62 to 0.43; PI −2.87 to 1.65) at 3‐4 weeks (very low COE) and a small improvement in pain-related functioning (SMD −0.59, 95% CI −1.11 to −0.06; PI −1.72 to 0.55) at 6‐8 weeks (low COE). Three IDR studies were not included in the meta-analyses for pain-related functioning due to incompatible outcome measures or reporting. Lin et al (Taiwan, some concerns RoB; N=80) [38] reported WOMAC domain scores (not total), showing inconsistent results over 16 weeks. Mete and Sari (Turkey, high RoB; N=64) [41] reported only medians (IQR), precluding Diff Δ calculation. Sonmez et al (Turkey, some concerns RoB; N=40) [49], which compared against sham VR control, reported WOMAC-pain (Diff Δ −0.8) and function (Diff Δ −4.7) subscale scores at 3 weeks but not a WOMAC total score.

Nine studies [29,30,32,36,38,41,42,44,49] assessed pain intensity using VAS (k=7) or the WOMAC-pain subscale (k=1). In pooled analysis at 3‐4 weeks (k=4), IDR showed a moderate-to-large reduction in pain intensity (very low COE), favoring IDR (SMD −0.72, 95% CI −1.77 to 0.34; PI −3.01 to 1.58). At 6‐8 weeks (k=4; low COE) it showed a small reduction in pain intensity favoring IDR (SMD −0.46, 95% CI −0.92 to 0.00; PI −1.39 to 0.47). Jarungvittayakon et al [36] reported greater VAS reduction in the IDR group during motion (Diff Δ −1.3, *P*=.0002) and at rest (Diff Δ −1.0, *P*=.025). Guede-Rojas et al [34] reported VAS Diff Δ −9.9 at 6 weeks, sustained at 14 weeks (Diff Δ −6.9).

Three studies [29,38,49] reported on AEs, with each reporting that none were observed (low to high COE) [29,38,49]. The remaining 7 studies [28,30,32,36,41,42,44] did not address AEs, representing a significant evidence gap given the physical demands of exergaming in an older population. For other outcomes, Mete and Sari [41] assessed kinesiophobia using the Tampa Scale of Kinesiophobia but only provided medians (IQRs). Two studies examined quality of life: Elshazly et al [32] using the CDC Health-Related Quality of Life scale and Lin et al [38] using the World Health Organization Quality of Life Brief Version (WHOQOL-BREF). There were generally small improvements in all groups with no clear between-group differences (eg, Diff Δ range 0.6 to 1.9 in the WHOQOL-BREF physical domain at 16 weeks [38]). Eight studies [28,29,32,36,38,41,44,49] evaluated physical performance using various measures including the 10-meter walk test, balance and position sense, range of motion (ROM), the Timed Up and Go test (TUG), strength dynamometry, and anticipatory postural adjustments. All groups generally improved, but results were inconsistent regarding which intervention group showed greater improvement. One study, Carvajal-Parodi et al [29] (Chile, low RoB; N=60), reported on an adherence-related outcome with percentages of study participants who had attended ≥20 therapy sessions. Little difference was shown, with 76.6% and 73.3% in IDR and comparator arms reaching this point.

### Rehabilitation After TKA

Nine trials evaluated XR for rehabilitation after TKA. Four trials examined VR interventions, incorporating modalities of DAE [37,43], passive distraction [33], and psychoeducation [40]. Five trials examined IDR [31,35,46-48], all of which evaluated the effectiveness of DAE. Trial characteristics and main findings are summarized in Table 4. Certainty of evidence tables are found in (Multimedia Appendix 7). Below, we first describe findings for VR intervention trials, organized by clinical mechanism, and then present findings for IDR studies.

**Table 4.** Summary of findings for posttotal knee arthroplasty (TKA) interventions. Western Ontario and McMaster Universities Osteoarthritis Index (WOMAC) scoring scales differ across included studies (eg, 0-96 Likert, 0-240, or 0-2400 visual analog/numerical); raw difference-in-change (Diff Δ) values for WOMAC are therefore not directly comparable across studies using different scale versions. Standardized mean differences used in the meta-analyses are unaffected by scale version.

Author, year; country; RoB^a^	Key participant duration characteristics	Study arms: N randomized (N analyzed)	Setting and duration	Outcomes			
Pain-related functioning	Pain intensity or severity	Adverse events	Other eligible outcomes^b^
VR^c^ intervention trials							
Fuchs et al [33]; Israel; high	Patients with osteoarthritis undergoing unilateral TKA^d^; mean age 70 years, 52%‐63% women	2 Arms: Nature or music film watched on Oculus while undergoing continuous passive motion therapy, N=30 (30) Continuous passive motion therapy, N=25 (25)	Hospital; 2 days	Modified KOOS^e^: Baseline mean (SD): Intervention: 36.4 (15.1) Comparator: 34.5 (17.0) Diff Δ^f^ (6 mo)^g^: 1.1	VAS^h^ Baseline median (IQR): Intervention: 6 (5-8) Comparator: 6 (6-8) Diff Δ^i^ NR^j^	NR	NR
Jin et al [37]; China; high	Patients with osteoarthritis undergoing TKA; mean age 66 years, 55%‐61% women	2 Arms: Game using Oculus headset rowing a boat using knee flexion, N=33 (33) Passive flexion of knee using arms, N=33 (33)	Hospital; NR	WOMAC^k^ baseline (SD): Intervention: 45.0 (5.1) Comparator: 44.2 (5.7) Diff Δ^g^: 1-month: –3.9 3-month: –4.7 6-month: –5.6	VAS Baseline (SD): Intervention: 7.4 (1.1) Comparator: 7.4 (1.3) Diff Δ^g^: 3-day: –0.3 5-day: –0.5 7-day: –0.5	NR	Physical performance (3, 7, and 14 d) ROM^l^
Mazurek et al [40]; Poland; some concerns	Age ≥60 years, recent hip or knee joint arthroplasty; mean ages 69.5‐69.7; 62% women	2 Arms: VRTierOne device (HTC Vive headset) with VR rehabilitation sessions; N=34 Comparator; N=34	NR; 4 weeks	Barthel Index Baseline mean (SD): VR: 54.6 (16.5) Comparator: 56.2 (17.4) Diff Δ^g^: 4-week: –21.2; *P*<.001	VAS baseline mean (SD): VR: 5.3 (2.0) Comparator: 4.4 (2.1) Diff Δ^g^: 4-week: –2.6; *P*<.001	NR	Physical performance · Tinetti balance · Rivermead Motor Assessment (Gross Function, Leg/Trunk)
Nishitha et al [43]; India; high	Unilateral TKA, aged 45‐65 years; mean ages NR, sex NR	2 Arms: 3D head-mounted VR with limb sensors for interactive games; N=18 Comparator; N=18	Clinic; 12 weeks	WOMAC (total) baseline mean (SD): VR: 71.2 (0.8) Comparator: 71.0 (0.8) Diff Δ^g^: 9-week: –11.1 12-week: –14.3	NRS^m^ baseline mean (SD): VR: 8.56 (0.5) Comparator: 8.44 (0.5) Diff Δ^g^: 4-week: –0.5 12-week: –0.9	NR	Physical performance · ROM Flexion
IDR^n^ intervention trials							
Eichler et al [31]; Germany; high	Patients with osteoarthritis after TKA or THA^o^; mean ages 53‐57 years; 49%‐54% women	2 Arms: Exercises using Microsoft Kinect sensor, as demonstrated by an avatar, N=56 (48) Usual care, N=55 (39)	Home (after 3 wk inpatient rehab); 3 months	WOMAC (total) Baseline mean (SD) Intervention: 26.4 (18.5) Comparator: 24.8 (16.4) Standardized diff Δ (3 mo)^m^: –0.3	NR	NR	Quality of life (3 mo) SF-36^p^ Physical performance (3 mo) TUG^q^ 6-minute walk test Stair ascend test
Janhunen et al [35]; Finland; some concerns	After first primary TKA; mean ages 66‐67; 63‐64% women	2 Arms: Games using Microsoft Kinect, involved similar movements as home PT^r^, N=25 (21) Standard PT, N=27 (25)	Home; 16 weeks	OKS Baseline (SD): Intervention: 26.7 (6.7) Comparator: 26.9 (6.5) Standardized diff Δ (4 mo)^s^: 0.32; *P*=.27^t^	VAS Baseline mean (SD): Intervention: 57.1 (18.3) Comparator: 54.2 (21.6) Standardized diff Δ (4 mo)^s^: –0.39, *P*=.18^t^	NR	Physical performance (2 and 4 mo) TUG Short physical performance battery Muscle force flexion, extension ROM flexion, extension
Piqueras et al [46]; Spain; high	After primary TKA, with active ROM flexion 80° and extension –10°, without signs of stiffness, and able to walk; mean age 73 years, 72% women	2 Arms: Screen and leg movement sensors to instruct and monitor knee exercises, N=90 (68) Conventional PT^r^, N=91 (65)	Clinic (XR^u^), home (control); 2 weeks	NR	VAS Baseline (SD): Intervention: 3.8 (2.01) Comparator: 4.3 (1.93) Standardized diff Δ^s^: 2-week: –0.05; *P*=.80^t^ 3-month: 0.22; *P*=.28^t^	NR	Physical performance (2-wk and 3-mo) TUG Quadriceps/ hamstring strength ROM flexion, extension
Prvu Bettger et al [47]; United States; some concerns	TKA for nontraumatic conditions and expected to discharge home; mean age 65 years, 60%‐65% women	2 Arms: Virtual telehealth system (VERA^v^) to demonstrate exercises (with avatar) and monitor performance, N=153 (140) Conventional PT care, N=153 (140)	Home; 12 weeks	KOOS Baseline (SD): Intervention: 37.0 (12.0) Comparator: 36.0 (13.0) Diff Δ^g^: 6-week: –1.8 12-week: –0.4	NRS Baseline (SD): Intervention: 5.2 (2.1) Comparator: 5.7 (2.0) Diff Δ 12-week^g^: 0.2	Number of falls (12 wk): Intervention: 19.4% Comparator: 14.6% Between-group difference (90% CI) 4.8% (–2.6 to 12.3)	Physical performance (6-wk) ROM extension, flexion
Shim et al [48]; Korea; some concerns	Post-TKA and discharged home; mean age 68‐72 years; 75%‐82% women	2 Arms: Exercises using Microsoft Kinect, N=28 (27) Conventional rehabilitation, N=28 (27)	Home; 12 weeks	WOMAC (total) Baseline (SD): Intervention: 83.1 (13.0) Comparator: 81.1 (14.4) Diff Δ not 3-week: 1.9 12-week: –0.8 24-week: –2.8	NRS Baseline (SD): Intervention: 5.7 (2.1) Comparator: 5.5 (2.2) Diff Δ 3-week: 0.2 12-week: –0.7 24-week: 0	NR	Quality of life (3-mo) EQ-5D Physical performance (3, 12, and24 wk) 4 meter gait speed Berg balance scale Quadriceps strength Hamstring strength ROM

a RoB: risk of bias.

b Results for other eligible outcomes are found in Multimedia Appendix 11.

c VR: virtual reality.

d TKA: total knee arthroplasty.

e KOOS: Knee Injury and Osteoarthritis Outcome Score.

f Diff Δ: difference in change scores.

g Diff Δ calculated by review team, unable to standardize as no SD for change reported.

h VAS: Visual Analog Scale.

i Diff Δ not reported and cannot be calculated using provided results.

j NR: not reported.

k WOMAC: Western Ontario and McMaster Universities Osteoarthritis Index.

l ROM: range of motion.

m NRS: Numeric Rating Scale.

n IDR: interactive digital rehabilitation.

o THA: total hip arthroplasty.

p SF-36: 36-Item Short Form Health Survey.

q TUG: Timed Up and Go test.

r PT: physical therapy.

s standardized Diff Δ calculated by review team.

t Study reported *P* values for analyses comparing change scores between groups (from baseline to indicated time points).

u XR: extended reality.

v VERA: Virtual Exercise Rehabilitation Assistant.

#### VR Trials

##### VR-DAE (k=2)

Jin et al [37] (China, high RoB; N=66) evaluated immersive VR rowing beginning on postoperative day 2, with patients performing knee flexion exercises via immersive VR (30 min; 3 times/day) compared with passive knee flexion exercises. Nishitha et al [43] (India, high RoB; N=36) used 3D head-mounted VR with limb sensors for interactive games over 12 weeks. VR-DAE may result in improvements in pain-related functioning and pain intensity (low COE). At 1, 3, and 6 months, Jin et al [37] reported greater improvements in WOMAC total scores (6 mo Diff Δ −5.6), as did Nishitha et al [43] who reported similar improvements at 9 (Diff Δ −11.1) and 12 weeks (Diff Δ −14.3). For pain intensity, Jin et al [37] reported VAS scores on postoperative days 1‐7 showing slightly greater reductions in the VR group (Diff Δ −0.5 at day 7). Nishitha et al [43] showed a similar magnitude of effect at longer-term follow-up (12 wk Diff Δ −0.9). Neither VR-DAE study reported AEs. Knee ROM at 14 days showed small improvement favoring VR (Diff Δ 6.7). Both authors reported improvements for VR arms relative to comparators in ROM physical performance measures, eg Diff Δ 6.7 (favoring VR) at 2 weeks follow-up [37].

##### VR-Distraction (k=1)

Fuchs et al [33] (Israel, high RoB; N=55) evaluated passive VR distraction in which patients watched nature or music films via Oculus headset during continuous passive motion (CPM) therapy on postoperative days 1‐2. The comparator received CPM only. Modified KOOS scores improved in both groups at 6 months with minimal between-group difference (Diff Δ 1.1 [low COE]). Because standardized effect size could not be calculated, VAS pain intensity was reported only as medians (IQRs), which favored a greater decrease in scores with VR (very low COE). AEs were not reported. The passive nature of this intervention (distraction without exercise guidance) is mechanistically distinct from VR-DAE.

##### VR-Psychoeducation (k=1)

Mazurek et al [40] (Poland, some concerns RoB; N=68) evaluated VRTierOne (HTC Vive headset) delivering VR rehabilitation sessions with a psychoeducation focus over 4 weeks in patients aged ≥60 years following hip or knee arthroplasty. Knee-specific data were not disaggregated from the mixed hip/knee population. Pain-related functioning, measured by the Barthel Index, showed large improvement favoring VR (Diff Δ 21.2; *P*<.001) and pain intensity measured by VAS showed marked reduction favoring VR (Diff Δ −2.6; *P*<.001; moderate COE). AEs were not reported. Physical performance outcomes (eg, Tinetti balance and Rivermead Motor Assessment) each favored VR at 4 weeks. The large effect sizes and moderate COE for pain reduction are notable, though the mixed population and unclear intervention content limit generalizability. This was the only post-TKA VR study to achieve moderate certainty of evidence for any outcome.

### IDR

Compared with standard rehabilitation, IDR-DAE may result in better pain-related functioning at 3‐4 months (low COE) and probably results in better functioning at 6 months (moderate COE and 1 randomized controlled trial [RCT]). IDR may result in little to no difference in pain intensity at 2‐3 weeks (low COE), 3‐4 months (low COE), or 6 months (moderate COE), and the evidence for AEs remains very uncertain (Multimedia Appendix 7). Five trials evaluated IDR interventions for patients post-TKA [31,35,46-48]. Three used Microsoft Kinect-based systems [31,35,48] and 2 used other sensor technologies that monitored participant movements and provided digital feedback [46,47] (Table 4). Studies ranged from small to large (n=52‐306) and follow-up ranged from 2 weeks to 6 months. Four [31,35,47,48] were home-based, and one [46] was clinic-based. Four studies [31,35,47,48] reported on pain-related functioning, and 4 [35,46-48] reported on pain intensity; only one reported on AEs. One study [31] included patients who had undergone total arthroplasty of the knee or hip.

Four studies [31,35,47,48] evaluated pain-related functioning using WOMAC [31,48], Oxford Knee Score (OKS) [35], or KOOS [47]. At 3‐4 months, findings broadly favored IDR (low COE). Eichler et al [31] (Germany, high RoB; N=111) and Shim et al [48] (South Korea, some concerns RoB; N=56) both found WOMAC improvement in both groups at 3 months, with small reductions favoring IDR (eg, standardized Diff Δ −0.29 [48]). Likewise, Janhunen et al [35] (Finland, some concerns RoB; N=52) reported greater OKS improvement in the IDR group at 4 months (standardized Diff Δ 0.3). Prvu Bettger et al [47] (United States, some concerns RoB; N=306)—the largest study in the review and the only US-based trial—found greater KOOS improvement in the traditional PT group at 6 weeks (Diff Δ −1.8) but a difference favoring IDR at 12 weeks (Diff Δ 1.4). Notably, Prvu Bettger et al [47] was industry-funded (ReflexionHealth), and the primary outcome was total health care costs rather than pain or function. At 24 weeks, Shim et al [48] (moderate COE) reported continued WOMAC improvement favoring IDR (Diff Δ −2.8)—the longest follow-up and the highest certainty rating for any IDR outcome in the post-TKA analysis. Piqueras et al [46] (Spain, high RoB; N=181) also assessed participants using WOMAC scores. The authors, however, did not provide follow-up scores, although they stated there were “no significant differences” between groups. This study primarily compared delivery modality (remote vs face-to-face) rather than IDR content.

**Figure 4.**
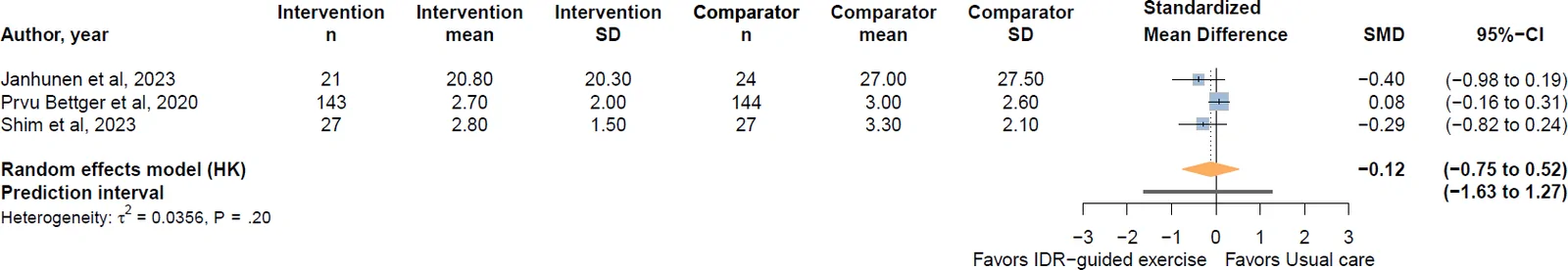
Forest plot of random-effects meta-analysis of interactive digital rehabilitation (IDR) versus usual care: Pain intensity at 12 weeks (k=3). IDR: interactive digital rehabilitation; SMD: standardized mean difference [35,47,48] (Multimedia Appendix 12).

Four trials assessed pain intensity using VAS or NRS. The evidence is very uncertain on the effect of IDR in pain intensity at 2‐3 weeks (very low COE; k=2) and at 3‐4 months (low COE; k=4). At 6 months (1 RCT), IDR probably resulted in little to no difference (moderate COE; k=1). Pooled analysis at 3‐4 months (k=3) showed no differences between groups (low COE) in reduction of pain intensity (SMD −0.12; 95% CI −0.75 to 0.52; PI −1.63 to 1.27; Figure 4; Multimedia Appendix 7) [35,47,48]. While not included in the pooled analysis due to intervention characteristics, Piqueras et al [46] showed greater VAS reduction in the control arm at 3 months (standardized Diff Δ 0.22; *P*=.28).

Only Prvu Bettger et al [47] reported on AEs, assessing falls during 12 weeks postdischarge; 19% (n=27) of the IDR group versus 15% (n=20) of the control group experienced one or more falls. Causality was not assessed; falls may reflect age and disease status rather than the intervention (very low COE). No other AE types were assessed in any of the 5 [31,35,46-48] IDR post-TKA studies. With respect to other outcomes, 2 studies assessed quality of life. Shim et al [48] reported EQ-5D scores at 3, 12, and 24 weeks with modest improvements in both groups and no clear between-group difference. Eichler et al [31] reported 36-item Short Form health survey (SF-36) physical component scores (PCS) improving comparably in both groups at 3 months (standardized Diff Δ −0.04), with no change in mental component scores (standardized Diff Δ −0.24). All 5 studies [31,35,46-48] evaluated physical performance using TUG, 6-minute walk, Short Performance Physical Battery (SPPB), ROM, gait speed, and/or strength measures. Results were heterogeneous; Janhunen et al [35] showed greater TUG improvement in the IDR group at 4 months (standardized Diff Δ −0.71; *P*=.04), whereas Piqueras et al [46] reported greater TUG improvement in the control group at 3 months (standardized Diff Δ 0.51, *P*=.02). Other physical performance measures showed either no differences or small inconsistent improvements between groups.

## Discussion

### Principal Findings

This systematic review evaluated the benefits and harms of XR interventions for chronic KOA pain and for symptomatic recovery after TKA, with findings stratified by level of immersion, clinical mechanism, and follow-up duration. We identified 21 eligible RCTs evaluating XR interventions for KOA pain or post-TKA rehabilitation. Despite a substantially expanded evidence base compared with previous reviews, the overall body of evidence remains limited by small study sizes, heterogeneity in intervention design and comparators, and lack of AE reporting in post-TKA. Most studies were rated as having high (k=9) or some concerns (k=11) for RoB, with only one study rated low. Findings should therefore be interpreted in light of the predominantly high or some concerns of RoB among the included studies, the low to very low GRADE certainty of evidence for most pooled outcomes, as well as the heterogeneity across interventions and follow-up durations. Pooled point estimates with 95% CIs reflect the average effect across the included studies, while prediction intervals describe the plausible range of true effects in similar future settings [60].

For adults with KOA, IDR interventions showed better pain-related functioning (k=5; low COE) and lower pain intensity (k=4; low COE) at 6‐8 weeks, compared with conventional therapy. The observed standardized effect sizes fall in the small-to-moderate range [56]. A consistent finding in both KOA and TKA studies was that both groups improved substantially over time, as assessed with established outcome measures (WOMAC, KOOS, and VAS), and met minimal clinically important differences (MCIDs) for these various measures (Multimedia Appendix 13 [61-65]). However, observed differences in change scores between groups (Diff Δ) typically were small and much less than MCID, though there should be caution in using MCID to interpret between-group differences [66]. Among the 10 IDR studies [28-30,32,34,36,38,41,42,44,49], the single low-RoB trial [29] did not report on WOMAC, and thus the pooled estimate of effects on pain-related functioning depended on data for studies with high or some concerns for RoB. VR-DAE for KOA (k=2) produced inconsistent results: short-term clinic-based benefits reported by Özlü et al [45] and null findings in a longer home-based trial reported by Lo et al [39] raise questions about the sustainability and generalizability of VR effects for KOA. Heterogeneity in intervention content (gaming vs guided strengthening), setting (clinic vs home), and duration (3 vs 12 weeks) limited comparability and synthesis.

Post-TKA evidence similarly indicates that IDR may result in better pain-related functioning at 3‐4 months (low COE), and this benefit probably persists at 6 months (moderate COE; 1 RCT)—a trajectory suggesting durable functional gains from IDR-guided rehabilitation. For pain intensity, the pooled meta-analysis at 3‐4 months showed little to no difference between groups (k=3). At 6 months, pain intensity probably showed little to no difference (moderate COE; 1 RCT).

Our results broadly align with other recent reviews undertaken to evaluate effects of XR technologies for KOA. Wei et al [18] evaluated immersive and nonimmersive VR technologies for patients with KOA and found improvements in pain and WOMAC total scores (moderate and low COE). Byra et al [19] evaluated rehabilitation using VR and exergames in patients with KOA and found “no conclusive evidence” that XR interventions were more beneficial than standard rehabilitation. Both reviews included studies evaluating a range of XR technologies encompassing both VR and nonfully immersive modalities, although only Wei et al [18] stratified outcomes by degree of immersion. Wei et al [18] also presented subgroup analyses for pain and WOMAC scores by duration of therapy; however, in contrast with our review, neither of the previous reviews stratified results by duration of follow-up, which is of particular importance when assessing effects of an intervention for a chronic pain condition [67]. Neither review evaluated AEs. Two recent reviews evaluated XR for post-TKA but neither stratified results by level of immersion. Su et al [20] and Peng et al [21] reported similar results for XR versus conventional rehabilitation on measures of pain and functioning (WOMAC). Pain scores improved in intervention arms at early time points, ≤2 weeks and ≤1 month, respectively, but neither review reported positive results at later time points. WOMAC scores showed improvement at 1 month in both reviews but disagreed at later time points (>1‐6 months) and showed considerable heterogeneity. Though a relatively small number of studies were included in Su et al [20] (k=14) and Peng et al [21] (k=8), only the latter commented on the quality of evidence (Physiotherapy Evidence Database [PEDro] scale for differences in pain (low-quality) and function (high-quality) scores. Neither review evaluated AEs. By stratifying findings by level of immersion (VR vs IDR), clinical mechanism, and follow-up duration, and by appraising certainty of evidence using GRADE and systematically evaluating AEs alongside efficacy, the present review extends and complements these prior syntheses and helps clarify which XR modalities and indications are best supported by the current evidence.

The lack of high-quality (ie, low RoB) studies evaluating XR interventions for KOA and post-TKA recovery is an important gap to address in order to improve care of these populations. XR interventions are being widely tested and deployed in a variety of other acute and chronic pain conditions, but insurance coverage for potentially beneficial interventions will be contingent on sufficient evidence to demonstrate benefit (and possibly cost-effectiveness) [68-71]. Given the rapid development, deployment of, and diminishing costs of XR hardware and software, there is also a critical need for rigorous evaluation of associated AEs relative to their benefits so we can safely deploy these devices in clinical settings.

### Limitations

Defining the characteristics of interventions by level of immersion (VR vs IDR) can be challenging, as existing frameworks for XR technologies sometimes differ in where boundaries are drawn [72]. Although most previous systematic reviews of XR interventions for pain did not stratify results by level of immersion, we sought to operationalize the distinction between full immersion (ie, VR) and nonimmersive digitally augmented experiences (ie, IDR) in order to provide greater clarity on benefits and harms attributable to each group. To do this, we relied on author descriptions of interventions, which were sometimes limited. To ensure that a broad range of clinically relevant XR interventions were evaluated, we included IDR interventions that many would consider minimally immersive (eg, Microsoft Kinect), which could potentially dilute the impact of more immersive XR interventions (that were not VR). Finally, we limited eligibility to English-language studies, but we did identify studies conducted in predominantly non–English speaking countries.

### Evidence Gaps and Recommendations for Future Research

The evidence on XR interventions for pain related to KOA or TKA is hampered by small sample sizes, short duration of follow-up, and lack of robust reporting on AEs. Methodological concerns were frequently encountered in the included studies, including most frequently missing outcome data and deviations from the intended interventions. Higher-quality studies addressing these concerns are needed to reliably compare the effects of XR to non-XR therapies.

Consistent with the early state of the science in an emerging field, most of the studies included in this report, along with 3-quarters of ongoing or recently completed trials on XR interventions for chronic pain, were small (n<100) [73]. Small sample sizes limit the effects of randomization on balancing baseline measures and unmeasured confounding [74]. As described above, we compared between-group change in outcome measures to avoid imbalances created by small sample size and imbalanced confounders, but between-group change is still subject to some amount of confounding. Future studies with sample sizes sufficient to effectively balance participants by measured and unmeasured confounders will facilitate rigorous assessment of the efficacy of IDR interventions for KOA and recovery post-TKA.

Included studies also were limited to short-term follow-up, with most (n=16) only reporting on outcomes at less than 6 months. This renders confidence in the effects of XR in the longer-term treatment of KOA, a chronic disease, uncertain. Further, assessment of whether XR interventions improve medium- and long-term outcomes post-TKA is important when considering the added cost, complexity, and potential for AEs entailed by XR. Longer trials are needed to adequately assess the effects of XR interventions on symptoms of KOA and recovery after TKA.

Lack of reporting of AEs is a critical gap to address in future research, particularly for VR interventions and for post-TKA populations, as AEs are an important component of the patient experience and often influence whether someone will start or continue an intervention [75]. AEs should be assessed systematically and reported for each arm and involve participant interviews with open-ended questions and/or checklists [76]. Accurate observation of rare but potentially serious AEs will require substantially larger studies but is an essential safety assessment, particularly for the TKA population performing intensive daily exercise programming early after surgery.

Finally, only 6 [33,37,39,40,43,45] of 21 included studies evaluated the effects of more immersive (VR) technologies. This is a critical evidence gap given the pace at which VR technologies are evolving and the high likelihood that they will be applied to areas of clinical need as the barriers to developing high-quality software to support them are lessened. Further, no study compared VR and IDR interventions to explore the potential for relative effects of higher levels of intervention immersion. These comparative effectiveness studies are needed given the increased complexity and cost of VR relative to IDR interventions.

### Conclusions

In patients with KOA, IDR interventions may result in small improvements in pain-related functioning and pain intensity at 6‐8 weeks compared with conventional therapy (low COE). Evidence for VR interventions in KOA is limited to 2 trials with inconsistent results. For post-TKA rehabilitation, IDR may result in better pain-related functioning at 3‐4 months (low COE), with benefits probably persisting at 6 months (moderate COE; 1 RCT). IDR may result in little to no difference in pain intensity at 2‐3 weeks and 3‐4 months (low COE) and probably results in little to no difference at 6 months (moderate COE; 1 RCT). VR-delivered psychoeducation showed promise for post-TKA pain reduction in a single trial (moderate COE) but requires replication. AE reporting was absent in the majority of studies but suggested acceptably low rates in those that did (high and low COE for KOA and post-TKA, respectively). Larger high-quality RCTs with longer follow-up, standardized outcome reporting, and systematic safety monitoring are needed before XR interventions can be recommended as standard-of-care adjuncts for recovery after both KOA and TKA. By organizing findings around level of immersion, clinical mechanism, and follow-up duration, as well as systematically appraising AEs alongside efficacy outcomes, this review provides clinicians, patients, and policymakers with an actionable framework for translating current evidence into future practice. The most defensible near-term clinical implication is that IDR-based exercise is reasonable to consider as an adjunct to conventional KOA care in real-world settings, while broader VR adoption and routine integration of XR into post-TKA rehabilitation should await additional high-quality evidence with longer follow-up periods and systematic safety monitoring.

## Supplementary material

10.2196/84753 Multimedia Appendix 1 Excluded references.

10.2196/84753 Multimedia Appendix 2 Eligibility criteria.

10.2196/84753 Multimedia Appendix 3 Search strategies.

10.2196/84753 Multimedia Appendix 4 Author-reported XR equipment and devices.

10.2196/84753 Multimedia Appendix 5 Grade Working Group grades of evidence.

10.2196/84753 Multimedia Appendix 6 Certainty of evidence tables for knee osteoarthritis studies.

10.2196/84753 Multimedia Appendix 7 Certainty of evidence tables for posttotal knee arthroplasty studies.

10.2196/84753 Multimedia Appendix 8 Detailed characteristics for included trials on knee osteoarthritis pain.

10.2196/84753 Multimedia Appendix 9 Detailed characteristics for included trials on total knee arthroplasty.

10.2196/84753 Multimedia Appendix 10 Detailed results for knee osteoarthritis studies.

10.2196/84753 Multimedia Appendix 11 Detailed results for included total knee arthroplasty studies.

10.2196/84753 Multimedia Appendix 12 Detailed Characteristics for TKA

10.2196/84753 Multimedia Appendix 13 Outcome measure minimal clinically important difference (MCID) reference table.

10.2196/84753 Checklist 1 PRISMA checklist.

## Abbreviations

AE: adverse event
COE: certainty of evidence
CPM: continuous passive motion
DAE: digitally augmented exercise
Diff Δ: difference in change scores
GDT: Guideline Development Tool
GRADE: Grading of Recommendations, Assessment, Development, and Evaluation
IDR: interactive digital rehabilitation
KOA: knee osteoarthritis
KOOS: Knee Injury and Osteoarthritis Outcome Score
MCID: minimal clinically important difference
NRS: Numeric Rating Scale
OKS: Oxford Knee Score
PCS: SF-36 physical component score
PEDro: Physiotherapy Evidence Database
PRISMA: Preferred Reporting Items for Systematic Reviews and Meta-Analyses
PRISMA-S: Preferred Reporting Items for Systematic Reviews and Meta-Analyses Literature Search Extension
PROSPERO: International Prospective Register of Systematic Reviews
PT: physical therapy
RCT: randomized controlled trial
RoB: risk of bias
ROM: range of motion
SF-36: 36-item Short Form health survey
SPPB: Short Performance Physical Battery
TENS: transcutaneous electrical nerve stimulation
TKA: total knee arthroplasty
TUG: Timed Up and Go test
VAS: Visual Analog Scale
VR: virtual reality
WHOQOL-BREF: World Health Organization Quality of Life Brief Version
WOMAC: Western Ontario and McMaster Universities Arthritis Index
XR: extended reality
