# Supplementary material for: Extended Reality Interventions for Osteoarthritis of the Knee and Recovery After Total Knee Arthroplasty: Systematic Review and Meta-Analyses
Source: J Med Internet Res. 2026 Sep 8;28:e84753. doi: 10.2196/84753 (PMC13555783; doi:10.2196/84753)
Supplement: Supplementary file 1 [file jmir-v28-e84753-s001.docx]

1. Virtual reality system for the treatment of chronic pain and stroke rehabilitation. Journal of Pain & Palliative Care Pharmacotherapy 2008;**22**(1):83-84 Online First. *Ineligible study design*

2. HELPING VETS DEAL WITH CHRONIC PAIN: Virtual reality program provides real results. InMotion 2021;**31**(2):16-17 Online First. *Ineligible study design*

3. Erratum: Effects of an 8-Week Virtual Reality Training Program on Pain, Fall Risk, and Quality of Life in lderly Women with Chronic Low Back Pain: Double-Blind Randomized Clinical Trial (Games for Health Journal (2022) 11:2 (85-92) DOI: 10.1089/g4h.2021.0175). Games for Health Journal 2022;**11**(4):275 Online First. *Ineligible study design*

4. Adaikkammai S, Singhal M, Smita E, Sreenivas S, Abhishek Appaji M. Virtual Reality in Rehabilitating Amputees Suffering from Phantom Limb Pain. 2019 11th International Conference on Communication Systems and Networks, COMSNETS 2019 2019:801-06 Online First. *Ineligible study design*

5. Aivaliotis VI, Dlamini V, Callahan M, Kelly C, Nguyen LAB. 697 VIRTUAL REALITY MINDFULNESS THERAPY VS. VIRTUAL REALITY DISTRACTIVE THERAPY IN CHRONIC ABDOMINAL PAIN. Gastroenterology 2020;**158**(6 Supplement 1):S-145 Online First. *Ineligible study design*

6. Akbulut A, Aşçi G, Tarakçi E, Aydin MA, Zaim AH. A Wearable Device for Virtual Cyber Therapy of Phantom Limb Pain. 2018 International Conference on Artificial Intelligence and Data Processing, IDAP 2018 2019 Online First. *Ineligible study design*

7. Akbulut A, Gungor F, Tarakci E, Cabuk A, Aydin MA. Immersive virtual reality games for rehabilitation of phantom limb pain. TIPTEKNO 2019 - Tip Teknolojileri Kongresi 2019 Online First. *Ineligible study design*

8. Alazba A, Al-Khalifa H, AlSobayel H. A proposed game for promoting physical activities among people with low back pain using virtual reality. ACM International Conference Proceeding Series 2018:141-44 Online First. *Ineligible study design*

9. Alazba A, Al-Khalifa H, AlSobayel H. RabbitRun: An Immersive Virtual Reality Game for Promoting Physical Activities Among People with Low Back Pain †. Technologies 2019;**7**(1) Online First. *Ineligible population*

10. Alnuman N, Jbara AA. Video Games and the Prevalence of Musculoskeletal Disorders in Young Adults. 2022 IEEE Zooming Innovation in Consumer Technologies Conference, ZINC 2022 2022:34-38 Online First. *Ineligible population*

11. Alphonso AL, Monson BT, Zeher MJ, et al. Use of a virtual integrated environment in prosthetic limb development and phantom limb pain. Annual Review of CyberTherapy and Telemedicine 2012;**10**:305-09 Online First. *Ineligible study design*

12. Alves CM, Rezende AR, Marques IA, Silva DC, Paiva TS, Naves ELM. Serious Games and Virtual Reality in the Treatment of Chronic Stroke: Both Sides Rehabilitation. IFMBE Proceedings 2022;**83**:239-44 Online First. *Ineligible population*

13. Ambron E, Buxbaum L, Kuchenbecker K, Miller A, Coslett B. Virtual reality treatment for phantom limb pain. Neurorehabilitation and Neural Repair 2019;**33**(12):1083 Online First. *Ineligible study design*

14. Ambron E, Buxbaum LJ, Miller A, Stoll H, Kuchenbecker KJ, Coslett HB. Virtual Reality Treatment Displaying the Missing Leg Improves Phantom Limb Pain: A Small Clinical Trial. Neurorehabilitation and neural repair 2021;**35**(12):1100-11 Online First. *Ineligible study design*

15. Amin AM. Effectiveness of mobile virtual reality as a means for pain distraction. 2016 Online First. *Ineligible population*

16. Anam M, Sizemore K, Mansour H, et al. (309) Virtual Reality Walking for Neuropathic Pain in Spinal Cord Injury: Preliminary Efficacy Findings. Journal of Pain 2019;**20**:S51-S51 Online First. *Ineligible study design*

17. Anwar N, Karimi H, Ahmad A, et al. Virtual Reality Training Using Nintendo Wii Games for Patients With Stroke: Randomized Controlled Trial. JMIR serious games 2022;**10**(2):e29830 Online First. *Ineligible population*

18. Arnoni JLB, Kleiner AFR, Lima CRG, De Campos AC, Rocha NACF. Nonimmersive Virtual Reality as Complementary Rehabilitation on Functional Mobility and Gait in Cerebral Palsy: A Randomized Controlled Clinical Trial. Games for Health Journal 2021;**10**(4):254-63 Online First. *Ineligible population*

19. Artifon M, Adachi L, Schestatsky P. Proceedings #39: Effects of tDCS alone and combined with virtual reality in clinical practice. Brain Stimulation 2019;**12**(2):e109-e10 Online First. *Not published in English*

20. Bahirat K, Annaswamy T, Prabhakaran B. Mr.MAPP: Mixed reality for MAnaging phantom pain. MM 2017 - Proceedings of the 2017 ACM Multimedia Conference 2017:1558-66 Online First. *Ineligible study design*

21. Bahirat K, Raval G, Chung YY, et al. Using Mr. MAPp for lower limb phantom pain management. MM 2019 - Proceedings of the 27th ACM International Conference on Multimedia 2019:1071-75 Online First. *Ineligible study design*

22. Baltaci G, Harput G, Haksever B, Ulusoy B, Ozer H. Comparison between Nintendo Wii Fit and conventional rehabilitation on functional performance outcomes after hamstring anterior cruciate ligament reconstruction: prospective, randomized, controlled, double-blind clinical trial. Knee surgery, sports traumatology, arthroscopy 2013;**21**:880-87 Online First. *No eligible outcomes provided*

23. Bani Mohammad E, Ahmad M. Virtual reality as a distraction technique for pain and anxiety among patients with breast cancer: A randomized control trial. Palliative & supportive care 2019;**17**(1):29-34 Online First. *Ineligible setting or follow-up*

24. Baqai A, Memon K, Memon AR, Shah SMZA. Interactive Physiotherapy: An Application Based on Virtual Reality and Bio-feedback. Wireless Personal Communications 2019;**106**(4):1719-41 Online First. *Ineligible study design*

25. Bartlett J, Fisher E, Liikkanen S, Turunen J, Skog M, Eccleston C. The Design and Development of an Embodied Semi-Autonomous Mentoring Intelligence (SAMI) for Use in Virtual Reality Interventions, Operationalized for the Self-Management of Chronic Pain. Frontiers in Virtual Reality 2022;**3** Online First. *Ineligible study design*

26. Basha MA, Aboelnour NH, Alsharidah AS, Kamel FH. Effect of exercise mode on physical function and quality of life in breast cancer-related lymphedema: a randomized trial. Supportive care in cancer : official journal of the Multinational Association of Supportive Care in Cancer 2022;**30**(3):2101-10 Online First. *Ineligible population*

27. Behar C, Lustick M, Foreman MH, Webb J, Engsberg JR. Personalized virtual reality for upper extremity rehabilitation: Moving from the clinic to a home exercise program. Journal of Intellectual Disability - Diagnosis and Treatment 2016;**4**(3):160-69 Online First. *Ineligible population*

28. Beltran-Alacreu H, Navarro-Fernandez G, Godia-Lledo D, et al. A Serious Game for Performing Task-Oriented Cervical Exercises Among Older Adult Patients With Chronic Neck Pain: Development, Suitability, and Crossover Pilot Study. JMIR serious games 2022;**10**(1):e31404 Online First. *Ineligible study design*

29. Benham S, Kang M, Grampurohit N. Immersive Virtual Reality for the Management of Pain in Community-Dwelling Older Adults. OTJR : occupation, participation and health 2019;**39**(2):90-96 Online First. *Ineligible study design*

30. Ber R, VanOosterhout S, Van Loo L, et al. TCT-261 Impact of Virtual Reality on Pre-Procedural Anxiety Prior to Heart Catheterization: The VR-THEIA Study. Journal of the American College of Cardiology 2022;**80**(12 Supplement):B103-B04 Online First. *Ineligible population*

31. Bogdanovych A, Chuan A. The Island of Pain: A Virtual Reality Experience for Patients with Chronic Pain. SIGGRAPH Asia 2020 XR, SA 2020 2020 Online First. *Ineligible study design*

32. Bolte B, e Lussanet M, Lappe M. Virtual reality system for the enhancement of mobility in patients with chronic back pain. Technology, rehabilitation and empowerment of people with special needs. 2015:47-59 Online First. *Ineligible study design*

33. Botella C, Garcia-Palacios A, Vizcaino Y, Herrero R, Banos RM, Belmonte MA. Virtual reality in the treatment of fibromyalgia: a pilot study. Cyberpsychology, behavior and social networking 2013;**16**(3):215-23 Online First. *Ineligible population*

34. Bottiroli S, Matamala-Gomez M, Allena M, et al. The virtual Enfacement Illusion on pain perception in patients suffering from chronic migraine: Preliminary data from a randomized controlled trial. Cephalalgia 2022;**42**(1 Supplement):47 Online First. *Ineligible study design*

35. Bratosin IA, Pavaloiu IB, Goga N, Luca AI. Virtual Reality Application for Pain Management: User Requirements. International Journal of Advanced Computer Science and Applications 2022;**13**(4):351-56 Online First. *Ineligible population*

36. Bratosin IA, Pavaloiu IB, Vasilateanu A, Gavajiuc D, Dragoi G, Goga N. Pain Relief using Virtual Reality. Proceedings of the 11th International Conference on Electronics, Computers and Artificial Intelligence, ECAI 2019 2019 Online First. *Ineligible study design*

37. Buttress S, Granat M, Barratt A, Roy B. The use of gamified virtual physiotherapy as an effective treatment for patients with shoulder problems. Physiotherapy (United Kingdom) 2019;**105**(Supplement 1):e162-e63 Online First. *Ineligible study design*

38. Cacau LdAP, Oliveira GU, Maynard LG, et al. The use of the virtual reality as intervention tool in the postoperative of cardiac surgery. Revista brasileira de cirurgia cardiovascular : orgao oficial da Sociedade Brasileira de Cirurgia Cardiovascular 2013;**28**(2):281-9 Online First. *Ineligible setting or follow-up*

39. Carrougher GJ, Hoffman HG, Nakamura D, et al. The effect of virtual reality on pain and range of motion in adults with burn injuries. Journal of burn care & research : official publication of the American Burn Association 2009;**30**(5):785-91 Online First. *Ineligible population*

40. Cawthorne D, March L, Parker D, Coolican M, Negus J. TKR-power-patient outcomes using wii enhanced rehabilitation after a total knee replacement. Physiotherapy (United Kingdom) 2015;**101**(SUPPL. 1):eS204-eS05 Online First. *Ineligible study design*

41. Chandler JM, Taylor JS, Portelli KI, et al. Small surgeries, big smiles: Reducing sedation through virtual reality. Journal of Laparoendoscopic and Advanced Surgical Techniques 2019;**29**(6):A64 Online First. *Ineligible population*

42. Chau B, Phelan I, Ta P, et al. Immersive Virtual Reality for Pain Relief in Upper Limb Complex Regional Pain Syndrome: A Pilot Study. Innovations in clinical neuroscience 2020;**17**(4-6):47-52 Online First. *Ineligible population*

43. Chavez T. (410) Therapeutic Virtual Reality in Pain Management. Journal of Pain 2019;**20**:S74-S74 Online First. *Ineligible study design*

44. Chen C-C. Multimedia virtualized environment for shoulder pain rehabilitation. Journal of physical therapy science 2016;**28**(4):1349-54 Online First. *No eligible outcomes provided*

45. Christensen SWM, Almsborg M H, Vain M TS, Vaegter HB. The Effect of Virtual Reality on Cold Pain Sensitivity in Patients with Fibromyalgia and Pain-Free Individuals: A Randomized Crossover Study. Games for health journal 2022 Online First. *No eligible outcomes provided*

46. Christiansen CL, Bade MJ, Davidson BS, Dayton MR, Stevens-Lapsley JE. Effects of weight-bearing biofeedback training on functional movement patterns following total knee arthroplasty: a randomized controlled trial. journal of orthopaedic & sports physical therapy 2015;**45**(9):647-55 Online First. *Ineligible intervention*

47. Chughtai M, Kelly JJ, Newman JM, et al. The Role of Virtual Rehabilitation in Total and Unicompartmental Knee Arthroplasty. The journal of knee surgery 2019;**32**(1):105-10 Online First. *Ineligible study design*

48. Clavelin G, Bouhier M, Tseng WJ, Gugenheimer J. Exploring the Perception of Pain in Virtual Reality through Perceptual Manipulations. Conference on Human Factors in Computing Systems - Proceedings 2023 Online First. *Ineligible population*

49. Cuneo A, Yang R, Wang K, et al. Utility of a Novel, Combined Biofeedback-Virtual Reality Tool as Add-on Treatment for Chronic Migraine. Neurology 2022;**98**(18 SUPPL) Online First. *Ineligible study design*

50. D. M P, L. A M, R. B W, L. J H. Effect of Virtual Reality Immersion on Phantom Limb Pain and Phantom Limb Sensation...45th Academy Annual Meeting and Scientific Symposium, March 6–9, 2019, Orlando, Florida. Journal of Prosthetics & Orthotics (JPO) 2019;**31**:6-6 Online First. *Ineligible study design*

51. Dagenais M, Brun C, Ohayon A, Mercier C. Virtual Reality in Fibromyalgia: Does Altering Visual Feedback Impact on Pain and Movement During Reaching? Frontiers in Virtual Reality 2021;**2** Online First. *Ineligible study design*

52. Dailey F, Tashjian VC, Mosadeghi S, et al. The clinical utility of virtual reality in inpatient pain management among patients with gastrointestinal disorders. American Journal of Gastroenterology 2016;**111**(Supplement 1):S464-S65 Online First. *Ineligible population*

53. Daste C, Foissac F, Abdoul H, Rannou F, Poiraudeau S, Nguyen C. Patient acceptable symptom state for patient-reported outcomes in 2 populations of patients with non-specific chronic low back pain: A secondary analysis of 2 randomized trials. Annals of Physical and Rehabilitation Medicine 2018 Online First. *Ineligible study design*

54. Demeter N, Josman N, Eisenberg E, Pud D. Who can benefit from virtual reality to reduce experimental pain? A crossover study in healthy subjects. European journal of pain (London, England) 2015;**19**(10):1467-75 Online First. *Ineligible population*

55. Depauw L, Bosteels A, Maes S, Sermeus L, Saldien V. Virtual reality hypnosis for postoperative pain after total knee arthroplasty. Acta Anaesthesiologica Belgica 2020;**71**:73-77 Online First. *Ineligible study design*

56. Deshpande AK, Bhatt I, Rojanaworarit C. Virtual reality for tinnitus management: a randomized controlled trial. International journal of audiology 2022;**61**(10):868-75 Online First. *Ineligible population*

57. Diaz-Orueta U, Alvarado S, Gutierrez D, Climent G, Banterla F. "Isla Calma", a Novel Virtual Reality Environment for Pain and Anxiety Distraction: Report on Usability, Acceptability, and Subjective Experience. Games for health journal 2012;**1**(5):353-61 Online First. *Ineligible study design*

58. DiMeola KA, Haynes J, Barone M, et al. A Pilot Investigation of Nonpharmacological Pain Management Intervention Groups in Methadone Maintenance Treatment. Journal of addiction medicine 2022;**16**(2):229-34 Online First. *No eligible outcomes provided*

59. Dingli A, Bondin L. Realtime adaptive virtual reality for pain reduction. IEEE Conference on Computatonal Intelligence and Games, CIG 2019;**2019-August** Online First. *Ineligible study design*

60. Donegan T, Ryan BE, Swidrak J, Sanchez-Vives MV. Immersive Virtual Reality for Clinical Pain: Considerations for Effective Therapy. Frontiers in Virtual Reality 2020;**1** Online First. *Ineligible study design*

61. Dulau E, Botha-Ravyse CR, Luimula M. Virtual reality for physical rehabilitation: A Pilot study How will virtual reality change physical therapy? 10th IEEE International Conference on Cognitive Infocommunications, CogInfoCom 2019 - Proceedings 2019:277-82 Online First. *Ineligible population*

62. e Carvalho MS, Carvalho LC, Alves RdS, et al. Analysis of the Muscular Activity, Peak Torque in the Lower Limbs, and Static Balance after Virtual Rehabilitation in Women with Fibromyalgia: A Randomized Controlled Study. Games for health journal 2021;**10**(3):190-97 Online First. *No eligible outcomes provided*

63. e Oliveira PF, Alves RdS, Iunes DH, et al. Effect of Exergaming on Muscle Strength, Pain, and Functionality of Shoulders in Cancer Patients. Games for health journal 2020;**9**(4):297-303 Online First. *Ineligible study design*

64. e Tommaso M, Ricci K, Laneve L, Savino N, Antonaci V, Livrea P. Virtual visual effect of hospital waiting room on pain modulation in healthy subjects and patients with chronic migraine. Pain research and treatment 2013;**2013**:515730 Online First. *Ineligible setting or follow-up*

65. e Villiers E, Stone T, Wang N-W, Sarangi V, Pelah A, Shenker N. Virtual Environment Rehabilitation for Patients with Motor Neglect Trial (VERMONT): A Single-Center Randomized Controlled Feasibility Trial. Brain sciences 2021;**11**(4) Online First. *Ineligible study design*

66. Ebrahimi N, Rojhani-Shirazi Z, Yoosefinejad AK, Nami M. The effects of virtual reality training on clinical indices and brain mapping of women with patellofemoral pain: a randomized clinical trial. BMC musculoskeletal disorders 2021;**22**(1):900 Online First. *Ineligible study design*

67. Elhafez HM, Elmageed SNA, Ali EA. Effect of Interactive Virtual Reality Device on Cervical Pain and Neck Function in Forward Head Posture. Egyptian Journal of Hospital Medicine 2022;**89**(1):6024-29 Online First. *Ineligible population*

68. Elor A, Teodorescu M, Kurniawan S. Project Star Catcher: A novel immersive virtual reality experience for upper limb rehabilitation. ACM Transactions on Accessible Computing 2018;**11**(4) Online First. *Ineligible population*

69. Erdoğan K, Ceylan R, Albayrak Gezer İ. The use of augmented reality in the teaching and training of basic exercises involved in the non-surgical treatment of neck pain. Virtual Reality 2023;**27**(1):481-92 Online First. *No eligible outcomes provided*

70. Faraj MM, Lipanski NM, Morales A, et al. A Virtual Reality Meditative Intervention Modulates Pain and the Pain Neuromatrix in Patients with Opioid Use Disorder. Pain medicine (Malden, Mass.) 2021;**22**(11):2739-53 Online First. *Ineligible population*

71. Fascio E, Vitale JA, Sirtori P, Peretti G, Banfi G, Mangiavini L. Early Virtual-Reality-Based Home Rehabilitation after Total Hip Arthroplasty: A Randomized Controlled Trial. Journal of Clinical Medicine 2022;**11**(7):1766 Online First. *Ineligible setting or follow-up*

72. Feintuch U, Tuchner M, Lorber-Haddad A, Meiner Z, Shiri S. VirHab - A virtual reality system for treatment of chronic pain and disability. 2009 Virtual Rehabilitation International Conference, VR 2009 2009:83-86 Online First. *Ineligible study design*

73. Ficklscherer A, Stapf J, Meissner KM, et al. Testing the feasibility and safety of the Nintendo Wii gaming console in orthopedic rehabilitation: a pilot randomized controlled study. Archives of Medical Science 2016;**12**(6):1273-78 Online First. *Ineligible population*

74. Fonseca M, Cardoso H, Ferreira N, Loureiro T, Gomes I, Quaresma C. VR4Neuropain: Interactive rehabilitation system. BIODEVICES 2019 - 12th International Conference on Biomedical Electronics and Devices, Proceedings; Part of 12th International Joint Conference on Biomedical Engineering Systems and Technologies, BIOSTEC 2019 2019:285-90 Online First. *Ineligible study design*

75. Ford S. Video game exercises 'can help treat chronic lower back pain'. Nursing Times 2018;**114**(9):148-48 Online First. *Ineligible study design*

76. Fowler CA, Ballistrea LM, Mazzone KE, et al. Virtual Reality as a Therapy Adjunct for Fear of Movement in Veterans With Chronic Pain: Single-Arm Feasibility Study. JMIR formative research 2019;**3**(4):e11266 Online First. *Ineligible study design*

77. Freedman S, Hoffman H. Interrupting traumatic memories: An emergency room virtual reality intervention for pain reduction and the prevention of PTSD. Journal of Cyber Therapy and Rehabilitation 2016;**9**(1):39 Online First. *Ineligible study design*

78. Fritz RC, Chaudhari AS, Boutin RD. Preoperative MRI of Articular Cartilage in the Knee: A Practical Approach. Journal of Knee Surgery 2020;**33**(11):1088-99 Online First. *Ineligible intervention*

79. Fung V, Ho A, Shaffer J, Chung E, Gomez M. Use of Nintendo Wii Fit TM in the rehabilitation of outpatients following total knee replacement: a preliminary randomised controlled trial. Physiotherapy 2012;**98**(3):183-8 Online First. *Ineligible setting or follow-up*

80. Fung V, Ho A, Shaffer J, Gomez M. The utilization of nintendo wii fit in the rehabilitation of outpatients following total knee replacements: Preliminary results of a randomized controlled trial. Archives of Physical Medicine and Rehabilitation 2010;**91**(10):e37 Online First. *Ineligible study design*

81. Fung V, Shaffer J, Chung E, Ho A, Gomez M. The utilization of nintendo wii fittm in the rehabilitation of outpatients following total knee replacements-a randomized controlled trial. Physiotherapy (United Kingdom) 2011;**97**(SUPPL. 1):eS419 Online First. *Ineligible population*

82. Gad R, Wilson M. Can we safely manage pain using virtual reality (VR)? Pain Management Nursing 2023;**24**(3):243-45 Online First. *Ineligible study design*

83. Gauthier LV, Kane C, Borstad A, et al. Video Game Rehabilitation for Outpatient Stroke (VIGoROUS): protocol for a multi-center comparative effectiveness trial of in-home gamified constraint-induced movement therapy for rehabilitation of chronic upper extremity hemiparesis. BMC neurology 2017;**17**(1):109 Online First. *Ineligible study design*

84. Gazerani P. Virtual reality for pain control - Virtual or real? European Neurological Review 2016;**12**(2) Online First. *Ineligible study design*

85. Geman O, Chiuchisan I, Vicoveanu D. Serious Games used in Rehabilitation of Patients with Osteoarthritis. 2021 9th E-Health and Bioengineering Conference, EHB 2021 2021 Online First. *Ineligible study design*

86. Giap B, Xavier M, Malekirad J, Giap HB. Virtual Reality Therapy Can Improve Fatigue and Cognitive Function after Radiation and Chemotherapy. International Journal of Radiation Oncology Biology Physics 2019;**105**(1 Supplement):E585 Online First. *Ineligible study design*

87. Glavare M, Stalnacke B-M, Hager CK, Lofgren M. Virtual Reality Exercises in an Interdisciplinary Rehabilitation Programme for Persons with Chronic Neck Pain: a Feasibility Study. Journal of rehabilitation medicine. Clinical communications 2021;**4**:1000067 Online First. *Ineligible study design*

88. Gray E, Erickson M, Bindler R, Eti DU, Wilson M. Experiencing COMFORT: Perceptions of Virtually-delivered Nonpharmacologic Therapies in Adults Prescribed Opioids for Chronic Pain. Pain Management Nursing 2023 Online First. *Ineligible study design*

89. Gray ML, Goldrich DY, McKee S, et al. Virtual Reality as Distraction Analgesia for Office-Based Procedures: A Randomized Crossover-Controlled Trial. Otolaryngology--head and neck surgery : official journal of American Academy of Otolaryngology-Head and Neck Surgery 2021;**164**(3):580-88 Online First. *Ineligible setting or follow-up*

90. Gromala D, Barnes SJ, Song M, et al. Immersive VR: A non-pharmacological analgesic for chronic pain? Conference on Human Factors in Computing Systems - Proceedings 2011:1171-76 Online First. *Ineligible study design*

91. Gromala D, Tong X, Choo A, Karamnejad M, Shaw CD. The virtual meditative walk: Virtual reality therapy for chronic pain management. Conference on Human Factors in Computing Systems - Proceedings 2015;**2015-April**:521-24 Online First. *Ineligible study design*

92. Groninger H, Stewart D, Fisher JM, Tefera E, Cowgill J, Mete M. Virtual reality for pain management in advanced heart failure: A randomized controlled study. Palliative medicine 2021;**35**(10):2008-16 Online First. *Ineligible population*

93. Groninger H, Stewart D, Wesley D, Cowgill J, Mete M. Virtual reality for management of cancer pain: Study rationale and design. Contemporary clinical trials communications 2022;**26**:100895 Online First. *Ineligible study design*

94. Gu Y, Yao Q, Xu Y, Zhang H, Wei P, Wang L. A Clinical Application Study of Mixed Reality Technology Assisted Lumbar Pedicle Screws Implantation. Medical science monitor : international medical journal of experimental and clinical research 2020;**26**:e924982 Online First. *Ineligible intervention*

95. Guarino D, La Paglia F, Daino M, et al. Chronic pain treatment through virtual reality. Annual Review of CyberTherapy and Telemedicine 2017;**15**:181-84 Online First. *Not published in English*

96. Guerra-Armas J, Flores-Cortes M, Pineda-Galan C, Luque-Suarez A, La Touche R. Role of Immersive Virtual Reality in Motor Behaviour Decision-Making in Chronic Pain Patients. Brain Sciences 2023;**13**(4) Online First. *Ineligible study design*

97. Gulsen CPTM, Soke FPTP, Eldemir KPTM, et al. Effect of fully immersive virtual reality treatment combined with exercise in fibromyalgia patients: a randomized controlled trial. Assistive technology : the official journal of RESNA 2022;**34**(3):256-63 Online First. *Ineligible population*

98. Gunduz AG, Soke F, Aydin Y, et al. An investigation of effects of immersive virtual reality combined with exercise training in fibromyalgia patients: A pilot study. Fizyoterapi Rehabilitasyon 2018;**29**(2):S12 Online First. *Ineligible study design*

99. Gutierrez-Maldonado J, Gutierrez-Martinez O, Cabas-Hoyos K. Interactive and passive virtual reality distraction: effects on presence and pain intensity. Studies in health technology and informatics 2011;**167**:69-73 Online First. *Ineligible population*

100. Hadamus A, Bialoszewski D, Blazkiewicz M, et al. Assessment of the Effectiveness of Rehabilitation after Total Knee Replacement Surgery Using Sample Entropy and Classical Measures of Body Balance. Entropy (Basel, Switzerland) 2021;**23**(2) Online First. *No eligible outcomes provided*

101. Hadamus A, Blazkiewicz M, Wydra KT, et al. Effectiveness of Early Rehabilitation with Exergaming in Virtual Reality on Gait in Patients after Total Knee Replacement. Journal of clinical medicine 2022;**11**(17) Online First. *No eligible outcomes provided*

102. Haffar A, Bryan S, Harwood M, Lonner JH. Patient Resilience Has Moderate Correlation With Functional Outcomes, but Not Satisfaction, After Primary Unilateral Total Knee Arthroplasty. Arthroplasty today 2021;**10**:123-27 Online First. *Ineligible intervention*

103. Haisley KR, Straw OJ, Muller DT, et al. Feasibility of implementing a virtual reality program as an adjuvant tool for peri-operative pain control; Results of a randomized controlled trial in minimally invasive foregut surgery. Complementary therapies in medicine 2020;**49**:102356 Online First. *Ineligible population*

104. Hammond A, O'Brien R, Woodbridge S, et al. Job retention vocational rehabilitation for employed people with inflammatory arthritis (WORK-IA): a feasibility randomized controlled trial. BMC musculoskeletal disorders 2017;**18**(1):315 Online First. *Ineligible intervention*

105. Hammond A, Prior Y, O'Brien R, Woodbridge S, Radford K. Work rehabilitation in inflammatory arthritis: A pilot randomised controlled trial. Annals of the Rheumatic Diseases 2014;**73**(SUPPL. 2) Online First. *Ineligible study design*

106. Hansford KJ, Baker DH, McKenzie KJ, Preston CEJ. Distinct Neural Signatures of Multimodal Resizing Illusions: Implications for Chronic Pain Treatment. bioRxiv 2023 Online First. *No eligible outcomes provided*

107. Hemphill S, Rodriguez S, Wang E, et al. Virtual Reality Augments Movement during Physical Therapy. American Journal of Physical Medicine and Rehabilitation 2022;**101**(3):229-36 Online First. *Ineligible population*

108. Hennessy R, Rumble D, Sprigner C, et al. (380) Preliminary Feasibility of a Graded, Locomotor-Enabled, Whole-Body Virtual Reality Intervention for Individuals with Chronic Low Back Pain. Journal of Pain 2019;**20**:S69-S69 Online First. *Ineligible study design*

109. Hennessy RW, Rumble D, Christian M, Brown DA, Trost Z. A Graded Exposure, Locomotion-Enabled Virtual Reality App During Walking and Reaching for Individuals With Chronic Low Back Pain: Cohort Gaming Design. JMIR serious games 2020;**8**(3):e17799 Online First. *Ineligible population*

110. Henriksen B, Nielsen R, Kraus M, Geng B. A virtual reality system for treatment of phantom limb pain using game training and tactile feedback. ACM International Conference Proceeding Series 2017 Online First. *Ineligible study design*

111. Henriksen B, Nielsen RN, Kraus M, Geng B. Comparison of movements in a virtual reality mirror box therapy for treatment of lower limb phantom pain. VISIGRAPP 2018 - Proceedings of the 13th International Joint Conference on Computer Vision, Imaging and Computer Graphics Theory and Applications 2018;**1**:167-74 Online First. *Ineligible study design*

112. Herrero R, Garcia-Palacios A, Castilla D, Molinari G, Botella C. Virtual reality for the induction of positive emotions in the treatment of fibromyalgia: a pilot study over acceptability, satisfaction, and the effect of virtual reality on mood. Cyberpsychology, behavior and social networking 2014;**17**(6):379-84 Online First. *Ineligible study design*

113. Hoffman HG, Fontenot MR, Garcia-Palacios A, et al. Adding tactile feedback increases avatar ownership and makes virtual reality more effective at reducing pain in a randomized crossover study. Scientific Reports 2023;**13**(1) Online First. *Ineligible population*

114. Holly J, Bridgewater C, Lambert AM. The use of virtual reality as a treatment medium for autonomic dysfunction across three diagnostic groups: Case studies. International Conference on Virtual Rehabilitation, ICVR 2017;**2017-June** Online First. *Ineligible study design*

115. Houdayer E, Alemanno F, Emedoli D, Locatelli M, Iannaccone S. Restoring "body image" in chronic pain: An innovative application of virtual reality. Neurorehabilitation and Neural Repair 2018;**32**(4-5):354 Online First. *Ineligible study design*

116. Hsiao PW, Kong LQ, Ye YL. Interactive game experience: a preliminary investigation of the impact of virtual reality on exercise rehabilitation behaviors. Interactive Learning Environments 2023 Online First. *Ineligible population*

117. Hsu JK, Thibodeau R, Wong SJ, Zukiwsky D, Cecile S, Walton DM. A "Wii" bit of fun: the effects of adding Nintendo Wii( R) Bowling to a standard exercise regimen for residents of long-term care with upper extremity dysfunction. Physiotherapy theory and practice 2011;**27**(3):185-93 Online First. *Ineligible population*

118. Hugli AS, Ernst MJ, Kool J, et al. Adherence to home exercises in non-specific low back pain. A randomised controlled pilot trial. Journal of bodywork and movement therapies 2015;**19**(1):177-85 Online First. *No eligible outcomes provided*

119. Hung ES-W, Chen S-C, Chang F-C, Shiao Y, Peng C-W, Lai C-H. Effects of Interactive Video Game-Based Exercise on Balance in Diabetic Patients with Peripheral Neuropathy: An Open-Level, Crossover Pilot Study. Evidence-based complementary and alternative medicine : eCAM 2019;**2019**:4540709 Online First. *No eligible outcomes provided*

120. Igna R, Stefan S, Onac I, Onac I, Ungur R-A, Szentagotai Tatar A. Mindfulness-based cognitive-behavior therapy (MCBT versus virtual reality (VR) enhanced CBT, versus treatment as usual for chronic back pain. A clinical trial. Journal of Evidence-Based Psychotherapies 2014;**14**(2):229-47 Online First. *Ineligible study design*

121. Inoue R, Sumitani M, Otake Y, et al. The effect of our physical training equipment presenting exercise instructions in the virtual reality space for chronic musculoskeletal pain. Pain Practice 2020;**20**(SUPPL 1):88 Online First. *Ineligible study design*

122. Jachak S, Phansopkar P. Effect of Oculus Guided Physical Therapy in adjunct to Conventional Therapy in Knee Osteoarthritis Patients. Journal of Medical Pharmaceutical and Allied Sciences 2022;**11**(4):5184-89 Online First. *Ineligible study design*

123. Janhunen M, Lopponen A, Walker S, et al. Movement characteristics during customized exergames after total knee replacement in older adults. Frontiers in sports and active living 2022;**4**:915210 Online First. *Ineligible population*

124. Jansen-Kosterink SM, Huis In 't Veld RMHA, Schonauer C, Kaufmann H, Hermens HJ, Vollenbroek-Hutten MMR. A Serious Exergame for Patients Suffering from Chronic Musculoskeletal Back and Neck Pain: A Pilot Study. Games for health journal 2013;**2**(5):299-307 Online First. *Ineligible setting or follow-up*

125. Jin W, Choo A, Gromala D, Shaw C, Squire P. A Virtual Reality Game for Chronic Pain Management: A Randomized, Controlled Clinical Study. Studies in health technology and informatics 2016;**220**:154-60 Online First. *Ineligible setting or follow-up*

126. Johnson L, Bird M-L, Muthalib M, Teo W-P. Innovative STRoke Interactive Virtual thErapy (STRIVE) online platform for community-dwelling stroke survivors: a randomised controlled trial protocol. BMJ open 2018;**8**(1):e018388 Online First. *Ineligible study design*

127. Jones T, Moore T, Rose H, Choo J. (512) The impact of virtual reality on chronic pain. Journal of Pain 2016;**17**:S102-S03 Online First. *Ineligible study design*

128. Joo SY, Cho YS, Lee SY, Seok H, Seo CH. Effects of Virtual Reality-Based Rehabilitation on Burned Hands: A Prospective, Randomized, Single-Blind Study. Journal of clinical medicine 2020;**9**(3) Online First. *Ineligible study design*

129. Junior VADS, Santos MdS, Ribeiro NMdS, Maldonado IL. Combining Proprioceptive Neuromuscular Facilitation and Virtual Reality for Improving Sensorimotor Function in Stroke Survivors: A Randomized Clinical Trial. Journal of central nervous system disease 2019;**11**:1179573519863826 Online First. *Ineligible population*

130. Kamdar M, Centi AJ, Agboola S, et al. A randomized controlled trial of a novel artificial intelligence-based smartphone application to optimize the management of cancer-related pain. Journal of Clinical Oncology 2019;**37**(Supplement 15) Online First. *Ineligible study design*

131. Kapsigay B, Sari Z, Kavlak B, Aras I, Tanhan A. HPR effects of virtual rehabilitation on shoulder periarthritis. Annals of the Rheumatic Diseases 2017;**76**(Supplement 2):1496 Online First. *Ineligible study design*

132. Karakoc ZB, Kuru Colak T, Kapsigay B, Sari Z, Polat MG. HPR the effcet of adding Wii Fit games to traditional rehabilitation on pain, quality of life and functionality after ACL reconstruction. Annals of the Rheumatic Diseases 2017;**76**(Supplement 2):1537 Online First. *Ineligible study design*

133. Kazis LE, Nethercot VA, Ren XS, Lee A, Selim A, Miller DR. Medication effectiveness studies in the United States Veterans Administration health care system: A model for large integrated delivery systems. Drug Development Research 2006;**67**(3):217-26 Online First. *Ineligible intervention*

134. Keefe FJ, Huling DA, Coggins MJ, et al. Virtual reality for persistent pain: a new direction for behavioral pain management. Pain 2012;**153**(11):2163-66 Online First. *Ineligible study design*

135. Kilbride C, Scott D, Butcher T, et al. Rehabilitation via home based gaming exercise for the upper-limb post stroke (RHOMBUS): results of an intervention feasibility trial. Physiotherapy (United Kingdom) 2020;**107**(Supplement 1):e25 Online First. *Ineligible population*

136. Kilbride C, Scott DJM, Butcher T, et al. Safety, feasibility, acceptability and preliminary effects of the Neurofenix platform for Rehabilitation via HOMe Based gaming exercise for the Upper-limb post Stroke (RHOMBUS): results of a feasibility intervention study. BMJ open 2022;**12**(2):e052555 Online First. *Ineligible study design*

137. Kim S-K, HwangBo G. The effects of horse-riding simulator exercise on balance in elderly with knee osteoarthritis. Journal of physical therapy science 2017;**29**(3):387-89 Online First. *No eligible outcomes provided*

138. Kim S-K, Kim S-G, HwangBo G. The effect of horse-riding simulator exercise on the gait, muscle strength and muscle activation in elderly people with knee osteoarthritis. Journal of physical therapy science 2017;**29**(4):693-96 Online First. *Ineligible study design*

139. Kim TY, Lee JH, Oh S, Kim SM, Yoon BC. Effect of simulated horseback-riding and sling exercise for patients with low back pain. Archives of Physical Medicine and Rehabilitation 2017;**98**(12):e175-e76 Online First. *Ineligible study design*

140. King A, Leggiero N, Acquaye A, et al. Use of virtual reality environments to improve self-reported distress, mood disturbance and overall symptom burden using correlative stress biomarkers in primary brain tumor (PBT) patients. Neuro-Oncology 2019;**21**(Supplement 6):vi219 Online First. *Ineligible study design*

141. Kiper P, Przysiezna E, Cieslik B, et al. Effects of Immersive Virtual Therapy as a Method Supporting Recovery of Depressive Symptoms in Post-Stroke Rehabilitation: Randomized Controlled Trial. Clinical interventions in aging 2022;**17**:1673-85 Online First. *Ineligible population*

142. Koo KI, Park DK, Youm YS, Cho SD, Hwang CH. Enhanced Reality Showing Long-Lasting Analgesia after Total Knee Arthroplasty: Prospective, Randomized Clinical Trial. Scientific Reports 2018;**8**(1) Online First. *Ineligible setting or follow-up*

143. Kragting M, Schuiling SF, Voogt L, Pool-Goudzwaard AL, Coppieters MW. Using visual feedback manipulation in virtual reality to influence pain-free range of motion in people with non-specific neck pain. Pain practice : the official journal of World Institute of Pain 2020 Online First. *Ineligible study design*

144. Krause K, Guertler D, Moehring A, et al. Association between Alcohol Consumption and Health-Related Quality of Life among Hospital and Ambulatory Care Patients with Past Year Depressive Symptoms. International journal of environmental research and public health 2022;**19**(22) Online First. *Ineligible intervention*

145. Krogg W, Golovyan D, Abshire L, et al. Development of a virtual reality environment to provide cognitive stimulation: A novel tool to reduce pain, anxiety, and delirium in critically ill patients. American Journal of Respiratory and Critical Care Medicine 2018;**197**(MeetingAbstracts) Online First. *Ineligible study design*

146. Kumru H, Soler D, Vidal J, et al. The effects of transcranial direct current stimulation with visual illusion in neuropathic pain due to spinal cord injury: an evoked potentials and quantitative thermal testing study. European journal of pain (London, England) 2013;**17**(1):55-66 Online First. *Ineligible study design*

147. Lehrl S, Gusinde J, Schulz-Drost S, et al. Advancement of physical process by mental activation: a prospective controlled study. Journal of Rehabilitation Research & Development 2012;**49**(8) Online First. *Ineligible intervention*

148. Lew D, Bancila L, Wang K, et al. Virtual Reality Can Reduce Pain and Opioid Use in Sphincter of Oddi Dysfunction (SOD) Type III: A Prospective Pilot Study. American Journal of Gastroenterology 2020;**115**(SUPPL):S39-S40 Online First. *Ineligible study design*

149. Li L. Effect of Remote Control Augmented Reality Multimedia Technology for Postoperative Rehabilitation of Knee Joint Injury. Computational and mathematical methods in medicine 2022;**2022**:9320063 Online First. *Ineligible setting or follow-up*

150. Lier EJ, Oosterman JM, Assmann R, e Vries M, van Goor H. The effect of Virtual Reality on evoked potentials following painful electrical stimuli and subjective pain. Scientific reports 2020;**10**(1):9067 Online First. *Ineligible population*

151. Liu K, Madrigal E, Chung JS, et al. Preliminary Study of Virtual-reality-guided Meditation for Veterans with Stress and Chronic Pain. Alternative therapies in health and medicine 2021 Online First. *Ineligible study design*

152. Llorens R, Noe E, Colomer C, Alcaniz M. Effectiveness, usability, and cost-benefit of a virtual reality-based telerehabilitation program for balance recovery after stroke: a randomized controlled trial. Archives of physical medicine and rehabilitation 2015;**96**(3):418-25.e2 Online First. *Ineligible population*

153. Luque-Moreno C, Cano-Bravo F, Kiper P, et al. Reinforced Feedback in Virtual Environment for Plantar Flexor Poststroke Spasticity Reduction and Gait Function Improvement. BioMed research international 2019;**2019**:6295263 Online First. *Ineligible population*

154. MacIntyre E, Sigerseth M, Larsen TF, et al. Get your head in the game: a replicated single-case-experimental-design evaluating the effect of a novel virtual reality intervention in people with chronic low back pain. The journal of pain 2023 Online First. *Ineligible study design*

155. Malik M, Shaw M, Palmeri M, Charvet L. Virtual reality as an intervention for chronic pain in multiple sclerosis. Neurology 2021;**96**(15 SUPPL 1) Online First. *Ineligible study design*

156. Manlapaz DG, Escuadra CT, Regino JM, et al. Effectiveness of exergaming, Tai Chi, and physical therapy in improving dynamic balance and enjoyment of older adults with knee osteoarthritis. Osteoarthritis and Cartilage 2020;**28**(Supplement 1):S166-S67 Online First. *Ineligible study design*

157. Marc A, Henry F, Duault E. The virtual reality headset, an alternative to pain and anxiety. Oxymag 2022;**35**(183):21-23 Online First. *Ineligible study design*

158. Marcuzzi A, Bach K, Nordstoga AL, et al. Individually tailored self-management app-based intervention (selfBACK) versus a self-management web-based intervention (e-Help) or usual care in people with low back and neck pain referred to secondary care: protocol for a multiarm randomised clinical trial. BMJ open 2021;**11**(9):e047921 Online First. *Ineligible study design*

159. Marsh J, Pettifer S, Richardson C, Kulkarni J. Experiences of treating phantom limb pain using immersive virtual reality. ACM SIGGRAPH 2019 Talks, SIGGRAPH 2019 2019 Online First. *Ineligible study design*

160. Marshall AN, Hertel J, Hart JM, Russell S, Saliba SA. Visual Biofeedback and Changes in Lower Extremity Kinematics in Individuals With Medial Knee Displacement. Journal of athletic training 2020;**55**(3):255-64 Online First. *Ineligible population*

161. Martínez D, Zavala J. Functional results when adding Microsoft Kinect® in the physiotherapeutic rehabilitation of patients with total knee arthroplasty. Fisioterapia 2020;**42**(6):295-300 Online First. *Ineligible study design*

162. Matamala-Gomez M, Diaz Gonzalez AM, Slater M, Sanchez-Vives MV. Decreasing Pain Ratings in Chronic Arm Pain Through Changing a Virtual Body: Different Strategies for Different Pain Types. The journal of pain 2019;**20**(6):685-97 Online First. *Ineligible study design*

163. Matheve T, Bogaerts K, Timmermans A. Virtual reality distraction induces hypoalgesia in patients with chronic low back pain: a randomized controlled trial. Journal of neuroengineering and rehabilitation 2020;**17**(1):55 Online First. *Ineligible setting or follow-up*

164. Matsangidou M, P. Kassianos A, Papaioannou D, et al. Virtual Painkillers: Designing Accessible Virtual Reality Experiences for Helping Cancer Patients Manage Pain at Home. Conference on Human Factors in Computing Systems - Proceedings 2022 Online First. *No eligible outcomes provided*

165. Mbada C, Oni A, Makinde M, Fasuyi F, Adegbemigun O, Awotidebe T. Does Virtual Reality Type Matter on Clinical Outcomes of Patients with Long-term Non-Specific Low-Back Pain? Archives of Physical Medicine and Rehabilitation 2019;**100**(12):e174 Online First. *Ineligible study design*

166. McDonald DD, Gifford T, Walsh S. Effect of a Virtual Pain Coach on Older Adults' Pain Communication: A Pilot Study. Pain Management Nursing 2011;**12**(1):50-56 Online First. *Ineligible intervention*

167. McFarland M, Zelaya N, Hossain G, Hicks D, McLauchlan L. Pain Mitigation through Virtual Reality Applications. 2019 22nd IEEE International Symposium on Measurement and Control in Robotics: Robotics for the Benefit of Humanity, ISMCR 2019 2019 Online First. *Ineligible study design*

168. Meinke A, Peters R, Knols RH, Swanenburg J, Karlen W. Feedback on Trunk Movements From an Electronic Game to Improve Postural Balance in People With Nonspecific Low Back Pain: Pilot Randomized Controlled Trial. JMIR serious games 2022;**10**(2):e31685 Online First. *Ineligible population*

169. Menek B, Tarakci D, Tarakci E, Menek MY. Investigation on the Efficiency of the Closed Kinetic Chain and Video-Based Game Exercise Programs in the Rotator Cuff Rupture: A Randomized Trial. Games for health journal 2022;**11**(5):298-306 Online First. *Ineligible setting or follow-up*

170. Mercier C, Roosink M, McFadyen BJ, Hebert LJ, Jackson PL, Bouyer LJ. Assessing the perception of movements in subjects with chronic pain using altered virtual feedback. Annals of Physical and Rehabilitation Medicine 2015;**58**(SUPPL. 1):e94 Online First. *Ineligible study design*

171. Merliot-Gailhoustet L, Raimbert C, Garnier O, et al. Discomfort improvement for critically ill patients using electronic relaxation devices: results of the cross-over randomized controlled trial E-CHOISIR (Electronic-CHOIce of a System for Intensive care Relaxation). Critical care (London, England) 2022;**26**(1):263 Online First. *Ineligible population*

172. Merlot B, Dispersyn G, Husson Z, et al. Pain Reduction With an Immersive Digital Therapeutic Tool in Women Living With Endometriosis-Related Pelvic Pain: Randomized Controlled Trial. Journal of medical Internet research 2022;**24**(9):e39531 Online First. *Ineligible setting or follow-up*

173. Mihajlovic Z, Popovic S, Brkic K, Cosic K. A system for head-neck rehabilitation exercises based on serious gaming and virtual reality. Multimedia Tools and Applications 2018;**77**(15):19113-37 Online First. *Ineligible study design*

174. Morkisch N, Jettkowski K, Dos Santos LF, Dohle C. Modelling of therapeutic action during mirror therapy. Current Directions in Biomedical Engineering 2017;**3**(1):45-48 Online First. *Ineligible study design*

175. Morris NA, Wang Y, Felix RB, et al. Adjunctive virtual reality pain relief after traumatic injury: a proof-of-concept within-person randomized trial. Pain 2023 Online First. *Ineligible setting or follow-up*

176. Mortensen J, Kristensen LQ, Brooks EP, Brooks AL. Women with fibromyalgia's experience with three motion-controlled video game consoles and indicators of symptom severity and performance of activities of daily living. Disability and rehabilitation. Assistive technology 2015;**10**(1):61-6 Online First. *Ineligible study design*

177. Mouraux D, Brassinne E, Sobczak S, et al. 3D augmented reality mirror visual feedback therapy applied to the treatment of persistent, unilateral upper extremity neuropathic pain: a preliminary study. The Journal of manual & manipulative therapy 2017;**25**(3):137-43 Online First. *Ineligible study design*

178. Mukherjee M, Bedekar N, Sancheti PK, Shyam A. Immediate and short-term effect of virtual reality training on pain, range of motion, and kinesiophobia in patients with cervical spondylosis. Indian Journal of Physical Therapy and Research 2020;**2**(1):55 Online First. *Ineligible population*

179. Najafi B, Grewal G, Sayeed R, et al. Early detection of peripheral neuropathy using virtual obstacle crossing. Diabetes 2012;**61**(SUPPL. 1):A162 Online First. *Ineligible intervention*

180. Nakad L, Rakel B. (271) Attitudes of Older Adults with Chronic Musculoskeletal Pain towards Immersive Virtual Reality. Journal of Pain 2019;**20**:S42-S42 Online First. *No eligible outcomes provided*

181. Nambi G, Abdelbasset WK, Alqahatani BA. Radiological (Magnetic Resonance Image and Ultrasound) and biochemical effects of virtual reality training on balance training in football players with chronic low back pain: A randomized controlled study. Journal of back and musculoskeletal rehabilitation 2021;**34**(2):269-77 Online First. *No eligible outcomes provided*

182. Nguyen LAB, Adler EP, Rodriguez S, et al. VIRTUAL REALITY MINDFULNESS THERAPY REDUCES PAIN AND ANXIETY IN PATIENTS WITH CHRONIC ABDOMINAL PAIN. Gastroenterology 2019;**156**(6 Supplement 1):S-1062 Online First. *Ineligible study design*

183. Ortiz-Catalan M, Gumundsdottir RA, Kristoffersen MB, et al. Phantom motor execution facilitated by machine learning and augmented reality as treatment for phantom limb pain: a single group, clinical trial in patients with chronic intractable phantom limb pain. Lancet (London, England) 2016;**388**(10062):2885-94 Online First. *Ineligible study design*

184. Ozkul C, Kilinc M, Yildirim SA, Topcuoglu EY, Akyuz M. Effects of visual illusion and transcutaneous electrical nerve stimulation on neuropathic pain in patients with spinal cord injury: A randomised controlled cross-over trial. Journal of back and musculoskeletal rehabilitation 2015;**28**(4):709-19 Online First. *Ineligible intervention*

185. Palmeri M, Shaw M, Dobbs B, et al. Abstract #144: Virtual Reality (VR) to Improve Quality of Life in Patients Diagnosed with Neurological Disorders. Brain Stimulation 2019;**12**(2):e49 Online First. *Ineligible population*

186. Pamment J, Aspell JE. Putting pain out of mind with an 'out of body' illusion. European journal of pain (London, England) 2017;**21**(2):334-42 Online First. *Ineligible study design*

187. Park H-Y, Nam KE, Lim J-Y, Yeo SM, In Lee J, Hwang JH. Real-time interactive digital healthcare system for post-operative breast cancer patients: study protocol for a randomized controlled trial. Trials 2021;**22**(1):549 Online First. *Ineligible study design*

188. Park H-Y, Nam KE, Lim J-Y, Yeo SM, Lee JI, Hwang JH. Real-Time Interactive Digital Health Care System for Postoperative Breast Cancer Patients: A Randomized Controlled Trial. Telemedicine journal and e-health : the official journal of the American Telemedicine Association 2022 Online First. *Ineligible population*

189. Park J-H, Lee S-H, Ko D-S. The Effects of the Nintendo Wii Exercise Program on Chronic Work-related Low Back Pain in Industrial Workers. Journal of physical therapy science 2013;**25**(8):985-8 Online First. *Ineligible study design*

190. Patel P, Ivanov D, Bhatt S, et al. Low-Cost Virtual Reality Headsets Reduce Perceived Pain in Healthy Adults: A Multicenter Randomized Crossover Trial. Games for health journal 2020;**9**(2):129-36 Online First. *Ineligible population*

191. Patricio NA, Vidal DG, Pinto EB, Sa KN, Baptista AF. Effectiveness of virtual reality games for falls, postural oscillations, pain and quality of life of individual HAM/TSP: a randomized, controlled, clinical trial. Journal of neurovirology 2020;**26**(5):676-86 Online First. *Ineligible study design*

192. Patterson DR, Hoffman HG, Palacios AG, Jensen MJ. Analgesic effects of posthypnotic suggestions and virtual reality distraction on thermal pain. Journal of abnormal psychology 2006;**115**(4):834-41 Online First. *Ineligible population*

193. Patterson DR, Soltani M, Teeley A, Morse D, Wiechman SA, Gibran NS. Hypnosis delivered through immersive virtual reality for wound care: A randomized, controlled study. Journal of Burn Care and Research 2012;**33**(2 SUPPL. 1):S70 Online First. *Ineligible study design*

194. Payne O, Smith V, Rolnik DL, et al. Virtual reality and its use in post-operative pain following laparoscopy: a feasibility study. Scientific reports 2022;**12**(1):13137 Online First. *Ineligible study design*

195. Pekyavas NO, Ergun N. Effects of different exercise and kinesiotaping application on pain, flexibility, strength and range of motion in patients with subacromial impingement syndrome. Fizyoterapi Rehabilitasyon 2014;**25**(1 SUPPL. 1):S31 Online First. *Ineligible study design*

196. Pekyavas NO, Ergun N. Comparison of virtual reality exergaming and home exercise programs in patients with subacromial impingement syndrome and scapular dyskinesis: Short term effect. Acta orthopaedica et traumatologica turcica 2017;**51**(3):238-42 Online First. *Ineligible population*

197. Perales FJ, Riera L, Ramis S, Guerrero A. Evaluation of a VR system for Pain Management using binaural acoustic stimulation. Multimedia Tools and Applications 2019;**78**(23):32869-90 Online First. *Ineligible study design*

198. Perez-Marcos D, Chevalley O, Schmidlin T, et al. Increasing upper limb training intensity in chronic stroke using embodied virtual reality: a pilot study. Journal of neuroengineering and rehabilitation 2017;**14**(1):119 Online First. *Ineligible study design*

199. Perna-Forrest L, Minton O. THE POTENTIAL FOR VIRTUAL REALITY THERAPY IN PALLIATIVE CARE - PRELIMINARY FINDINGS. BMJ Supportive and Palliative Care 2019;**9**(Supplement 1):A63 Online First. *Ineligible study design*

200. Perry BN, Armiger RS, Wolde M, et al. Clinical Trial of the Virtual Integration Environment to Treat Phantom Limb Pain With Upper Extremity Amputation. Frontiers in neurology 2018;**9**:770 Online First. *Ineligible study design*

201. Perry BN, Mercier C, Pettifer SR, Cole J, Tsao JW. Virtual reality therapies for phantom limb pain. 2014;**18**(7):897-99 Online First. *Ineligible study design*

202. Peterson BN, Hitching R, Howard L, et al. Immersive Virtual Reality: A Safe, Scalable, Non-opioid Analgesic for Military and Veteran Patients. Frontiers in Virtual Reality 2021;**2** Online First. *Ineligible study design*

203. Pettifer S, Howard T, Blundell B, Edwards D, Lieberman I. An immersive virtual environment for Phantom Limb Pain rehabilitation. GRAPP 2012 IVAPP 2012 - Proceedings of the International Conference on Computer Graphics Theory and Applications and International Conference on Information Visualization Theory and Applications 2012:426-33 Online First. *Ineligible study design*

204. Phelan I. How Virtual Reality Can Reduce Pain and Aid Rehabilitation. ITNOW 2022;**64**(3):22-23 Online First. *Ineligible study design*

205. Piccoli A, Samuels G, Mirakaj A, et al. Immersive Virtual Reality for individuals with spinal cord injuries. 2017 IEEE Long Island Systems, Applications and Technology Conference, LISAT 2017 2017 Online First. *Ineligible population*

206. Polat M, Kahveci A, Gunendi Z, Karatas GK. The effect of virtual reality exercises on pain, functionality, cardiopulmonary capacity and quality of life in fibromyalgia syndrome: A randomized, single-blind, controlled study. Annals of the Rheumatic Diseases 2019;**78**(Supplement 2):526 Online First. *Ineligible study design*

207. Popert S, Magar K. EXPANDING THE REACH OF VIRTUAL REALITY FOR PALLIATIVE PATIENTS. BMJ Supportive and Palliative Care 2021;**11**(Supplement 2):A59 Online First. *Ineligible study design*

208. Pournajaf S, Goffredo M, Pellicciari L, et al. Effect of balance training using virtual reality-based serious games in individuals with total knee replacement: A randomized controlled trial. Annals of physical and rehabilitation medicine 2022;**65**(6):101609 Online First. *Ineligible setting or follow-up*

209. Powell W, Stevens B, Simmonds M. Annual review of cybertherapy and telemedicine 2009. Treadmill interface for virtual reality vs. overground walking: a comparison of gait in individuals with and without pain. Studies in Health Technology & Informatics 2009;**144**:198-203 Online First. *Ineligible intervention*

210. Pozeg P, Palluel E, Ronchi R, et al. Virtual reality improves embodiment and neuropathic pain caused by spinal cord injury. Neurology 2017;**89**(18):1894-903 Online First. *No eligible outcomes provided*

211. Prabhu VG, Stanley LM, Linder C, Morgan R. Analyzing the Efficacy of a Restorative Virtual Reality Environment using HRV Biofeedback for Pain and Anxiety Management. Proceedings of the 2020 IEEE International Conference on Human-Machine Systems, ICHMS 2020 2020 Online First. *Ineligible population*

212. Prahm C, Bressler M, Eckstein K, Kuzuoka H, Daigeler A, Kolbenschlag J. Developing a wearable Augmented Reality for treating phantom limb pain using the Microsoft Hololens 2. ACM International Conference Proceeding Series 2022:309-12 Online First. *Ineligible study design*

213. Preatoni G, Bracher NM, Raspopovic S. Towards a future VR-TENS multimodal platform to treat neuropathic pain. International IEEE/EMBS Conference on Neural Engineering, NER 2021;**2021-May**:1105-08 Online First. *Ineligible population*

214. Preston C, Gilpin HR, Newport R. An exploratory investigation into the longevity of pain reduction following multisensory illusions designed to alter body perception. Musculoskeletal science & practice 2020;**45**:102080 Online First. *Ineligible intervention*

215. Putrino D, Tabacof L, Breyman E, et al. Pain Reduction after Short Exposure to Virtual Reality Environments in People with Spinal Cord Injury. International journal of environmental research and public health 2021;**18**(17) Online First. *Ineligible study design*

216. Rahbar M, Salekzamani Y, Jahanjou F, Eslamian F, Niroumand A, Dolatkhah N. Effect of hippotherapy simulator on pain, disability and range of motion of the spinal column in subjects with mechanical low back pain: A randomized single-blind clinical trial. Journal of back and musculoskeletal rehabilitation 2018;**31**(6):1183-92 Online First. *Ineligible population*

217. Ramsayer B, Honold M, Beck K, Kraus M, Kramer M, Dehner C. Cervicocephalic kinaesthesia in subjects with and without peripherally induced pain. Journal of Musculoskeletal Pain 2013;**21**(4):327-33 Online First. *Ineligible intervention*

218. Rawlins CR, Veigulis Z, Hebert C, Curtin C, Osborne TF. Effect of Immersive Virtual Reality on Pain and Anxiety at a Veterans Affairs Health Care Facility. Frontiers in Virtual Reality 2021;**2** Online First. *Ineligible study design*

219. Reinersmann A, Landwehrt J, Krumova EK, et al. The rubber hand illusion in complex regional pain syndrome: preserved ability to integrate a rubber hand indicates intact multisensory integration. Pain 2013;**154**(9):1519-27 Online First. *Ineligible study design*

220. Richardson EJ, McKinley EC, Rahman AKMF, Klebine P, Redden DT, Richards JS. Effects of virtual walking on spinal cord injury-related neuropathic pain: A randomized, controlled trial. Rehabilitation psychology 2019;**64**(1):13-24 Online First. *Ineligible setting or follow-up*

221. Risso G, Preatoni G, Valle G, Marazzi M, Bracher NM, Raspopovic S. Multisensory stimulation decreases phantom limb distortions and is optimally integrated. iScience 2022;**25**(4):104129 Online First. *Ineligible population*

222. Rivera I, Billington J, Lloyd D. Can virtual reality reduce the perception of pain? Journal of Cyber Therapy and Rehabilitation 2017;**10**(1):51 Online First. *Ineligible study design*

223. Rizzo J-R, Thai P, Li EJ, et al. Structured Wii protocol for rehabilitation of shoulder impingement syndrome: A pilot study. Annals of physical and rehabilitation medicine 2017;**60**(6):363-70 Online First. *Ineligible study design*

224. Rodriguez MC, Aruanno B, Bordegoni M, Rossini M, Molteni F. Immersive virtual reality system for treatment of phantom limb pain (plp). Proceedings of the ASME Design Engineering Technical Conference 2017;**1** Online First. *Ineligible population*

225. Rodriguez S, Badia SB, Cameirao MD, et al. Effects of virtual reality upper limb based training (rehabilitation gaming system) on spasticity, shoulder pain depression after stroke. PM and R 2011;**3**(10 SUPPL. 1):S160 Online First. *Ineligible study design*

226. Rodriguez-Mansilla J, Mejias-Gil A, Garrido-Ardila EM, Jimenez-Palomares M, Montanero-Fernandez J, Gonzalez-Lopez-Arza MV. Effects of Non-Pharmacological Treatment on Pain, Flexibility, Balance and Quality of Life in Women with Fibromyalgia: A Randomised Clinical Trial. Journal of clinical medicine 2021;**10**(17) Online First. *Ineligible intervention*

227. Rothbaum AO, Tannenbaum LR, Zimand E, Rothbaum BO. A pilot randomized controlled trial of virtual reality delivered relaxation for chronic low back pain. Virtual Reality 2023 Online First. *Ineligible study design*

228. Saadon-Grosman N, Arzy S. Virtual reality may relieve pain in patients with spinal cord injury. Neurology 2017;**89**(18):e227-e30 Online First. *Ineligible study design*

229. Saito K, Miyaki T, Rekimoto J. The method of reducing phantom limb pain using optical see-through head mounted display. 26th IEEE Conference on Virtual Reality and 3D User Interfaces, VR 2019 - Proceedings 2019:1560-62 Online First. *Ineligible study design*

230. Saito K, Okada A, Matsumura Y, Rekimoto J. Remote Treatment System of Phantom Limb Pain by Displaying Body Movement in Shared VR Space. ACM International Conference Proceeding Series 2020 Online First. *Ineligible study design*

231. Sandal LF, Stochkendahl MJ, Svendsen MJ, et al. An App-Delivered Self-Management Program for People With Low Back Pain: Protocol for the selfBACK Randomized Controlled Trial. JMIR research protocols 2019;**8**(12):e14720 Online First. *Ineligible study design*

232. Sargut M, Schorn S, Novotny A, et al. The effect of Virtual Reality (VR) on anxiety and pain in patients undergoing port implantation Preliminary results of a feasibility study. Current Directions in Biomedical Engineering 2022;**8**(1):97-100 Online First. *Ineligible population*

233. Sarig Bahat H, Weiss PLT, Laufer Y. Characteristics of cervical motion affected by neck pain, as assessed in a virtual environment. Physiotherapy (United Kingdom) 2011;**97**(SUPPL. 1):eS1097-eS98 Online First. *Ineligible study design*

234. Sarig-Bahat H, Takasaki H, Chen X, Betor Y, Treleaven J. Cervical kinematic training with and without an interactive virtual reality device for chronic neck pain-a pilot randomized clinical trial. Physiotherapy (United Kingdom) 2015;**101**(SUPPL. 1):eS1535-eS36 Online First. *Ineligible study design*

235. Schneider SM, Hood LE. Virtual reality: a distraction intervention for chemotherapy. Oncology nursing forum 2007;**34**(1):39 Online First. *Ineligible population*

236. Schroeder D, Korsakov F, Jolton J, Keefe FJ, Haley A, Keefe DF. Creating widely accessible spatial interfaces: mobile VR for managing persistent pain. IEEE computer graphics and applications 2013;**33**(3):82-9 Online First. *Ineligible study design*

237. Schwartz I, Safran O, Karniel N, et al. Positive Effect of Manipulated Virtual Kinematic Intervention in Individuals with Traumatic Stiff Shoulder: A Pilot Study. Journal of clinical medicine 2022;**11**(13) Online First. *Ineligible study design*

238. Seo M, Jeon S, Kim B, Ryu J, Lee K. Pain perception and visual feedback in virtual reality. IBRO Reports 2019;**6**(Supplement):S323 Online First. *Ineligible study design*

239. Shahid Khan MU, Hussain HG, Farooq M, et al. The potential of virtual reality for rehabilitation intervention in females with breast cancer. International Journal of Surgery 2022;**106** Online First. *Ineligible study design*

240. Shahrbanian S. Virtual reality training or physical activity? Which one is more effective in reducing pain among persons with multiple sclerosis? A randomized controlled trial. Multiple Sclerosis Journal 2017;**23**(3 Supplement 1):959-60 Online First. *Ineligible study design*

241. Shahrbanian S, Simmonds MJ. Effects of different virtual reality environments on experimental pain rating in post-stroke individuals with and without pain in comparison to pain free healthy individuals. Annual Review of CyberTherapy and Telemedicine 2008;**6**:53-59 Online First. *Ineligible study design*

242. Shanbehzadeh M, Orooji A, Kazemi-Arpanahi H. Comparing of data mining techniques for predicting in-hospital mortality among patients with covid-19. Journal of Biostatistics and Epidemiology 2021;**7**(2):154-69 Online First. *Ineligible population*

243. Shaw M, Palmeri M, Krupp L, Charvet L. Acute and lasting benefits of a virtual reality in multiple sclerosis. Neurology 2019;**92**(15 Supplement 1) Online First. *Ineligible population*

244. Sherrard C, Parker D, Negus J, McIntosh B. The effects of a Wii Fit-based balance program on knee pain and function in patellofemoral pain sufferers. Journal of Science and Medicine in Sport 2010;**13**(SUPPL. 1):e6-e7 Online First. *Ineligible study design*

245. Silva Ribeiro NM, Ferraz DD, Pedreira E, et al. Virtual rehabilitation via Nintendo Wii R and conventional physical therapy effectively treat post-stroke hemiparetic patients. Topics in stroke rehabilitation 2015;**22**(4):299-305 Online First. *Ineligible population*

246. Smith V, Warty RR, Kashyap R, et al. A randomised controlled trial to assess the feasibility of utilising virtual reality to facilitate analgesia during external cephalic version. Scientific reports 2020;**10**(1):3141 Online First. *Ineligible population*

247. Sobie T. Virtual reality bonestm and feldenkraismovements compared to core stabilization biofeedback and motor control exercises: Comparative effects on chronic nonspecific low back pain in an outpatient clinical setting: A dissertation study and randomized controlled trial. Global Advances in Health and Medicine 2020;**9**:104-05 Online First. *Ineligible study design*

248. Solca M, Ronchi R, Bello-Ruiz J, et al. Heartbeat-enhanced immersive virtual reality to treat complex regional pain syndrome. Neurology 2018;**91**(5):e479-e89 Online First. *Ineligible study design*

249. Soler D, Kumru H, Vidal J, et al. Transcranial direct current stimulation (TDCS) and virtual reality (VR) techniques for treatment neuropathic central pain in spinal cord injury (NP-SCI). European Journal of Pain Supplements 2010;**4**(1):105-06 Online First. *Ineligible study design*

250. Soler D, Morina D, Kumru H, Vidal J, Navarro X. Transcranial Direct Current Stimulation and Visual Illusion Effect According to Sensory Phenotypes in Patients With Spinal Cord Injury and Neuropathic Pain. The journal of pain 2021;**22**(1):86-96 Online First. *Ineligible study design*

251. Soler MD, Kumru H, Pelayo R, et al. Effectiveness of transcranial direct current stimulation and visual illusion on neuropathic pain in spinal cord injury. Brain : a journal of neurology 2010;**133**(9):2565-77 Online First. *Ineligible intervention*

252. Stamm O, Dahms R, Reithinger N, Rus A, Muller-Werdan U. Virtual reality exergame for supplementing multimodal pain therapy in older adults with chronic back pain: a randomized controlled pilot study. Virtual reality 2022;**26**(4):1291-305 Online First. *Ineligible population*

253. Stanmore EK, Mavroeidi A, e Jong LD, et al. The effectiveness and cost-effectiveness of strength and balance Exergames to reduce falls risk for people aged 55 years and older in UK assisted living facilities: a multi-centre, cluster randomised controlled trial. BMC medicine 2019;**17**(1):49 Online First. *Ineligible population*

254. Tasci Z, Cirak Y, Elbasi N, Tutuneken YE, Pamukcu B, Asalioglu A. The effects of virtual reality on pulmonary functions, functional level and pain in the early period after open heart surgery. European Respiratory Journal 2020;**56**(Supplement 64) Online First. *Ineligible population*

255. Tefertiller C, Ketchum JM, Bartelt P, Peckham M, Hays K. Feasibility of virtual reality and treadmill training in traumatic brain injury: a randomized controlled pilot trial. Brain Injury 2022;**36**(7):898-908 Online First. *Ineligible population*

256. Teremetz M, Garcia Alvarez A, Hanneton S, et al. Improving upper-limb and trunk kinematics by interactive gaming in individuals with chronic stroke: A single-blinded RCT. Annals of physical and rehabilitation medicine 2022;**65**(3):101622 Online First. *No eligible outcomes provided*

257. Tran Y, Austin P, Lo C, et al. An Exploratory EEG Analysis on the Effects of Virtual Reality in People with Neuropathic Pain Following Spinal Cord Injury. Sensors (Basel, Switzerland) 2022;**22**(7) Online First. *Ineligible population*

258. Trost Z, Nowlin L, Guck A, Madi D, Davis M. Exploring the role of pain-related fear and catastrophizing in response to a virtual reality gaming intervention for chronic low back pain. Journal of Pain 2015;**16**(4 SUPPL. 1):S98 Online First. *Ineligible study design*

259. Trujillo M, Crossland D, Alvarez A, Petros J. Virtual embodiment influences dynamic range of motion in chronic shoulder pain patients. Neuromodulation 2020;**23**(3):e179 Online First. *Ineligible study design*

260. Tuck N, Pollard C, Good C, et al. Active Virtual Reality for Chronic Primary Pain: Mixed Methods Randomized Pilot Study. JMIR formative research 2022;**6**(7):e38366 Online First. *Ineligible study design*

261. Ucero-Lozano R, Perez-Llanes R, Lopez-Pina JA, Cuesta-Barriuso R. Approach to Knee Arthropathy through 180-Degree Immersive VR Movement Visualization in Adult Patients with Severe Hemophilia: A Pilot Study. Journal of clinical medicine 2022;**11**(20) Online First. *Ineligible population*

262. Ucero-Lozano R, Perez-Llanes R, Lopez-Pina JA, Cuesta-Barriuso R. Effectiveness of Motion Visualization Through Immersive Video in Patients With Hemophilic Arthropathy Of The Ankle. Archives of Physical Medicine and Rehabilitation 2022;**103**(12):e167 Online First. *Ineligible study design*

263. Ucero-Lozano R, Perez-Llanes R, Lopez-Pina JA, Cuesta-Barriuso R. IMMERSIVE VR VISUALIZATION to IMPROVE QUALITY of LIFE in PATIENTS with HEMOPHILIC ANKLE ARTHROPATHY. Haemophilia 2022;**28**(SUPPL 1):23-24 Online First. *Ineligible study design*

264. Ucero-Lozano R, Perez-Llanes R, Lopez-Pina JA, Cuesta-Barriuso R. 180-degree immersive VR motion visualization in the treatment of haemophilic ankle arthropathy. Haemophilia : the official journal of the World Federation of Hemophilia 2023;**29**(1):282-89 Online First. *Ineligible study design*

265. Vázques JLM, Golsong JB, Wiederhold MD. Cybertherapy in medicine - Clinical applications to reduce pain and anxiety. Journal of Cyber Therapy and Rehabilitation 2013;**6**(1):28-29 Online First. *Ineligible study design*

266. Venuturupalli RS, Chu T, Vicari M, Kumar A, Fortune N, Spielberg B. Virtual Reality-Based Biofeedback and Guided Meditation in Rheumatology: A Pilot Study. ACR open rheumatology 2019;**1**(10):667-75 Online First. *Ineligible setting or follow-up*

267. Vilalta-Abella F, Gutierrez-Maldonado J, Pla-Sanjuanelo J. Development Of A Virtual Environment Based On The Perceived Characteristics Of Pain In Patients With Fibromyalgia. Studies in health technology and informatics 2015;**219**:158-62 Online First. *Ineligible study design*

268. Villiger M, Bohli D, Kiper D, et al. Virtual reality training for the rehabilitation of lower limb motor dysfunction and neuropathic pain after spinal cord injury. Neurorehabilitation and Neural Repair 2012;**26**(4):398 Online First. *Ineligible study design*

269. Vugts MA, Zedlitz AM, Joosen MC, Vrijhoef HJ. Serious Gaming During Multidisciplinary Rehabilitation for Patients With Chronic Pain or Fatigue Symptoms: Mixed Methods Design of a Realist Process Evaluation. Journal of medical Internet research 2020;**22**(3):e14766 Online First. *Ineligible study design*

270. Vugts MAP, Joosen MCW, Mert A, Zedlitz A, Vrijhoef HJM. Serious gaming during multidisciplinary rehabilitation for patients with complex chronic pain or fatigue complaints: study protocol for a controlled trial and process evaluation. BMJ open 2017;**7**(6):e016394 Online First. *Ineligible study design*

271. Wake N, Sano Y, Oya R, Sumitani M, Kumagaya SI, Kuniyoshi Y. Multimodal virtual reality platform for the rehabilitation of phantom limb pain. International IEEE/EMBS Conference on Neural Engineering, NER 2015;**2015-July**:787-90 Online First. *Ineligible study design*

272. Walter S, Traue HC. Differential effects in the intervention with different forms of virtual reality in the psychological pain treatment. Verhaltenstherapie und Verhaltensmedizin 2015;**36**(1):66-68 Online First. *Not published in English*

273. Wang J, Anslow C, Robinson B, McCallum S. Extended reality for chronic pain relief. Proceedings of the ACM Symposium on Virtual Reality Software and Technology, VRST 2019 Online First. *Ineligible study design*

274. Wiederhold BK, Gao K, Kong L, Wiederhold MD. Mobile devices as adjunctive pain management tools. Cyberpsychology, behavior and social networking 2014;**17**(6):385-9 Online First. *Ineligible study design*

275. Wiederhold BK, Miller IT, Wiederhold MD. Using Virtual Reality to Mobilize Health Care: Mobile Virtual Reality Technology for Attenuation of Anxiety and Pain. IEEE Consumer Electronics Magazine 2018;**7**(1):106-09 Online First. *Ineligible study design*

276. Wiederhold BK, Riva G, Wiederhold MD. How can virtual reality interventions help reduce prescription opioid drug misuse? Cyberpsychology, Behavior, and Social Networking 2014;**17**(6):331-32 Online First. *Ineligible study design*

277. Wilson AS, Ramesh A, Carruthers D, connor J. A prototype virtual reality rehabilitation game for complex regional pain syndrome. Proceedings of the European Conference on Games-based Learning 2018;**2018-October**:745-53 Online First. *Ineligible population*

278. Winkler SL, Kairalla JA, Cooper R, et al. Comparison of functional benefits of self-management training for amputees under virtual world and e-learning conditions. Journal of Alternative Medicine Research 2018;**10**(1):65-72 Online First. *Ineligible intervention*

279. Won AS, Barreau AC, Gaertner M, et al. Assessing the Feasibility of an Open-Source Virtual Reality Mirror Visual Feedback Module for Complex Regional Pain Syndrome: Pilot Usability Study. Journal of medical Internet research 2021;**23**(5):e16536 Online First. *Ineligible study design*

280. Wood DP, Wiederhold MD, Spira J, Scholl D, Pyne J, Wiederhold BK. Cybertherapy for combat related posttraumatic stress disorder, pain management and physical rehabilitation following stroke. Coping with posttraumatic stress disorder in returning troops: Wounds of war II. 2010:169-84 Online First. *Ineligible study design*

281. Wright C, Babber A, Price S. USING VIRTUAL REALITY IN PATIENTS WITH PALLIATIVE CARE NEEDS. BMJ Supportive and Palliative Care 2019;**9**(Supplement 4):A71 Online First. *Ineligible study design*

282. Xie J, Zeng Q. Application of virtual reality technology combined with moderate perineal protection in natural childbirth. Ginekologia polska 2022 Online First. *Ineligible setting or follow-up*

283. Yanagisawa T, Fukuma R, Seymour B, et al. BCI training to move a virtual hand reduces phantom limb pain: A randomized crossover trial. Neurology 2020;**95**(4):E417-E26 Online First. *Ineligible study design*

284. Yilmaz DS, Baki AE. Effect of game based exercise programs on pain, functional mobility and balance in patients with knee osteoarthritis: Randomized controlled study. Annals of the Rheumatic Diseases 2019;**78**(Supplement 2):498-99 Online First. *Ineligible study design*

285. Yohannan SK, Tufaro PA, Hunter H, et al. The efficacy of Nintendo WiiTM during post-burn rehabilitation. Journal of Burn Care and Research 2011;**32**(SUPPL. 2):S66 Online First. *Ineligible study design*

286. Yohannan SK, Tufaro PA, Hunter H, et al. The utilization of Nintendo R Wii TM during burn rehabilitation: a pilot study. Journal of burn care & research : official publication of the American Burn Association 2012;**33**(1):36-45 Online First. *Ineligible study design*

287. Zavala J, Martínez D, Gutiérrez H. Effects of virtual reality added to physiotherapy training in patients with total knee arthroplasty. Fisioterapia 2019;**41**(6):322-28 Online First. *Ineligible study design*

288. Zeher MJ, Armiger RS, Burck JM, et al. Using a virtual integration environment in treating phantom limb pain. Studies in Health Technology & Informatics 2011;**164**:730-36 Online First. *Ineligible study design*

289. Zhang C. Application effect of virtual reality technology in postoperative pain of patients with vocal cord disease. Chinese Evidence-based Nursing 2021;**7**(13):1794-97 Online First. *Not published in English*
