# Supplementary material for: Extended Reality Interventions for Osteoarthritis of the Knee and Recovery After Total Knee Arthroplasty: Systematic Review and Meta-Analyses
Source: J Med Internet Res. 2026 Sep 8;28:e84753. doi: 10.2196/84753 (PMC13555783; doi:10.2196/84753)
Supplement: Supplementary file 2 [file jmir-v28-e84753-s004.docx]

**OA Studies – Equipment**

| **Author, Year** | **Author-reported XR Device/Hardware** |
| --- | --- |
| **VR** | |
| Lo et al, 2024[39] | A smart phone app (iOS or Android) which captured outdoor garden scenes was used to create an immersive environment and participants were immersed using a head-mounted display (HMD) device (VR Shinecon 5.0). |
| Ozlu et al, 2023[45] | Quest 128 GB type of Oculus brand immersive glasses. |
| **IDR** | |
| Buyukyilmaz et al, 2024[28] | WBE program was implemented with the Becure Balance System. Becure (Becure Global, Mannheim, Germany) is a web-based platform designed for the field of physiotherapy, incorporating both assessment tools and rehabilitative games. The Becure balance system includes custom rehabilitative video games developed by physiotherapists and software developers using a Wii balance board. |
| Carvajal-Parodi et al, 2025[29]; Guede-Rojas et al, 2025[34] | Nintendo Switch, on a 43-in television |
| Dal Erdogan et al, 2025[30] | The BTS Nirvana is a device that analyzes a patient’s movements in a virtual environment with optoelectronic infrared sensors. The system is projected onto a main screen via a projector. |
| Elshazly et al, 2016[32] | "Light Race consists of its player standing on a virtual platform displayed on the game screen and doing steps on the platform by moving his/her left or right lower limbs back and forth and left and right according to the signs." |
| Jarungvittayakon et al, 2025[36] | A wearable sensor instrument and a mobile game. The sensor instrument consisted of two plastic boxes sized at 4 × 3 × 1 cm containing an IMU sensor, internal chargeable battery, and a Wi-Fi transmitter. The first plastic box was attached 10 cm above the knee at the anterior thigh, and the other was attached 10 cm below the knee at the lateral calf. Both boxes are connected to each other via a Wi-Fi hotspot and can measure the relative angle between them using IMU sensors. The first device measures the angle relative to the ground, while the second device measures the angle relative to the Earth’s vertical axis, based on their respective positions. The program then calculates the angular difference between the two devices to display the knee flexion– extension angle. The sensors connect to a smartphone through the same Wi-Fi hotspot, and the game-based program is developed as a mobile application compatible with both iOS and Android systems. All data, including usage time and knee flexion–extension angles, are automatically synchronized with an online cloud database for patient data storage. |
| Lin et al, 2020[38] | Television screen and Hot Plus video game system (has step-sensing pad placed on the floor). |
| Mete et al, 2022[41] | Joint education device (MarVAJED, Marmara Visual Auditory Joint Education Device) , developed by Marmara University. This device evaluated the range of motion of the joints and provides auditory and visual biofeedback support to increase joint control. The device has sensors placed above and under the participant knee and was calibrated to allow participants to play virtual games through the flexion and extension of the knee. |
| Nambi et al, 2020[42] | Pro-Kin system PK 252 N Techno body, Italy |
| Oliveira et al, 2024[44] | Training using the Xbox 360 videogame with Microsoft Kinect 360 sensor, with a 42† TV set connected to the consoles. |
| Sonmez et al, 2025[49] | Microsoft Kinect Xbox 360 |

**TKA Studies - Equipment**

| **Author, Year** | **Author-reported XR Device/Hardware** |
| --- | --- |
| **VR** | |
| Fuchs, 2022[33] | "Head mounted display that allows projection of a three-dimensional image (Samsung Gear VR) (Fig. 1)... CPM (Continuous passive motion device) (Fig. 2)." |
| Jin, 2018[37] | VR headset (Mide Tech) |
| Mazurek, 2023[40] | VRTierOne device by Stolgraf®, Stanowice, Poland. This system employed VR HTC VIVE goggles (2017) and two controllers. |
| Nishitha, 2024[43] | Sensors were inserted onto limbs, 3D-head-mounted glasses were utilised. The games are loaded from game engine software. |
| **IDR** | |
| Eichler, 2019[31] | "The rehab box (minicomputer with internet access) was connected to the usual peripherals (mouse and keyboard) as well as to a screen and Kinect sensor (camera)..." |
| Janhunen, 2023[35] | Kinect 2.0 connected to a laptop and controlled with a tablet (Lenovo), television screens in the homes of the study participants |
| Piqueras, 2013[46] | "A touch-screen all-in-one computer... wireless system to capture patient movements, the core of which is the two sensors, and one low-bandwidth mobile internet device... A tool kit with the items necessary to perform the exercises: straps for attaching sensors (one placed above the knee and another on the ankle), a set of weights and a stretch band." |
| Prvu Bettger, 2020[47] | Virtual Exercise Rehabilitation Assistant (VERA; Reflexion Health) system |
| Shim, 2023[48] | Xbox One Kinect, a computer, and a display (TV or monitor) |

References

28. Büyükyilmaz G, Menek B, Tarakçi D. Closed kinetic chain exercises therapy versus Wii-based Exergame therapy in the treatment of knee osteoarthritis: randomized controlled trial. Arch Health Sci Res. 2024;11(2):133-139. [doi: 10.5152/ArcHealthSciRes.2024.23139]

29. Carvajal-Parodi C, Mendoza C, Alvarez C, et al. Effectiveness of exergames on functional physical performance in older adults with knee/hip osteoarthritis: a randomized controlled trial. J Clin Med. Apr 25, 2025;14(9):2968. [doi: 10.3390/jcm14092968] [Medline: 40363999]

30. Dal Erdoğan S, Berkan F, Armağan O, Özgen M, Çiracioğlu AM, Şahi̇n Mutlu F. The effects of virtual reality-based exercise on pain and function in older adults with knee osteoarthritis: a randomized controlled study. Turk J Geriatr. 2025;28(2). [doi: 10.29400/tjgeri.2025.432]

31. Eichler S, Salzwedel A, Rabe S, et al. The effectiveness of telerehabilitation as a supplement to rehabilitation in patients after total knee or hip replacement: randomized controlled trial. JMIR Rehabil Assist Technol. Nov 7, 2019;6(2):e14236. [doi: 10.2196/14236] [Medline: 31697239]

[32. Elshazly FAA, Nambi GS, Elnegamy TE. Comparative study on virtual reality training (VRT) over sensory motor training (SMT) in unilateral chronic osteoarthritis—A randomized control trial. Int J Med Res Health Sci. 2016;5(8):7-16. URL: https://www.ijmrhs.com/](https://www.ijmrhs.com/)

33. Fuchs L, Kluska A, Novak D, Kosashvili Y. The influence of early virtual reality intervention on pain, anxiety, and function following primary total knee arthroplasty. Complement Ther Clin Pract. Nov 2022;49:101687. [doi: 10.1016/j.ctcp.2022.101687] [Medline: 36347152]

34. Guede-Rojas F, Mendoza C, Rodríguez-Lagos L, et al. Effects of non-immersive virtual reality exercise on self-reported pain and mechanical hyperalgesia in older adults with knee and hip osteoarthritis: a secondary analysis of a randomized controlled trial. Medicina (B Aires). 2025;61(7):1122. [doi: 10.3390/medicina61071122]

35. Janhunen M, Katajapuu N, Paloneva J, et al. Effects of a home-based, exergaming intervention on physical function and pain after total knee replacement in older adults: a randomised controlled trial. BMJ Open Sport Exerc Med. 2023;9(1):e001416. [doi: 10.1136/bmjsem-2022-001416] [Medline: 36896366]

36. Jarungvittayakon C, Sa-Ngasoongsong P, Chockchaisakul D, Bamrungchaowkasem J, Wongsak S. Effectiveness of mobile exergaming with sensor-based visual feedback as an adjunct therapy for home-based quadriceps exercise training in knee osteoarthritis: a prospective randomized controlled trial. Life (Basel). Nov 12, 2025;15(11):1738. [doi: 10.3390/life15111738] [Medline: 41302162]

[37. Jin C, Feng Y, Ni Y, Shan Z. Virtual reality intervention in postoperative rehabilitation after total knee arthroplasty: A prospective and randomized controlled clinical trial. Int J Clin Exp Med. 2018;11(6):6119-6124. URL: www.ijcem.com](www.ijcem.com)

38. Lin YT, Lee WC, Hsieh RL. Active video games for knee osteoarthritis improve mobility but not WOMAC score: a randomized controlled trial. Ann Phys Rehabil Med. Nov 2020;63(6):458-465. [doi: 10.1016/j.rehab.2019.11.008] [Medline: 31981832]

39. Lo HHM, Ng M, Fong PYH, et al. Examining the feasibility, acceptability, and preliminary efficacy of an immersive virtual reality-assisted lower limb strength training for knee osteoarthritis: mixed methods pilot randomized controlled trial. JMIR Serious Games. Sep 27, 2024;12:e52563. [doi: 10.2196/52563] [Medline: 39331525]

40. Mazurek J, Cieślik B, Wrzeciono A, Gajda R, Szczepańska-Gieracha J. Immersive virtual reality therapy is supportive for orthopedic rehabilitation among the elderly: a randomized controlled trial. J Clin Med. Dec 14, 2023;12(24):7681. [doi: 10.3390/jcm12247681] [Medline: 38137750]

41. Mete E, Sari Z. The efficacy of exergaming in patients with knee osteoarthritis: a randomized controlled clinical trial. Physiother Res Int. Jul 2022;27(3):e1952. [doi: 10.1002/pri.1952] [Medline: 35470534]

42. Nambi G, Abdelbasset WK, Elsayed SH, Khalil MA, Alrawaili SM, Alsubaie SF. Comparative effects of virtual reality training and sensory motor training on bone morphogenic proteins and inflammatory biomarkers in post-traumatic osteoarthritis. Sci Rep. Sep 28, 2020;10(1):15864. [doi: 10.1038/s41598-020-72587-2] [Medline: 32985509]

43. Nishitha K, Anitha A, Thaheera D. Effectiveness of virtual reality-based rehabilitation and high-intensity exercise program for total knee arthroplasty patients: a randomised controlled trial. J Clin Diagn Res. 2024;18(11). [doi: 10.7860/JCDR/2024/71122.20263]

44. Oliveira LKR, Marques AP, Andrade KFA, et al. Virtual reality in improving anticipatory postural adjustments to step initiation in individuals with knee osteoarthritis: a randomized controlled trial. Games Health J. Apr 2024;13(2):100-108. [doi: 10.1089/g4h.2023.0154] [Medline: 38436585]

45. Özlü A, Ünver G, Tuna Hİ, Menekşeoğlu AK. The effect of a virtual reality-mediated gamified rehabilitation program on pain, disability, function, and balance in knee osteoarthritis: a prospective randomized controlled study. Games Health J. Apr 2023;12(2):118-124. [doi: 10.1089/g4h.2022.0130] [Medline: 36603100]

46. Piqueras M, Marco E, Coll M, et al. Effectiveness of an interactive virtual telerehabilitation system in patients after total knee arthoplasty: a randomized controlled trial. J Rehabil Med. Apr 2013;45(4):392-396. [doi: 10.2340/16501977-1119] [Medline: 23474735]

47. Prvu Bettger J, Green CL, Holmes DN, et al. Effects of virtual exercise rehabilitation in-home therapy compared with traditional care after total knee arthroplasty: VERITAS, a randomized controlled trial. J Bone Joint Surg Am. Jan 15, 2020;102(2):101-109. [doi: 10.2106/JBJS.19.00695] [Medline: 31743238]

48. Shim GY, Kim EH, Lee SJ, et al. Postoperative rehabilitation using a digital healthcare system in patients with total knee arthroplasty: a randomized controlled trial. Arch Orthop Trauma Surg. Oct 2023;143(10):6361-6370. [doi: 10.1007/s00402-023-04894-y] [Medline: 37129691]

49. Sönmez M, Avcı Ş, Şimşek F, Baygutalp F. Nonimmersive virtual reality-based exercises improve muscle excitability and balance in patients with knee osteoarthritis: a sham-controlled study. Games Health J. Dec 2025;14(6):493-503. [doi: 10.1177/2161783X251360443] [Medline: 40711924]
