# Supplementary material for: Extended Reality Interventions for Osteoarthritis of the Knee and Recovery After Total Knee Arthroplasty: Systematic Review and Meta-Analyses
Source: J Med Internet Res. 2026 Sep 8;28:e84753. doi: 10.2196/84753 (PMC13555783; doi:10.2196/84753)
Supplement: Supplementary file 3 [file jmir-v28-e84753-s005.docx]

**High certainty**: we are very confident that the true effect lies close to that of the estimate of the effect.
**Moderate certainty**: we are moderately confident in the effect estimate: the true effect is likely to be close to the estimate of the effect, but there is a possibility that it is substantially different.
**Low certainty**: our confidence in the effect estimate is limited: the true effect may be substantially different from the estimate of the effect.
**Very low certainty**: we have very little confidence in the effect estimate: the true effect is likely to be substantially different from the estimate of effect.
