# Supplementary material for: Extended Reality Interventions for Osteoarthritis of the Knee and Recovery After Total Knee Arthroplasty: Systematic Review and Meta-Analyses
Source: J Med Internet Res. 2026 Sep 8;28:e84753. doi: 10.2196/84753 (PMC13555783; doi:10.2196/84753)
Supplement: Supplementary file 4 [file jmir-v28-e84753-s006.docx]

VR-DAE for Knee OA Pain

| **Outcome**  Outcome Measure | Follow-up  No. of Participants (Studies) | **Anticipated Absolute Effects** | | | Certainty | What Happens |
| --- | --- | --- | --- | --- | --- | --- |
|  |  | **VR** | **Comparator** | **Difference** |  |  |
| **Pain-Related Functioning or Interference**  WOMAC total | 3 weeks  N = 73  1 RCT[45] | -8.6* | 0.1* | **Diff** Δ**:**  **-8.7*** | ⨁⨁◯◯  Low^a^ | VR physical activity may result in better pain-related functioning compared with conventional exercise. |
| **Pain-Related Functioning or Interference**  WOMAC total | 6-7 weeks  N = 103  2 RCTs[39, 45] | 23.8^†^ | 30.0^†^ | **Diff** Δ**:**  **-6.2**^†^ | ⨁◯◯◯  Very Low^a,b^ | The evidence is very uncertain on the effect of VR physical activity on pain-related functioning compared with conventional exercise. |
| **Pain-Related Functioning or Interference**  WOMAC total | 12 weeks  N = 30  1 RCT[39] | -111.6^†^ | -9.6^†^ | **Diff** Δ**:**  **-52.7**^†^ | ⨁⨁◯◯  Low^c^ | VR physical activity may result in better pain-related functioning compared with conventional exercise. |
| **Pain Intensity or Severity**  VAS | 3 weeks  N = 73  1 RCT[39] | -1.46* | -0.73* | **Diff** Δ**:**  **-0.73*** | ⨁⨁◯◯  Low^a^ | VR physical activity may result in lower pain intensity compared with conventional exercise. |
| **Pain Intensity or Severity**  NRS, VAS | 6-7 weeks  N = 103  2 RCTs[39, 45] | -0.4^†^ | 0.3^†^ | **Diff** Δ**:**  **-0.7**^†^ | ⨁◯◯◯  Very Low^a,b^ | The evidence is very uncertain on the effect of VR physical activity on pain intensity compared with conventional exercise. |
| **Pain Intensity or Severity**  NRS | 12 weeks  N = 30  1 RCT[39] | -1.1^†^ | -0.2^†^ | **Diff** Δ**:**  **-0.9**^†^ | ⨁⨁⨁◯  Moderate^c^ | VR physical activity probably results in lower pain intensity compared with conventional exercise. |
| **Adverse Events** | 12 weeks  N = 30  1 RCT[39] | 0^†^ | 0^†^ | **0**^†^ | ⨁⨁◯◯  Low^c,e^ | VR physical activity may result in little to no difference in adverse events compared with conventional exercise. |
| *Values for mean change in VR physical activity, comparator (conventional therapy) and Diff Δ taken from Ozlu et al[45]  ^†^Values for mean change in VR physical activity, comparator (conventional therapy) and Diff Δ taken from Lo et al[39]  CI**:** confidence interval; Diff Δ: difference in change scores; No.: number; NRS: numeric rating scale; RCT: randomized controlled trial; VAS: Visual Analogue Scale; VR: virtual reality; WOMAC: Western Ontario and McMaster Universities Arthritis Index | | | | | | |

**Explanations**

a. Downgraded 2 levels for study limitations (study rated high risk of bias)

b. Downgraded 1 level for inconsistency (direction of effect varied across studies)

c. Downgraded one level for study limitations (study rated some concerns)

d. Downgraded 1 level for indirectness (adverse event data only reported for one arm in study)

IDR-DAE for Knee OA Pain

| **Outcome**  Outcome Measure | Follow-up  No. of Participants (Studies) | **SMD Pooled Estimate (95% CI)** | **Anticipated Absolute Effects** | | | Certainty | What Happens |
| --- | --- | --- | --- | --- | --- | --- | --- |
|  |  |  | **IDR** | **Comparator** | **Difference** |  |  |
| **Pain-Related Functioning or Interference**  WOMAC total | 3-4 weeks  N = 172  4 RCTs[28, 30, 32, 42] | **SMD:**  **-0.59 (-1.62, 0.43)** | -31.9* | -17.8* | **Diff** Δ**:**  **-14.1*** | ⨁◯◯◯  Very Low^a,b,c,d^ | The evidence is very uncertain on the effect of IDR physical activity on pain-related functioning compared with conventional exercise. |
| **Pain-Related Functioning or Interference**  WOMAC total, KOOS | 6-8 weeks  N = 232  5 RCTs[28, 32, 36, 42, 44] | **SMD:**  **-0.59 (-1.11, -0.06)** | 12.0^†^ | 4.5^†^ | **Diff** Δ**:**  **7.5**^†^ | ⨁⨁◯◯  Low^a,b^ | IDR physical activity may result in better pain-related functioning, compared with conventional exercise. |
| **Pain-Related Functioning or Interference**  WOMAC total | 3 months  N = 37  1 RCT[42] | - | -16.1‡ | -36.0‡ | **Diff** Δ**:**  **-25.1**‡ | ⨁◯◯◯  Very Low^a,b^ | The evidence is very uncertain on the effect of IDR physical activity on pain-related functioning compared with conventional exercise. |
| **Pain Intensity or Severity**  VAS | 3-4 weeks  N = 176  4 RCTs[29, 30, 32, 42] | **SMD:**  **-0.72 (-1.77, 0.34)** | -18.0^§^ | -12.6^§^ | **Diff** Δ**:**  **-5.4**^§^ | ⨁◯◯◯  Very Low^a,b,c,d^ | The evidence is very uncertain on the effect of IDR physical activity on pain intensity compared with conventional exercise. |
| **Pain Intensity or Severity**  VAS | 6-8 weeks  N = 180  4 RCTs[29, 32, 34, 42, 44] | **SMD:**  **-0.46 (-0.92, -0.00)** | -15.8^§^ | -5.9^§^ | **Diff** Δ**:**  **-9.9**^§^ | ⨁⨁◯◯  Low^b,e^ | IDR physical activity may result in lower pain intensity compared with conventional exercise. |
| **Pain Intensity or Severity**  VAS | 12-14 weeks  N = 100  2 RCTs[34, 42] | - | -14.7^§^ | -7.8^§^ | **Diff** Δ**:**  **-6.9**^§^ | ⨁⨁◯◯  Low^b,e^ | IDR physical activity may result in lower pain intensity compared with conventional exercise. |
| **Adverse Events** | 4 weeks  N = 80  1 RCT[38] | - | 0^¶^ | 0^¶^ | **Diff** Δ**:**  ^¶^**0**^¶^ | ⨁⨁◯◯  Low^e,f^ | IDR physical activity may result in little to no difference in adverse events compared with conventional exercise. |
| **Adverse Events** | 14 weeks  N = 60  1 RCT[29] | - | 0^§^ | ^§^0^§^ | **Diff** Δ**:**  ^§^**0**^§^ | ⨁⨁⨁⨁  High | IDR physical activity results in little to no difference in adverse events compared with conventional exercise. |
| *Values for mean change in VR physical activity, comparator (conventional therapy) and Diff Δ taken from Elshazly et al[32]  ^†^Values for mean change in VR physical activity, comparator (conventional therapy) and Diff Δ taken from Jarungvittayakon et al[36]  ^‡^Values for mean change in VR physical activity, comparator (conventional therapy) and Diff Δ taken from Nambi et al[42]  ^§^Values for mean change in VR physical activity, comparator (conventional therapy) and Diff Δ taken from Carvajal-Parodi et al[29, 34]  ^¶^Values for mean change in VR physical activity, comparator (conventional therapy) and Diff Δ taken from Lin et al[38]  CI**:** confidence interval; Diff Δ: difference in change scores; No.: number; NRS: numeric rating scale; RCT: randomized controlled trial; VAS: Visual Analogue Scale; VR: virtual reality; WOMAC: Western Ontario and McMaster Universities Arthritis Index | | | | | | | |

**Explanations**

a. Downgraded 2 levels for study limitations (study rated high risk of bias).

b. Downgraded one level for indirectness (study includes only young male athletes)

c. Downgraded 1 level for inconsistency (effect varies across studies)

d. Downgraded 1 level for imprecision (CI crosses 0).

e. Downgraded one level for study limitations (study rated some concerns)

f. Downgraded 1 level for indirectness (authors do not describe how they measured adverse events)

References

28. Büyükyilmaz G, Menek B, Tarakçi D. Closed kinetic chain exercises therapy versus Wii-based Exergame therapy in the treatment of knee osteoarthritis: randomized controlled trial. Arch Health Sci Res. 2024;11(2):133-139. [doi: 10.5152/ArcHealthSciRes.2024.23139]

29. Carvajal-Parodi C, Mendoza C, Alvarez C, et al. Effectiveness of exergames on functional physical performance in older adults with knee/hip osteoarthritis: a randomized controlled trial. J Clin Med. Apr 25, 2025;14(9):2968. [doi: 10.3390/jcm14092968] [Medline: 40363999]

30. Dal Erdoğan S, Berkan F, Armağan O, Özgen M, Çiracioğlu AM, Şahi̇n Mutlu F. The effects of virtual reality-based exercise on pain and function in older adults with knee osteoarthritis: a randomized controlled study. Turk J Geriatr. 2025;28(2). [doi: 10.29400/tjgeri.2025.432]

[32. Elshazly FAA, Nambi GS, Elnegamy TE. Comparative study on virtual reality training (VRT) over sensory motor training (SMT) in unilateral chronic osteoarthritis—A randomized control trial. Int J Med Res Health Sci. 2016;5(8):7-16. URL: https://www.ijmrhs.com/](https://www.ijmrhs.com/)

34. Guede-Rojas F, Mendoza C, Rodríguez-Lagos L, et al. Effects of non-immersive virtual reality exercise on self-reported pain and mechanical hyperalgesia in older adults with knee and hip osteoarthritis: a secondary analysis of a randomized controlled trial. Medicina (B Aires). 2025;61(7):1122. [doi: 10.3390/medicina61071122]

36. Jarungvittayakon C, Sa-Ngasoongsong P, Chockchaisakul D, Bamrungchaowkasem J, Wongsak S. Effectiveness of mobile exergaming with sensor-based visual feedback as an adjunct therapy for home-based quadriceps exercise training in knee osteoarthritis: a prospective randomized controlled trial. Life (Basel). Nov 12, 2025;15(11):1738. [doi: 10.3390/life15111738] [Medline: 41302162]

38. Lin YT, Lee WC, Hsieh RL. Active video games for knee osteoarthritis improve mobility but not WOMAC score: a randomized controlled trial. Ann Phys Rehabil Med. Nov 2020;63(6):458-465. [doi: 10.1016/j.rehab.2019.11.008] [Medline: 31981832]

39. Lo HHM, Ng M, Fong PYH, et al. Examining the feasibility, acceptability, and preliminary efficacy of an immersive virtual reality-assisted lower limb strength training for knee osteoarthritis: mixed methods pilot randomized controlled trial. JMIR Serious Games. Sep 27, 2024;12:e52563. [doi: 10.2196/52563] [Medline: 39331525]

42. Nambi G, Abdelbasset WK, Elsayed SH, Khalil MA, Alrawaili SM, Alsubaie SF. Comparative effects of virtual reality training and sensory motor training on bone morphogenic proteins and inflammatory biomarkers in post-traumatic osteoarthritis. Sci Rep. Sep 28, 2020;10(1):15864. [doi: 10.1038/s41598-020-72587-2] [Medline: 32985509]

44. Oliveira LKR, Marques AP, Andrade KFA, et al. Virtual reality in improving anticipatory postural adjustments to step initiation in individuals with knee osteoarthritis: a randomized controlled trial. Games Health J. Apr 2024;13(2):100-108. [doi: 10.1089/g4h.2023.0154] [Medline: 38436585]

45. Özlü A, Ünver G, Tuna Hİ, Menekşeoğlu AK. The effect of a virtual reality-mediated gamified rehabilitation program on pain, disability, function, and balance in knee osteoarthritis: a prospective randomized controlled study. Games Health J. Apr 2023;12(2):118-124. [doi: 10.1089/g4h.2022.0130] [Medline: 36603100]
