# Supplementary material for: Extended Reality Interventions for Osteoarthritis of the Knee and Recovery After Total Knee Arthroplasty: Systematic Review and Meta-Analyses
Source: J Med Internet Res. 2026 Sep 8;28:e84753. doi: 10.2196/84753 (PMC13555783; doi:10.2196/84753)
Supplement: Supplementary file 5 [file jmir-v28-e84753-s007.docx]

VR-DAE for Post-TKA

| **Outcome**  Outcome Measure | Follow-up  No. of Participants (Studies) | **Anticipated Absolute Effects** | | | Certainty | What Happens |
| --- | --- | --- | --- | --- | --- | --- |
|  |  | **VR** | **Comparator** | **Difference** |  |  |
| **Pain-Related Functioning or Interference**  WOMAC total | 3 months  N = 102  2 RCTs[37, 43] | -19.2* | -14.5* | **Diff** Δ**:**  **-4.7*** | ⨁⨁◯◯  Low^a^ | VR physical activity may result in better pain-related functioning compared with conventional exercise. |
| **Pain-Related Functioning or Interference**  WOMAC total | 6 months  N = 66  1 RCT[37] | -23.5* | -17.9* | **Diff** Δ**:**  **-5.6*** | ⨁⨁◯◯  Low^a^ | The evidence is very uncertain on the effect of VR physical activity on pain intensity compared with conventional exercise. |
| **Pain Intensity or Severity**  VAS | 7 days  N = 66  1 RCT[37] | -3.5* | -3.0* | **Diff** Δ**:**  **-0.5*** | ⨁⨁◯◯  Low^a^ | VR physical activity may result in decreased pain intensity compared with conventional exercise. |
| **Pain Intensity or Severity**  NRS | 4 weeks  N = 36  1 RCT[43] | -5.2^†^ | -4.6^†^ | **Diff** Δ**:**  **-0.5**^†^ | ⨁⨁◯◯  Low^a^ | VR physical activity may result in decreased pain intensity compared with conventional exercise. |
| **Pain Intensity or Severity**  NRS | 3 months  N = 36  1 RCT[43] | -6.1^†^ | -5.1^†^ | **Diff** Δ**:**  **-0.9**^†^ | ⨁⨁◯◯  Low^a^ | VR physical activity may result in decreased pain intensity compared with conventional exercise. |
| *Values for mean change in VR physical activity, comparator (conventional therapy) and Diff Δ taken from Jin et al[37]  ^†^Values for mean change in VR physical activity, comparator (conventional therapy) and Diff Δ taken from Nishitha et al[43]  CI**:** confidence interval; Diff Δ: difference in change scores; No.: number; NRS: numeric rating scale; RCT: randomized controlled trial; VAS: Visual Analogue Scale; VR: virtual reality; WOMAC: Western Ontario and McMaster Universities Arthritis Index | | | | | | |

**Explanations**

a. Downgraded 2 levels for study limitations (study rated high risk of bias

VR-Distraction for Post-TKA

| **Outcome**  Outcome Measure | Follow-up  No. of Participants (Studies) | **Anticipated Absolute Effects** | | | Certainty | What Happens |
| --- | --- | --- | --- | --- | --- | --- |
|  |  | **VR** | **Comparator** | **Difference** |  |  |
| **Pain-Related Functioning or Interference**  Modified KOOS | 6 months  N = 55  1 RCT[33] | 20.8* | 19.7* | **Diff** Δ**:**  **1.1*** | ⨁⨁◯◯  Low^a^ | VR distraction may result in better pain-related functioning, compared with conventional exercise. |
| **Pain Intensity or Severity**  VAS | 2 days  N = 55  1 RCT[33] | NC^†^ | NC^†^ | **Diff** Δ**:**  **NC**^†^ | ⨁◯◯◯  Very Low^a,b^ | The evidence is very uncertain on the effect of VR distraction on pain intensity compared with conventional exercise. |
| *Values for mean change in VR physical activity, comparator (conventional therapy) and Diff Δ taken from Fuchs et al[33]  ^†^Median (IQR) values of change in VR physical activity, comparator (conventional therapy) reported in Fuchs et al. Means not reported.  Diff Δ: difference in change scores; No.: number; NRS: numeric rating scale; RCT: randomized controlled trial; VAS: Visual Analogue Scale; VR: virtual reality; WOMAC: Western Ontario and McMaster Universities Arthritis Index | | | | | | |

**Explanations**

a. Downgraded 2 levels for study limitations (study rated high risk of bias)

b. Downgraded one level for indirectness (study reported only medians)

VR-Psychoeducation for Post-TKA

| **Outcome**  Outcome Measure | Follow-up  No. of Participants (Studies) | **Anticipated Absolute Effects** | | | Certainty | What Happens |
| --- | --- | --- | --- | --- | --- | --- |
|  |  | **VR** | **Comparator** | **Difference** |  |  |
| **Pain Intensity or Severity**  VAS | 4 weeks  N = 65  1 RCT[40] | -4.4* | -1.8* | **Diff** Δ**:**  **-2.6*** | ⨁⨁⨁◯  Moderate^a^ | VR psychoeducation probably results in decreased pain intensity compared with conventional exercise. |
| *Values for mean change in VR physical activity, comparator (conventional therapy) and Diff Δ taken from Mazurek et al[40]  CI**:** confidence interval; Diff Δ: difference in change scores; No.: number; NRS: numeric rating scale; RCT: randomized controlled trial; VAS: Visual Analogue Scale; VR: virtual reality; WOMAC: Western Ontario and McMaster Universities Arthritis Index | | | | | | |

**Explanations**

a. Downgraded one level for study limitations (study rated some concerns)

IDR-DAE for post-TKA

| **Outcome**  Outcome Measure | Follow-up  No. of Participants (Studies) | **SMD Pooled Estimate (95% CI)** | **Anticipated Absolute Effects** | | | Certainty | What Happens |
| --- | --- | --- | --- | --- | --- | --- | --- |
|  |  |  | **IDR** | **Comparator** | **Difference** |  |  |
| **Pain-Related Functioning or Interference**  WOMAC total, KOOS, OKS | 3-4 months  N = 473  4 RCTs[31, 35, 47, 48] | - | -36.6* | -35.8* | **Diff** Δ**:**  **-0.8*** | ⨁⨁◯◯  Low^a^ | IDR physical activity may result in better pain-related functioning, compared with conventional exercise. |
| **Pain-Related Functioning or Interference**  WOMAC total | 6 months  N = 50  1 RCT[48] | - | -43.0* | -40.2* | **Diff** Δ**:**  **-2.8*** | ⨁⨁⨁◯  Moderate^b^ | IDR physical activity probably results in better pain-related functioning, compared with conventional exercise. |
| **Pain Intensity or Severity**  VAS, NRS | 2-3 weeks  N = 176  2 RCTs[46, 48] | - | -0.6* | -0.8* | **Diff** Δ**:**  **0.2*** | ⨁◯◯◯  Very Low^a,c^ | IDR physical activity may result in little to no difference in pain intensity compared with conventional exercise. |
| **Pain Intensity or Severity**  VAS, NRS | 3-4 months  N = 376  3 RCTs[35, 47, 48] | **SMD:**  **-0.12 (-0.75, 0.52)** | -2.9* | -2.2* | **Diff** Δ**:**  **-0.7*** | ⨁⨁◯◯  Low^b,d^ | IDR physical activity may result in little to no difference in pain intensity compared with conventional exercise. |
| **Pain Intensity or Severity**  NRS | 6 months  N = 54  1 RCT[48] | - | -3.2* | -3.2* | **Diff** Δ**:**  **0*** | ⨁⨁⨁◯  Moderate^b^ | IDR physical activity probably results in little to no difference in pain intensity compared with conventional exercise. |
| **Adverse Events**  Patient falls | 3 months  N = 139  1 RCT[47] | - | 19.4%^†^ | ^†^14.6% | **Diff** Δ**:**  **4.8%**  **(90% CI -2.60 to 12.25)** ^†^ | ⨁◯◯◯  Very Low^b,e,f^ | The evidence is very uncertain on the effect of IDR physical activity on pain-related functioning compared with conventional exercise. |
| *Values for mean change in VR physical activity, comparator (conventional therapy) and Diff Δ taken from Shim et al[48]  ^†^Values for mean change in VR physical activity, comparator (conventional therapy) and Diff Δ taken from Prvu Bettger et al[47]  CI**:** confidence interval; Diff Δ: difference in change scores; No.: number; NRS: numeric rating scale; RCT: randomized controlled trial; VAS: Visual Analogue Scale; VR: virtual reality; WOMAC: Western Ontario and McMaster Universities Arthritis Index | | | | | | | |

**Explanations**

a. Downgraded 2 levels for study limitations (1 study rated high RoB)

b. Downgraded 1 level for study limitations (studies rated some concerns RoB)

c. Downgraded 1 level for inconsistency (direction of effect varied across studies)

d. Downgraded 1 level for imprecision (confidence interval crosses small effects in both directions)

e. Downgraded 1 level for indirectness (authors only reported falls without assessment of cause, and no other adverse events)

f. Downgraded 1 level for imprecision (confidence interval includes fewer events and substantially more events)

References

31. Eichler S, Salzwedel A, Rabe S, et al. The effectiveness of telerehabilitation as a supplement to rehabilitation in patients after total knee or hip replacement: randomized controlled trial. JMIR Rehabil Assist Technol. Nov 7, 2019;6(2):e14236. [doi: 10.2196/14236] [Medline: 31697239]

33. Fuchs L, Kluska A, Novak D, Kosashvili Y. The influence of early virtual reality intervention on pain, anxiety, and function following primary total knee arthroplasty. Complement Ther Clin Pract. Nov 2022;49:101687. [doi: 10.1016/j.ctcp.2022.101687] [Medline: 36347152]

35. Janhunen M, Katajapuu N, Paloneva J, et al. Effects of a home-based, exergaming intervention on physical function and pain after total knee replacement in older adults: a randomised controlled trial. BMJ Open Sport Exerc Med. 2023;9(1):e001416. [doi: 10.1136/bmjsem-2022-001416] [Medline: 36896366]

37. Jin C, Feng Y, Ni Y, Shan Z. Virtual reality intervention in postoperative rehabilitation after total knee arthroplasty: A prospective and randomized controlled clinical trial. Int J Clin Exp Med. 2018;11(6):6119-6124. URL: www.ijcem.com

40. Mazurek J, Cieślik B, Wrzeciono A, Gajda R, Szczepańska-Gieracha J. Immersive virtual reality therapy is supportive for orthopedic rehabilitation among the elderly: a randomized controlled trial. J Clin Med. Dec 14, 2023;12(24):7681. [doi: 10.3390/jcm12247681] [Medline: 38137750]

43. Nishitha K, Anitha A, Thaheera D. Effectiveness of virtual reality-based rehabilitation and high-intensity exercise program for total knee arthroplasty patients: a randomised controlled trial. J Clin Diagn Res. 2024;18(11). [doi: 10.7860/JCDR/2024/71122.20263]

46. Piqueras M, Marco E, Coll M, et al. Effectiveness of an interactive virtual telerehabilitation system in patients after total knee arthoplasty: a randomized controlled trial. J Rehabil Med. Apr 2013;45(4):392-396. [doi: 10.2340/16501977-1119] [Medline: 23474735]

47. Prvu Bettger J, Green CL, Holmes DN, et al. Effects of virtual exercise rehabilitation in-home therapy compared with traditional care after total knee arthroplasty: VERITAS, a randomized controlled trial. J Bone Joint Surg Am. Jan 15, 2020;102(2):101-109. [doi: 10.2106/JBJS.19.00695] [Medline: 31743238]

48. Shim GY, Kim EH, Lee SJ, et al. Postoperative rehabilitation using a digital healthcare system in patients with total knee arthroplasty: a randomized controlled trial. Arch Orthop Trauma Surg. Oct 2023;143(10):6361-6370. [doi: 10.1007/s00402-023-04894-y] [Medline: 37129691]
