# Supplementary material for: Extended Reality Interventions for Osteoarthritis of the Knee and Recovery After Total Knee Arthroplasty: Systematic Review and Meta-Analyses
Source: J Med Internet Res. 2026 Sep 8;28:e84753. doi: 10.2196/84753 (PMC13555783; doi:10.2196/84753)
Supplement: Supplementary file 6 [file jmir-v28-e84753-s008.docx]

| **Author, Year**  **Risk of Bias**  **Follow-up Duration**  **Site(s)**  **Funding source** | **Inclusion/Exclusion Criteria** | **Intervention:**  **Participants Randomized**  **Demographics**  **Setting**  **Frequency; Duration**  **Detailed Intervention Characteristics** | **Comparator(s):**  **Participants Randomized**  **Demographics**  **Setting**  **Frequency; Duration**  **Detailed Comparator Characteristics** | **Primary Outcome**  **Eligible Outcomes & Measures Reported (timepoints)**  **Other Non-Eligible Outcomes Reported** |
| --- | --- | --- | --- | --- |
| ***VR Intervention*** | | | | |
| Ozlu et al, 2023[45]  High  7 weeks  Turkey  Kutahya Health Sciences University  No funding | **Inclusion**:  Age between 40 and 64 years  American College of Rheumatology concordant diagnosis of stage 2 or 3 knee OA  Minimental status test score of ≥22  **Exclusion:**  Recent or previous operation on the affected lower extremity  History of lower extremity injury including fracture, meniscus and/or ligament tear  History of intra-articular injection to the affected knee in preceding 6 months, knee physical therapy in preceding 6 months  History of rheumatological disease, septic arthritis, cardiovascular disease or other impairment affecting ability to exercise, balance, hearing or vision | N=41  Age, mean (SD): 53.3(10.4)  Female: 48.6%  Clinic  15 mins VR (in addition to time for ultrasound and TENS) per day, 5 days a week (total 15 sessions) 3 weeks  Fish Game: Participants wear Oculus VR glasses and intercept incoming virtual fish using lateral flexion movement of the trunk. Training focused on weight transfer, balance and proprioception of the patients.  Monkey Game: Participants wear Oculus VR glasses and intercept incoming virtual bananas using lateral stepping movements. Training focused on patient depth sense. | N=41  Age, mean (SD): 53.7(9.7)  Female: 68.4%  Clinic  27 mins per day, 5 days/week (total 15 sessions) 3 weeks  Therapeutic ultrasound to affected knee for 7 minutes  Transcutaneous electrical nerve stimulation on both knees for 20 minutes | **Primary outcome NR**  **Pain-related functioning (3, 7 wk)**   - WOMAC   **Pain intensity (3, 7 wk)**   - VAS   **Physical performance (3, 7 wk)**   - 6-min walk - Berg Balance Scale |
| Lo et al, 2024[39]  Some concerns  12 weeks  Hong Kong, China  NR | **Inclusion:**  Diagnosis of KOA based on clinical and radiographic ACR criteria  Moderate to severe knee pain for at least 3 months  **Exclusion:**  Decompensated organic/psychiatric disease  Contraindications to VR (epilepsy, severe myopia >-3.5 diopters)  Comorbidities that may impede exercise | N=15  Age, median (IQR): 63 (60-67)  Female: 67%  Home  30 min/day, 5 days/week; 12 weeks  VRiKnee: Smartphone-based immersive VR app (iOS/Android) with head-mounted display (VR Shinecon 5.0). The app captured outdoor garden scenes to create an immersive exercise environment with ambient audio, a virtual coach, and interactive animations providing visual feedback (virtual flowers blossoming with successful exercise moves). A timer unlocked 3 difficulty levels. Exercises: repeated concentric quadriceps knee-extension and isometric vastus medialis oblique training (squeezing fitness ring between thighs), both in sitting position. | N=15  Age, median (IQR): 64 (62-65)  Female: 87%  Home  30 min/day, 5 days/week; 12 weeks  Same exercises without VR. Participants attended a health talk led by a registered physiotherapist to explain and demonstrate home-based exercises. Two sets of lower limb strengthening exercises selected from the Ottawa Panel Clinical Practice Guideline: repeated knee-extension exercise for concentric quadriceps training and squeezing a fitness ring between thighs for vastus medialis oblique isometric training, both in sitting position. | **Primary outcome NR**  **Pain-related functioning (6, 12 wk)**   - WOMAC total, pain, stiffness, function   **Pain intensity (6, 12 wk)**   - NRS   **Quality of life (6, 12 wk)**   - EQ-VAS   **Adverse events (12 wk)**  **Adherence (12 wk)** |
| **IDR Interventions** | | | | |
| Elshazly et al, 2016[32]  Some concerns  8 weeks  Saudi Arabia  Prince Sattam Bin Abdul Aziz University  Grant from deanship of scientific research, Prince Sattam Bin Abdul Aziz University, Saudi Arabia | **Inclusion**:  Male and Female  Age between 35 and 65 years  ≥ 3 months of symptomatic osteoarthrits  Ambulate at least 30 feet with or without assistance  Not in any sports or physical therapy  Able to complete physical therapy three times weekly  **Exclusion:**  Acute medical illness or knee surgery within 6 months of enrollment  History of metal implants, peripheral vascular disease, infection, fever, mental deficit, femur or tibial condyle fracture, joint effusion, laboratory abnormality, systemic or psychiatric illness, corticosteroid use in the preceding 30 days, malignancy, gout or any other disease preventing participation | AR Physical Activity:  N=20  Age, mean (SD): 58(6)  NR  NR  15-30 minute sessions, 3 times weekly 8 weeks  Light Race: Participants use Xbox 360 Kinect to step on a virtual platform displayed on a monitor. On-screen commands have participants step forward, backward, right and left in both sitting and standing positions for 10 repetitions with a two-minute rest between exercises. Exercises focused on lower limb strength, flexibility, coordination, and balance. Sessions were held in small groups of three or less subjects | Sensorimotor Training (SMT):  N=20  Age, mean (SD):  60(8)  NR  NR  3 times weekly; 8 weeks  Participants were trained through three stages:  Static [ie, standing upright position (30 s), single leg stance with closed eyes, half-step position for 10 s, one-leg balance for 10 s]  Dynamic [ie, forward stepping thrust, T-band kicks exercise],  Functional [ie, 1) walking exercise on a firm surface, then on a foam surface: (a) toe skipping, (b) heel skipping; 2) squatting exercise: (a) against a wall and away from the wall, (b) one leg squats on affected and non-affected limbs; 3) balance exercise on wobble board: (a) multidirectional rolling movement from sitting, (b) multidirectional rolling movement from standing on both legs between parallel bars with eyes open, then eyes closed, (c) multidirectional rolling movement from standing on one leg between parallel bars with eyes open, then eyes closed].” | **Primary outcome NR**  **Pain-related functioning (4, 8 wk)**   - WOMAC   **Pain intensity**  **(4, 8 wk)**   - VAS   **Physical performance (4, 8 wk)**   - Position sense   **Quality of life (4, 8 wk)**   - CDC Health Related Quality of Life |
|  |  |  | Conventional Exercise Therapy (CET):  N=20  Age, mean (SD): 59(7)  NR  NR  3 times weekly; 8 weeks  5 minutes warm up followed by 12 minutes of walking at comfortable pace and 5 minutes cool down. |  |
| Lin et al, 2020[38]  Some concerns  3 months  Taiwan  Shin Kong Wu Ho-Su Memorial Hospital  Grants from Shin Kong Wu Ho-Su Memorial Hospital and Ministry of Science and Technology, Taiwan | **Inclusion**:  Age 40 to 85 years  American College of Rheumatology concordant diagnosis of knee OA  Kellgren and Lawrence score ≥2  Able to walk >15 m  Able to undergo 4 weeks of treatment and 3 months of follow-up  Not needing non-steroidal anti-inflammatory drugs during the research  **Exclusion:**  Any infection, inflammation, autoimmune disease, or fracture  History or underlying disease affecting posture and balance, such as malignancy, dizziness, vertigo, or stroke  History of any knee operation or internal fixation  Pregnancy or planning to become pregnant  Undergoing any other treatment for knee OA." | N=40  Age, mean (SD): 55.9(15.8)  Female: 60%  Clinic  3 times per week; 4 weeks  Hot packs applied to both knees for 20 min, transcutaneous electric nerve stimulation (TENS) for 20 min, 20 min of active video games or therapeutic exercise  Whack-a-mole: Participants use Hot Plus gaming system and a step sensing pad. Participants step on step sensing pad to stamp on-screen moles into holes. Training focused on fast movement and improving lower limb range of motion, strength and coordination. Activity lasts 10 minutes.  Archery: Participants use Hot Plus gaming system and a step sensing pad. Participant steps on sensing pad to shoot arrows at on-screen targets. Training focused on fast movement and improving lower limb range of motion, strength, and coordination. Activity last 10 minutes. | N=40  Age, mean (SD): 58.1(16.9)  Female: 42.5%  Clinic  3 times per week; 4 weeks  Hot packs applied to both knees for 20 min, transcutaneous electric nerve stimulation (TENS) for 20 min, 20 min of active video games or therapeutic exercise  Therapeutic exercise includes stretching warm-up, stabilization exercises, weight-shift training for knees and pelvis … cycling for 10 min with approximately 40% to 60% heart rate reserve, and stretching cool-down. Sandbag and Thera-band were used in strength exercises with a maximum of 10 repetitions and 5 to 10 repetitions per session, for 3 to 5 sets. Activity lasts for a total of 20 min and supervised by physical therapists. | **Primary: WOMAC (only separate domain scores for pain, stiffness, & function)**  **Pain intensity (2, 4, 8, 16 wk)**   - Chronic Pain Grade Questionnaire- overall grade; disability points; disability score; pain intensity   **Adverse events (4 wk)**   - Non-specific adverse events   **Physical performance** **(2, 4, 8, 16 wk)**   - Biodex Stability System, Postural Stability & Limits of Stability, - 10 m walk - Stair ascent, descent times   **Quality of life (2, 4, 8, 16 wk)**   - WHOQOL-BREF (domain scores for physical, psychological, social, & environmental)   **Other non-eligible outcomes reported**   - HADS - Multidimensional Fatigue Inventory - Work Ability Index |
| Mete et al, 2022[41]  High  6 Weeks  Turkey  Physical therapy and rehabilitation clinic of medical center (name NR) in Istanbul  Marmara University Scientific Research Projects Committee (Project Number: SAG‐C‐YLP‐070617‐0349) | **Inclusion**:  Aged 40 to 65 years  Diagnosis of knee OA  Kellgren Lawrence stage 2 or 3  **Exclusion:**  History of surgery at knee, hip, ankle, or/and foot, systemic anti-inflammatory joint disease, any condition with contraindication for electrical stimulation and/or exercise, previous knee physiotherapy in preceding 6 months and a history of corticosteroid injection in preceding 3 months | N=32  Age, mean (SD): NR  Median age: 59.5 (55,64)  Female: 80%  Clinic  20 games for 5 days per week; 6 weeks  Crazy Wings: Participant controls an on-screen bird avatar through obstacles using knee movement. Sensors from the MarVAJED system were placed above and below knee, which evaluate the ROM of the joints, analyze the sensation of joint position, and allow control of on-screen avatars. Knee flexion and extension triggers bird avatar movement up and down, respectively. Training aimed to have participants complete squat exercises.  Blasting Ball: Participant controls the size of a virtual ball size on a screen through knee flexion. Sensors from the MarVAJED system were placed above and below knee, which evaluate the ROM of the joints, analyze the sensation of joint position, and allow control of on-screen avatars. The ball would explode when the degree of knee flexion reached a peak value. Training aimed to have participants complete leg press exercises and increase degree of knee flexion. | N=32  Age, mean (SD): NR  Median age: 57 (51, 65)  Female: 76.6%  Clinic  5 days per week; 6 weeks  Electrotherapy, exercise program, hot and cold pack therapy, therapeutic ultrasound, and conventional transcutaneous electrical nerve stimulation were completed. Participants with edema were treated with a cold pack, and participants without edema received hot pack. Exercise program consists of isometric strengthening of the quadriceps muscle, terminal knee extension, leg press exercise with elastic band, and hamstring stretching. Elastic band training involved 3 sets of 10 repetitions. | **Primary outcome NR**  **Pain related functioning or interference (6 wk)**   - WOMAC-stiffness - WOMAC-function   **Pain intensity (6 wk)**   - WOMAC-pain - VAS   **Pain Catastrophizing (6 wk)**   - TSK   **Physical performance (6 wk)**   - PEDALO Sensamove balance score - Knee flexion/extension ROM - Knee proprioception at 30, 60 degrees - Peak torque of knee flexion/extension (absolute and normalized to body weight) at 120°, 240° |
| Nambi et al, 2020[42]  Some concerns  3 months  Saudi Arabia  Prince Sattam Bin Abdulaziz University Hospital and King Khalid Hospital, Al-Kharj, Saudi Arabia  Deanship of Scientific Research at Princess Nourah bint Abdulrahman University through the Fast-track Research Funding Program | **Inclusion:**  Male gender  Age 18 to 25 years  ≥3 months post-traumatic osteoarthritis following ACL injury as diagnosed by an orthopedic surgeon  Pain rating 4 to 8 in visual analog scale (VAS)  **Exclusion:**  Other orthopedic, neural, systemic, psychological diagnosis  Any pending surgical procedure  Participation in any other treatment and physical training | N=20  Age, mean (SD): 22.8(1.3)  Female: 0%  Clinic  2 20-minute sessions per day, 5 days a week; 4 weeks  Participants used the Pro-Kin system PK 252 N Techno body. Training involved standing on the affected limb and following commands on a computer display screen to shoot virtual balls. Interaction with balls was controlled by having the participant follow specified knee movements. | SMT:  N=20  Age, mean (SD): 22.6(1.4)  Female: 0%  Clinic  5 days per week; 4 weeks  Sensorimotor training exercises in three consecutive stages: Static phase where the participant stands straight for 30 seconds on a hard plate and 30 seconds on a foam plate. Next, the participant was instructed to stand on a single leg (affected side) with closed eyes for 20 seconds on a hard plate and 20 seconds on a foam plate, followed by a semi knee bending position for 10 seconds. Dynamic phase where the participant performs forward kicking for 30 seconds and T-band kicking for 30 seconds. Functional phase where the participant was informed to do toe jumping for 20 meters and heel jumping for 20 meters. Then the participant performs bilateral and unilateral squatting exercises for 10 repetitions with and without the support of a wall. All exercises were continued for 3 sets each with 5 repetitions and 3 minutes rest between the sets. | **Primary outcome NR**  **Pain related functioning or interference (4, 8 wk, 3 mo)**   - WOMAC-total   **Pain intensity (4, 8 wk, 3 mo)**   - VAS   **Other non-eligible outcomes reported**   - Bone morphogenic protein levels (BMP 2,4,5 and 7) - Inflammatory biomarkers serum levels (CRP, TNF-alpha, IL2, IL4 and IL6) |
|  |  |  | Control, Supervised Exercise:  N=20  Age, mean (SD): 21.9(1.3)  Female 0%  Clinic or health care facility  5 days per week; 4 weeks  Control group completed supervised conventional exercise programs for the knee muscles. Exercises laid special stress on the quadriceps, hamstrings, glutei, and calf muscles. The participants performed 10–15 repetitions in one set for 3 sets with 1 minute rest between the sets. Then participants completed stretching focused on each muscle group for 3 repetitions for 15 seconds per muscle group" |  |
| Jarungvittayakon et al, 2025[36]  Some concerns  6 weeks  Thailand  NR  NR | **Inclusion:**  Primary KOA per ACR criteria, Kellgren-Lawrence stage 2-3  Age 40-75 years  **Exclusion:**  Previous significant injury or surgery  Intra-articular injection within 6 months  Neuromuscular disease | N=28  Age, mean (SD): 60 (5.8)  Female: 82%  Home  Daily, twice a day; 6 weeks  Exergaming with wearable IMU sensors and mobile game app (iOS/Android). Two sensor boxes attached above and below the knee measured knee flexion-extension angle. Airplane Game: participants performed deep knee flexion and full extension to navigate an on-screen airplane past obstacles. Five repetitions of deep knee bending and full stretching, maintaining extension for 10 seconds each. | N=28  Age, mean (SD): 62.1 (5.6)  Female: 79%  Home  Daily, twice a day; 6 weeks  Standard conservative treatment only: quadriceps strengthening exercise (5 reps deep knee flexion and full extension, twice daily), concomitant KOA medications, weight reduction and self-care advice. | **Primary: Quadriceps strengthening evaluation**  **Pain-related functioning (6 wk)**   - KOOS   **Pain intensity (6 wk)**   - VAS-Rest, VAS-Motion   **Physical performance (6 wk)**   - ROM Flexion, Extension, - Arc of motion - TUG |
| Oliveira et al, 2024[44]  High  8 weeks  Brazil  NR  NR | **Inclusion:**  KOA with Kellgren-Lawrence grades 2, 3, or 4  Independent ambulation ability  **Exclusion:**  Severe hearing loss/visual deficit  Previous orthopedic surgeries  Neurological diseases  Loss of protective foot sensitivity | N=20  Age, mean (SD): 62.4 (7.4)  Female: 70%  NR  Twice weekly; 8 weeks (50-min sessions)  Non-immersive VR using Xbox 360 with Kinect 360 sensor on 42-inch TV. Kinect Adventures! with three standardized subgames: (1) Reflex Ridge—obstacle avoidance with squats and jumps; (2) 20000 Leaks—reaching targets requiring weight transfer and unipodal support; (3) RallyBall—quick movements to throw/intercept balls. Each game 6 min with 1-min breaks (20 min VR total).  **Co-intervention (both groups):**  30 min kinesiotherapy: quadriceps/hamstring stretching and strengthening with shin guards, sit-to-stand, bipodal balance on disk (3 sets of 10 reps). | N=20  Age, mean (SD): 62.6 (8.6)  Female: 85%  NR  Twice weekly; 8 weeks (50-min sessions)  Conventional exercises for 20 min: (1) forward/backward/sideways displacements and squats using four colored cones in a square 2m apart, with therapist verbal commands; (2) ball game throwing/receiving with upper and lower limbs alternately. Two 3-min repetitions per exercise with 2-min rest. | **Primary outcome NR**  **Pain-related functioning (8 wk)**   - WOMAC total   **Pain intensity (8 wk)**   - VAS   **Physical performance (8 wk)**   - Anticipatory postural adjustments |
| Carvajal-Parodi et al, 2024[29, 34]  Low  14 weeks  Chile  NR  NR | **Inclusion:**  Age 60-84 years  Mild-to-moderate KOA/HOA per ACR criteria, Kellgren-Lawrence grade 2-3  Ability to walk independently for at least 6 minutes  **Exclusion:**  Decompensated health conditions  Inability to properly use exergames  Use of opioids or interfering medications  MMSE score < 18 | N=30  Age, mean (SD): 68.7 (5.5)  Female: NR  Clinic  3x/week; 10 weeks (30 min conv. exercise + 20 min exergames)  Exergaming using Nintendo Switch on a 43-inch television. Specific exercises, yoga postures, and interactive challenges performed following a virtual guide. Games selected to target strength, aerobic endurance, flexibility, balance, or motor coordination. Intensity individualized as light-to-moderate using perceived exertion scale (0-10). | N=30  Age, mean (SD): 69.0 (5.6)  Female: NR  Clinic  3x/week; 10 weeks (50 min conv. exercise)  Conventional therapy: TENS and hot packs (10 min), 5-min warm-up, 50 min exercise (stationary cycling, step-ups, treadmill; strengthening with elastic bands/dumbbells; balance training on unstable surfaces; flexibility exercises), 5-min cool-down, then 10 min TENS and hot-pack. | **Primary outcome NR**  **Pain intensity (14 wk)**   - VAS   **Physical performance (14 wk)**   - TUG   **Adverse events (14 wk)**  **Adherence (14 wk)** |
| Dal Erdogan et al, 2025[30]  High  3 weeks  Turkey  NR  NR | **Inclusion:**  Kellgren-Lawrence stage 2-3 KOA  Age 40-75 years  **Exclusion:**  Cognitive dysfunction  Inability to stand without support  Visual/auditory disorders  Knee surgery or injection in last 6 months | **3-arm study:**  **Arm 2 (Exercise + VR):**  N=18  Age, mean (SD): 60.1 (6.5)  Female: 60.8 (6.5%)  Clinic  5 days/week; 3 weeks (15 sessions)  40-min conventional exercise followed by 20-min VR using BTS Nirvana system (BTS, Italy)—screen-projected VR with optoelectronic infrared sensors. System generates exercises targeting trunk, lower/upper extremities, and cognitive processes.  **Arm 3 (VR only):**  N=18  Age, mean (SD): 60.1 (5.6)  Female: 78%  Clinic  5 days/week; 3 weeks (15 sessions)  20-min VR session using BTS Nirvana only, without conventional exercise. | **Arm 1 (Exercise only):**  N=18  Age, mean (SD): 61.1 (6.2)  Female: 78%  Clinic  5 days/week; 3 weeks (15 sessions)  40-min conventional exercise therapy: strengthening, stretching, and ROM exercises targeting lower extremities (quadriceps, hamstrings, gastrocnemius, hip abductors). Strengthening: 3 sets of 10 reps with body weight and resistance bands, 30-sec rest. Stretching: hamstrings, calves, hip flexors held 20-30 sec. Active and passive ROM for knee flexion/extension. | **Primary outcome NR**  **Pain-related functioning (3 wk)**   - WOMAC total, pain, stiffness, function   **Pain intensity (3 wk)**   - VAS-Rest, VAS-Movement |
| Buyukyilmaz et al, 2024[28]  High  8 weeks  Turkey  NR  NR | **Inclusion:**  OA-induced knee pain for minimum 6 months  Primary OA per ACR criteria, Kellgren-Lawrence grade 2-3  **Exclusion:**  Secondary OA  Previous knee/hip/spine surgery  Physiotherapy for serious knee trauma within past year | N=28  Age, mean (SD): 58.9 (3.6)  Female: NR  Clinic  30 min, 5 days/week; 3 weeks (assessed at 8 wk)  Wii Balance Board-based exergaming using Becure Balance System (Becure Global, Germany). Three games: (1) Balance Surf—mediolateral stability; (2) Balance Adventure—multi-dimensional weight transference; (3) Balance Bowling—static and dynamic postural control. Games played under physiotherapist guidance, difficulty constant throughout.  **Co-intervention (both groups):**  15 sessions electrotherapy/modalities: 20-min hot pack, ultrasound (1.5 W/cm², 1 MHz, 5 min/knee), conventional TENS (50-150 Hz, 20 min). | N=28  Age, mean (SD): 60.3 (3.7)  Female: NR  Clinic  30 min, 5 days/week; 3 weeks (assessed at 8 wk)  Structured closed kinetic chain exercises (CKCE) for 30 min under physiotherapist supervision. Exercises integrated from easy to difficult each week, with 10-sec rest between exercises. | **Primary outcome NR**  **Pain-related functioning (3, 8 wk)**   - WOMAC total   **Physical performance (3, 8 wk)**   - ROM Flexion - Knee flexion/extension strength - Joint position sense (15, 45, 75 degrees) - TUG |
| Sonmez et al, 2025[49]  Some concerns  3 weeks  Turkey  NR  NR | **Inclusion:**  Age over 18 years  Kellgren-Lawrence stage 1-3  Ability to walk >15m independently  **Exclusion:**  Infection, autoimmune disease, fracture  Dizziness, vertigo, stroke  Neurological/vestibular diseases  Previous knee surgery or injection in last 6 months | N=20  Age, mean (SD): 60.1 (8.3)  Female: 75%  Clinic  5 days/week; 3 weeks (VR 30 min + trad PT 45 min)  Non-immersive VR using Microsoft Kinect Xbox 360. River Rush—Curvy Creek game: participants stood in a boat on a flowing river, overcoming obstacles by jumping, crouching, and stepping left/right. Training targeted lower limb proprioception, balance, and coordination.  **Co-intervention (both groups):**  Traditional PT: hot pack (20 min), TENS (100 Hz, 20-35 mA, 20 min), 1 set/10 reps knee flexion/extension in sitting. | N=20  Age, mean (SD): 64 (8.8)  Female: 75%  Clinic  5 days/week; 3 weeks (sham VR 30 min + trad PT 45 min)  Sham VR: participants sat on a chair in front of screen with lower extremities fixed by belt. Played Rally Ball Game using upper extremities only, blocking oncoming balls in random directions under physiotherapist supervision. Designed to control for screen/game exposure without lower extremity VR interaction. | **Primary outcome NR**  **Pain-related functioning (3 wk)**   - WOMAC function, WOMAC pain   **Physical performance (3 wk)**   - TUG - Proprioception (right, left)   **Adverse events (3 wk)** |

ACL=anterior cruciate ligament; ACR=American College of Rheumatology; AR=augmented reality; CDC=Centers for Disease Control and Prevention; CKCE=closed kinetic chain exercise; IDR=interactive digital rehabilitation; IQR=interquartile range; KOOS=Knee Injury and Osteoarthritis Outcome Score; min=minutes; MMSE=Mini-Mental State Examination; N=number of participants; NR=not reported; NRS=numeric rating scale; OA=osteoarthritis; PT=physical therapy; ROM=range of motion; SD=standard deviation; TENS=transcutaneous electrical nerve stimulation; TSK=Tampa Scale for Kinesiophobia; TUG=Timed Up and Go test; VAS=Visual Analog Scale; VR=virtual reality; WHOQOL-BREF=World Health Organization Quality of Life Brief Version; WOMAC=Western Ontario and McMaster Universities Osteoarthritis Index

References

28. Büyükyilmaz G, Menek B, Tarakçi D. Closed kinetic chain exercises therapy versus Wii-based Exergame therapy in the treatment of knee osteoarthritis: randomized controlled trial. Arch Health Sci Res. 2024;11(2):133-139. [doi: 10.5152/ArcHealthSciRes.2024.23139]

29. Carvajal-Parodi C, Mendoza C, Alvarez C, et al. Effectiveness of exergames on functional physical performance in older adults with knee/hip osteoarthritis: a randomized controlled trial. J Clin Med. Apr 25, 2025;14(9):2968. [doi: 10.3390/jcm14092968] [Medline: 40363999]

30. Dal Erdoğan S, Berkan F, Armağan O, Özgen M, Çiracioğlu AM, Şahi̇n Mutlu F. The effects of virtual reality-based exercise on pain and function in older adults with knee osteoarthritis: a randomized controlled study. Turk J Geriatr. 2025;28(2). [doi: 10.29400/tjgeri.2025.432]

[32. Elshazly FAA, Nambi GS, Elnegamy TE. Comparative study on virtual reality training (VRT) over sensory motor training (SMT) in unilateral chronic osteoarthritis—A randomized control trial. Int J Med Res Health Sci. 2016;5(8):7-16. URL: https://www.ijmrhs.com/](https://www.ijmrhs.com/)

34. Guede-Rojas F, Mendoza C, Rodríguez-Lagos L, et al. Effects of non-immersive virtual reality exercise on self-reported pain and mechanical hyperalgesia in older adults with knee and hip osteoarthritis: a secondary analysis of a randomized controlled trial. Medicina (B Aires). 2025;61(7):1122. [doi: 10.3390/medicina61071122]

36. Jarungvittayakon C, Sa-Ngasoongsong P, Chockchaisakul D, Bamrungchaowkasem J, Wongsak S. Effectiveness of mobile exergaming with sensor-based visual feedback as an adjunct therapy for home-based quadriceps exercise training in knee osteoarthritis: a prospective randomized controlled trial. Life (Basel). Nov 12, 2025;15(11):1738. [doi: 10.3390/life15111738] [Medline: 41302162]

38. Lin YT, Lee WC, Hsieh RL. Active video games for knee osteoarthritis improve mobility but not WOMAC score: a randomized controlled trial. Ann Phys Rehabil Med. Nov 2020;63(6):458-465. [doi: 10.1016/j.rehab.2019.11.008] [Medline: 31981832]

39. Lo HHM, Ng M, Fong PYH, et al. Examining the feasibility, acceptability, and preliminary efficacy of an immersive virtual reality-assisted lower limb strength training for knee osteoarthritis: mixed methods pilot randomized controlled trial. JMIR Serious Games. Sep 27, 2024;12:e52563. [doi: 10.2196/52563] [Medline: 39331525]

41. Mete E, Sari Z. The efficacy of exergaming in patients with knee osteoarthritis: a randomized controlled clinical trial. Physiother Res Int. Jul 2022;27(3):e1952. [doi: 10.1002/pri.1952] [Medline: 35470534]

42. Nambi G, Abdelbasset WK, Elsayed SH, Khalil MA, Alrawaili SM, Alsubaie SF. Comparative effects of virtual reality training and sensory motor training on bone morphogenic proteins and inflammatory biomarkers in post-traumatic osteoarthritis. Sci Rep. Sep 28, 2020;10(1):15864. [doi: 10.1038/s41598-020-72587-2] [Medline: 32985509]

44. Oliveira LKR, Marques AP, Andrade KFA, et al. Virtual reality in improving anticipatory postural adjustments to step initiation in individuals with knee osteoarthritis: a randomized controlled trial. Games Health J. Apr 2024;13(2):100-108. [doi: 10.1089/g4h.2023.0154] [Medline: 38436585]

45. Özlü A, Ünver G, Tuna Hİ, Menekşeoğlu AK. The effect of a virtual reality-mediated gamified rehabilitation program on pain, disability, function, and balance in knee osteoarthritis: a prospective randomized controlled study. Games Health J. Apr 2023;12(2):118-124. [doi: 10.1089/g4h.2022.0130] [Medline: 36603100]

49. Sönmez M, Avcı Ş, Şimşek F, Baygutalp F. Nonimmersive virtual reality-based exercises improve muscle excitability and balance in patients with knee osteoarthritis: a sham-controlled study. Games Health J. Dec 2025;14(6):493-503. [doi: 10.1177/2161783X251360443] [Medline: 40711924]
