# Supplementary material for: Extended Reality Interventions for Osteoarthritis of the Knee and Recovery After Total Knee Arthroplasty: Systematic Review and Meta-Analyses
Source: J Med Internet Res. 2026 Sep 8;28:e84753. doi: 10.2196/84753 (PMC13555783; doi:10.2196/84753)
Supplement: Supplementary file 7 [file jmir-v28-e84753-s009.docx]

| **Author, Year**  **Risk of Bias**  **Follow-up Duration**  **Country**  **Site(s)**  **Funding source** | **Inclusion/Exclusion Criteria** | **Intervention:**  **Participants Randomized**  **Demographics**  **Setting**  **Frequency; Duration**  **Detailed Intervention Characteristics** | **Comparator:**  **Participants Randomized**  **Demographics**  **Setting**  **Frequency; Duration**  **Detailed Comparator Characteristics** | **Primary Outcome**  **Eligible Outcomes & Measures Reported (timepoints)**  **Other non-eligible Outcomes Reported** |
| --- | --- | --- | --- | --- |
| ***VR Interventions*** | | | | |
| Fuchs et al, 2022[33]  High  6 months  Kaplan Medical Center, Rehovot  "This research did not receive any specific grant from funding agencies in the public, commercial or not-for-profit sectors." | **Inclusion**: "(1) Patients diagnosed with osteoarthritis, either uni/double/triple compartmental osteoarthritis, and found suitable for surgery. All grades of osteoarthritis were included in the study; (2) Patients undergoing unilateral TKA."  **Exclusion**: “(1) Patients undergoing revision of TKA; (2) Ligament injury or periprosthetic fracture occurring during TKA; (3) Unstable vital signs; (4) Vision loss, hearing loss, or functional illiteracy." | N=30  Age, mean (SD):  70 (7)  Female: 63.3%  Clinic/facility  1 session per day; 1-2 days post-operatively  "The VR intervention included a movie that was picked by the patient from several options, either a nature film or a music film. The patient watched the VR film during the CPM physiotherapy." | N=25  Age, mean (SD):  70 (7)  Female: 52.0%  Clinic/facility  1 session per day; 1-2 days post-operatively  "The CPM was used as a tool to match the physiotherapy conditions for all patients and to measure a possible change in the range of motion." | **Primary: VAS**  **Pain related functioning or interference (6 mo)**   - Modified KOOS |
| Jin et al, 2018[37]  High  6 months  Cangzhou Central Hospital  NR | **Inclusion**:  "1) Patients diagnosed as OA according to The Guidelines for Diagnosis and Treatment of Osteoarthritis issued by the Chinese Rheumatology Association in 2010; 2) Patients undergoing unilateral TKA for the first time..."  **Exclusion**:  "1) Overweight (BMI ≥ 30 kg/m^2); 2) Severe osteoporosis; 3) Ligament injury or periprosthetic fracture occurring during TKA; 3) Unstable vital signs, complications of incision healing, or clot formation in leg veins; 4) Vision loss, hearing loss, or functional illiteracy. | N=33  Age, mean (SD):  66.45 (3.49)  Female: 54.55%  Clinic/facility  30 minutes, 3 times per day; NR  "… patients exercised by performing foot dorsiflexion and plantar flexion beginning the first day after TKA. Exercises targeting quadriceps muscle strength occurred from the second day after TKA. Passive exercises on knee flexion began after the drainage tube was removed. Exercises were assisted with psychological intervention and pain management education... In addition, VR (Mide Technology Inc., Cangzhou, China) intervention was applied in the experimental group beginning the second day after TKA. Patients were asked to row a boat using knee flexion (interaction of VR) in an immersive virtual environment…” | N=33  Age, mean (SD):  66.30 (4.41)  Female: 60.61%  Clinic/facility  30 repetitions, 3 times per day; NR  ",,,patients exercised by performing foot dorsiflexion and plantar flexion beginning the first day after TKA. Exercises targeting quadriceps muscle strength occurred from the second day after TKA. Passive exercises on knee flexion began after the drainage tube was removed. Exercises were assisted with psychological intervention and pain management education...Patients in the control group were asked to flex their knees passively using their arms until pain tolerance was reached. They held that position for 20 seconds followed by relaxing for 40 seconds." | **Primary: WOMAC, VAS, ROM**  **Other non-eligible outcomes reported (1, 3, 6 mo)**  HSS |
| Mazurek et al, 2023[40]  NR  4 weeks  Poland  NR | **Inclusion:**  Age 60 years or older  Recent hip or knee joint arthroplasty  **Exclusion:**  Cognitive impairments  History of bipolar affective disorder or other psychiatric conditions  Consciousness disorders | N=34  Age, mean (SD): 69.7 (6.8)  Female: 61.8  NR  20 min/session, 2x/week; 4 weeks (8 sessions)  VRTierOne device (Stolgraf, Poland) with HTC VIVE VR goggles and two controllers. Immersive VR psychotherapy based on Ericksonian psychotherapy principles. Sessions began at a virtual garden door; the participant entered an evolving garden that became more vibrant each session. Midway, participants colored a mandala using controllers. Therapy combined metaphorical communication, post-hypnotic therapeutic suggestions, calming music, cognitive engagement through mandala coloring, and a mood-enhancing green garden aesthetic rooted in Japanese design. | N=32  Age, mean (SD): 69.5 (5.5)  Female: 61.8  NR  4 weeks  Conventional rehabilitation only (same co-intervention received by both groups). | **Primary outcome NR**  **Pain-related functioning (4 wk)**  Barthel Index  **Pain intensity (4 wk)**  VAS  **Physical performance (4 wk)**  Tinetti  Rivermead Motor Assessment Gross-Function |
| Nishitha et al, 2024[43]  NR  12 weeks  India  NR  NR | **Inclusion:**  Men and women aged 45 to 65 years  Unilateral TKA  **Exclusion:**  Unstable disease  Previous orthopedic pathologies on same side  Cataract surgery/vision loss  Hearing loss  Parkinson’s, vertigo, cardiorespiratory conditions | N=18  Age, mean (SD): NR  Female: NR  Clinic  4 days/week, 40 min/day; 12 weeks  Week 1: Immersive VR rowing boat game using sensors on limbs and 3D head-mounted glasses. Participants used knee flexion to paddle a boat in a virtual world (30 min, every 3 days with 7-min intervals between 10-min sets).  Weeks 1–12: Non-immersive VR games via computer. Players manipulated an avatar through knee flexion, standing, squatting, and weight shifting. Participants also performed conventional exercises including lateral weight shift exercises concurrently. | N=18  Age, mean (SD): NR  Female: NR  NR  4 days/week; 12 weeks  High-intensity graded exercise program:  Weeks 0–2: Heel glides, short arc knee extensions, bilateral squats, hip external rotation (clams), ankle pumps. Progress when ROM >15°–80° and NPRS at rest <5/10.  Weeks 2–4: Added bilateral calf raises, hip abduction, straight leg raise, sit-to-stand transfers, forward lunging, single-leg standing, multi-directional stepping.  Weeks 4–6: Added single-leg knee extension/flexion, calf press, standing hip exercises, wall slides to 90°, step-downs, side step-ups, tilt board squats, stability ball hip extension.  Weeks 6–12: Added eccentric knee flexion/extension, single-leg press, eccentric calf press, multi-directional lunging, endurance wall slides (5–10 sec holds at 90°). | **Primary outcome NR**  **Pain-related functioning (9, 12 wk)**  WOMAC total  **Pain intensity (4, 12 wk)**  NRS  **Physical performance (4, 12 wk)**  ROM Flexion |
| ***IDR Interventions*** | | | | |
| Eichler et al, 2019[31]  High  3 months  3 Sites (unspecified)  German Pension Insurance Berlin-Brandenburg (grant number 10-40.07.05.07.007); Deutsche Forschungsgemeinschaft; and Open Access Publishing Fund of University of Potsdam | **Inclusion**: "[T]otal hip or knee replacement was performed following idiopathic, posttraumatic, or congenital osteoarthritis, between 18-65 years, and... additional criteria (eg, High Definition Multimedia Interface [HDMI]-compatible screen, minimum 2.5-meter space in front  of the screen, and internet access) …"  **Exclusion**: "Patients not expected to achieve functional safety in walking with full load by the end of the rehabilitation were excluded. For those patients, it was assumed that they would not be able to perform exercises with adequate load or the assessments at the study site. Insufficient verbal and written German-language skills also led to exclusion." | N=56  Age, mean (SD):  53.5 (7.0)  Female: 54.2%  Home  3 x per week; 3 months (following 3 weeks of inpatient rehabilitation)  "... home-based telerehabilitation program based on the MeineReha system, which consisted of a home component as well as a working portal for the therapist in the clinic...The exercises to build up strength and improve postural control were chosen by the supervising therapist …The training intensity was individualized in terms of the choice of exercises, the number of sets and repetitions, and the duration of the breaks, which could all be adjusted by the therapist... options for the patient and the therapist to communicate …: (1) the patient could record and send audio messages to their therapist...and the therapist was able to listen to it whenever their schedule gave them time to do it; (2) the therapist could respond or start a conversation...with individualized text messages, which the patient was shown whenever they started the system...; and (3) the patient and the therapist were able to make appointments for live video conferences, which they were supposed to conduct on a weekly basis... During the training, the exercises were demonstrated on screen by an avatar... The patient performed the exercises simultaneously and was detected by means of a Kinect sensor…The system…sent them real-time visual feedback in which their relevant body segments were colored green for correct movements and red in the case of incorrect movements … For training supervision, the therapist was given access to the frequency of the training as well as the exercise evaluations." | N=55  Age, mean (SD):  56.8 (5.7)  Female: 48.7%  Home  NR; 3 weeks inpatient followed by no specific therapy  "Patients in the control group did not receive any study-specific therapy after their inpatient rehabilitation. The follow-up was carried out identically to the [intervention group] three months after randomization. The patients of both groups were also offered the usual aftercare..." | **Primary: 6-minute walk test**  **Pain related functioning or interference (3 mo)**   - WOMAC - Five Times Chair Rise Test   **Physical performance (3 mo)**   - TUG - Stair Ascend Test   **Quality of life (3 mo)**   - SF-36-physical - SF-36-mental |
| Janhunen et al, 2023[35]  Some Concerns  4 months  Turku University of Applied Sciences, University of Jyväskylä  Päivikki and Sakari Sohlberg Foundation; the Business Finland (grant numbers: 5794/31/2016, 5941/31/2016, 6057/31/2016); Finnish partner companies (SE Innovations Oy [Senior Some Oy], Suunto Oy, Physiotools Oy, GoodLife Technology Oy, Lingsoft Oy, eSeteli Palveluverkko Oy, PN Turku Oy, Ade Animations Design & Effects Oy, Adesante Oy, 4FeetUnder, Intechso and Realmax Oy) | **Inclusion**: “(1) first primary unilateral TKR, (2) mechanical axis of the limb in varus, (3) posterior stabilising or cruciate-retaining prosthesis and (4) normal vision with or without eyeglasses"  **Exclusion**: “[F]ractures, rheumatoid arthritis or other biomechanical disruptions in the affected lower limb within 1 year before surgery, a diagnosed memory disorder, cognitive impairment or a neurological condition." | N=25  Age, mean (SD):  66.9 (3.1)  Women: 64.0%  Home  4-5 exergames (variable duration and sets) played several times a day; 16 wks  "…[P]rotocol included 11 games…The player controls the games using movements similar to the standard postoperative home exercise program…Exergames were categorized according to exercise target: knee extension and flexion, knee flexion or squatting, weight shifting from side to side, stretching, and functional exergames…The knee extension-flexion games (the Cave Game and Intruders) and stretching game (the Cannon) were played in a sitting position, whereas the other games (the Rowing Game, Pick Up, Squat Pong, Bubble Runner, Hat Trick, Brick Breaker, Hiking, and Toy Golf) were played in a standing position." | N=27  Age, mean (SD):  66.4 (4.5)  Women: 63.0%  Home  Multiple exercises 2-5 times a day; 16 wks  “[P]rotocol included 11–12 exercises…The control group underwent a standard postoperative home exercise program…The research physiotherapist instructed the control group participants to follow this standard program several times a day for 16 weeks starting after discharge from the hospital. Similar to the exergame group, guidance was provided at the exercise laboratory baseline visit immediately after randomization. Face-to-face individual guidance was provided for 5 to 10 minutes by the same research physiotherapist who had conducted the baseline assessment.” | **Primary: Oxford knee score 12-item questionnaire, TUG**  **Pain intensity (2, 4 mo)**   - VAS   **Physical performance (4 mo)**   - SPPB - Active and passive knee flexion and extension, using universal goniometer - Muscle force flexion and extension, assessed with Metitur Good Strength dynamometer in Jyväskylä (Newton [N]) and a Con-Trex Multijoint dynamometer (Newton-meter [Nm]) in Turku from the operated |
| Piqueras et al, 2013[46]  High  3 months  An acute-care university general hospital, Barcelona  Partially financed by Telefónica Research and Development. | **Inclusion**: "[S]uccessful primary TKA surgery; post-TKA active range of motion: flexion 80º and extension –10º, without signs of stiffness; ability to walk with the use of a walking aid; ability to read and understand Spanish; ability to understand and accept the trial procedures and to sign an informed consent form in accordance with national legislation."  **Exclusion**: "…[S]ensory, cognitive and/or praxic impairment; concomitant medical conditions that may influence the rehabilitation process; discharge to destination other than home; patients with any local or systemic complication (e.g. surgical wound infection, suspicion of deep vein thrombosis) in the 3-month follow-up period were also excluded." | N=90  Age, mean (SD):  NR  Women: 83%  Home/Clinic or health care facility  1 hour sessions; 10 days  "The IVT is an interactive virtual software-hardware platform that facilitates the development of remote rehabilitation therapy for multiple diseases...[The] patient receives the information needed to perform the exercises and the therapist can remotely monitor the patient’s performance. For the purpose of this trial, the IVT system was designed for lower limb motor recovery in patients undergoing TKA[...]Wireless sensors (WAGYRO) including a 3-axis accelerometer and two self-powered gyroscopes [...] connected to the patient and allow calculation of their movement trajectories. [I]nteractive software with a 3D avatar that demonstrates the exercises to be undertaken, while patients reproduce the movements[...]Web portal for the therapist: receives data and records them for evaluation, with the option to modify the therapy as the rehabilitation evolves[...]The therapist supervised patients remotely on a daily basis from the hospital to check that they were performing the therapy, adjusted the prescribed therapy as appropriate, and contacted the patient via telephone as necessary." | N= N=91  Age, mean (SD):  NR  Women: 62.5%  Home/Clinic or health care facility  1 hour sessions; 10 days  "In all cases, functional rehabilitation started the day after TKA. All participants were instructed by a physical therapist in weight bearing to tolerance with an assistive device and underwent inpatient care. (mean length of stay 6.2 days) and outpatient intervention (outpatient physical therapy or IVT) for the first 3 weeks after surgery. Inpatient care consisted of assisted walking within 24 h, knee range of motion exercises and preparing for the return home. After hospital discharge, conventional physical therapy consisted of a 2-week face-to-face rehabilitation programme (progressive exercise and instruction including knee range of motion, gait training, and instructions in negotiating stairs and community-related obstacles). Any signs of adverse knee joint responses (*eg*, increased swelling or pain) resulted in a lowering of the intensity, frequency and duration of the exercises or elimination of a rehabilitation component." | **Primary outcome NR**  **Pain intensity (2 wk, 3 mo)**   - VAS   **Physical performance (2 wk, 3 mo)**   - TUG - Quadriceps muscle strength, measured in kg (NMMT dynamometer) - Hamstring muscle strength, measured in kilograms (NMMT dynamometer) - Active Knee extension, measured in degrees with a goniometer - Active Knee flexion, measured in degrees with a goniometer |
| Prvu Bettger et al, 2020[47]  Some concerns  12 weeks  Two academic medical centers and 2 independent private practices  Reflexion Health, “the manufacturer of the VERA system” | **Inclusion**:  "Patients who had an in-person clinic visit at least 10 days prior to TKA. Patients who were ≥18 years of age, who were scheduled for TKA for the treatment of nontraumatic conditions, and who had a Risk Assessment and Prediction Tool (RAPT) score of ≥ 6 (indicating expected discharge to home after surgical hospitalization) were eligible."  **Exclusion**: "[P]atients who were scheduled to undergo bilateral or staged bilateral TKA, who were in a nursing home prior to surgery, or who were unable or unwilling to provide informed consent." | N=153  Age, mean (SD):  65.4 (7.7)  Female: 59.6%  Home/Clinic or health care facility  NR; NR  "VERA is a cloud-based virtual telehealth system that functions with use of 3-dimensional (3D) tracking technology to quantify pose and motion, an avatar (digitally simulated coach) to demonstrate and guide activity, visual and audible instructions and immediate feedback on exercise quality, and a virtual video connection for synchronous telehealth visits with an assigned intervention telehealth physical therapist. Individualized prescribed therapy regimens were electronically programmed for patients through the clinician interface prior to surgery...  The VERA system tracked activity, performance, exercise quality, and adherence; the telehealth therapist monitored the patient’s progress asynchronously. Patients had a video visit with their telehealth therapist in the week after hospital discharge and weekly thereafter to review progress and to revise the therapy regimen accordingly. The telehealth therapist provided remote clinician oversight to the patients for the duration of the intervention and communicated progress to each clinical site ahead of the patients’ 2 and 6-week postoperative visits. Patients and the telehealth therapist mutually agreed when therapy goals were met for discharge from virtual PT." | N=153  Age, mean (SD):  65.1 (9.2)  Female: 65.4%  Home/Clinic or health care facility  NR; NR  "Patients in the usual care group followed their care team’s recommendations for all preoperative and postoperative medical and rehabilitative care." | **Primary outcome: Total healthcare costs 12 weeks after discharge**  **Pain related functioning or interference (6, 12 wk)**   - KOOS   **Pain intensity (12 wk)**   - NRS   **Adverse events (12 wk)**   - Number of falls, patient-reported   **Physical performance (6 wk)**   - Knee Extension (degrees) - Knee Flexion (degrees) - 10-meter gait speed   **Quality of life (12 wk)**   - PROMIS physical function - PROMIS mental health   **Other non-eligible outcomes reported**   - Healthcare costs, total and by different services - Hospital readmissions, patient-reported |
| Shim et al, 2023[48]  Some concerns  24 weeks  Two academic medical centers, Seoul  Korea Health Technology R&D Project through the Korea Health Industry Development Institute; Ministry of Health Welfare, Republic of Korea (grant no.: HR19C0781). | **Inclusion**:  “Participants aged ≥ 50 years who underwent TKA and were discharged home.”  **Exclusion**: "[P]articipants with a history of osteotomy, severe neurological deficits, infection in the  affected knee, inability to perform exercises due to severe  comorbidities, or inability to participate in the rehabilitation  program for other reasons.” | N=28  Age, mean (SD):  68.6 (5.8)  Women: 82.1%  Home  30 min, daily; 12 wks  “[AR] group performed brochure-based exercises in the early phase... In the advanced phase, exercise was performed using an AR-based digital healthcare system... At levels 1–4, participants performed range of motion and isometric strengthening of lower extremities while lying down or sitting in a chair. At levels 5–9, participants performed a program designed to train stretching and isotonic strengthening of lower extremities while standing with support. At levels 10–12, participants performed a program designed to train stretching and isotonic strengthening, including lunges and squats, while standing without support… The exercises were displayed on the screen (avatar plus written summaries). Participants…were provided with real-time feedback on the screen... The exercise performance and motion accuracy were recorded online and reviewed by a physician. Participants were provided detailed feedback on their performance at the outpatient clinic visits." | N=28  Age, mean (SD):  73.0 (4.6)  Women: 75.0%  Home  3-5 sets of exercises (10 repetitions each); 12 wks  "Both groups performed the same exercises for the same duration, but the types of instructions provided at home differed...Participants in the [conventional rehab] group performed brochure-based home exercises, according to the standard rehabilitation protocol for patients after TKA... In the early phase, an exercise program consisting of ankle pumping, ROM, and isometric/isotonic exercise was performed in a sitting or lying position. In addition, gait training using a walker and stair up-and-down training were performed. In the advanced phase, participants performed cane/self-gait training and strengthening exercise, such as squats and lunges.” | **Primary: 4-m gait speed**  **Pain related functioning or interference (3, 12, 24 wk)**   - WOMAC   **Pain intensity (3, 12, 24 wk)**   - NRS   **Physical performance (3, 12, 24 wk)**   - Berg balance scale - ROM - Quadriceps strength - Hamstring strength   **Quality of life (3, 12, 24 wk)**   - EuroQoL-5D-5L |

3D=three-dimensional; AR=augmented reality; BMI=body mass index; CPM=continuous passive motion; EuroQoL-5D=EuroQoL 5-dimension health survey; EuroQoL-5D-5L=EuroQoL 5-dimension 5-level health survey; HSS=Hospital for Special Surgery score; IDR=interactive digital rehabilitation; KOOS=Knee Injury and Osteoarthritis Outcome Score; min=minutes; N=number of participants; NR=not reported; NRS=numeric rating scale; OA=osteoarthritis; PROMIS=Patient-Reported Outcomes Measurement Information System; PT=physical therapy; RAPT=Risk Assessment and Prediction Tool; ROM=range of motion; SD=standard deviation; SF-36=36-Item Short Form Health Survey; SPPB=Short Physical Performance Battery; TKA=total knee arthroplasty; TUG=Timed Up and Go test; VAS=Visual Analog Scale; VERA=Virtual Exercise Rehabilitation Assistant; VR=virtual reality; WOMAC=Western Ontario and McMaster Universities Osteoarthritis Index

References

31. Eichler S, Salzwedel A, Rabe S, et al. The effectiveness of telerehabilitation as a supplement to rehabilitation in patients after total knee or hip replacement: randomized controlled trial. JMIR Rehabil Assist Technol. Nov 7, 2019;6(2):e14236. [doi: 10.2196/14236] [Medline: 31697239]

33. Fuchs L, Kluska A, Novak D, Kosashvili Y. The influence of early virtual reality intervention on pain, anxiety, and function following primary total knee arthroplasty. Complement Ther Clin Pract. Nov 2022;49:101687. [doi: 10.1016/j.ctcp.2022.101687] [Medline: 36347152]

35. Janhunen M, Katajapuu N, Paloneva J, et al. Effects of a home-based, exergaming intervention on physical function and pain after total knee replacement in older adults: a randomised controlled trial. BMJ Open Sport Exerc Med. 2023;9(1):e001416. [doi: 10.1136/bmjsem-2022-001416] [Medline: 36896366]

[37. Jin C, Feng Y, Ni Y, Shan Z. Virtual reality intervention in postoperative rehabilitation after total knee arthroplasty: A prospective and randomized controlled clinical trial. Int J Clin Exp Med. 2018;11(6):6119-6124. URL: www.ijcem.com](www.ijcem.com)

40. Mazurek J, Cieślik B, Wrzeciono A, Gajda R, Szczepańska-Gieracha J. Immersive virtual reality therapy is supportive for orthopedic rehabilitation among the elderly: a randomized controlled trial. J Clin Med. Dec 14, 2023;12(24):7681. [doi: 10.3390/jcm12247681] [Medline: 38137750]

43. Nishitha K, Anitha A, Thaheera D. Effectiveness of virtual reality-based rehabilitation and high-intensity exercise program for total knee arthroplasty patients: a randomised controlled trial. J Clin Diagn Res. 2024;18(11). [doi: 10.7860/JCDR/2024/71122.20263]

46. Piqueras M, Marco E, Coll M, et al. Effectiveness of an interactive virtual telerehabilitation system in patients after total knee arthoplasty: a randomized controlled trial. J Rehabil Med. Apr 2013;45(4):392-396. [doi: 10.2340/16501977-1119] [Medline: 23474735]

47. Prvu Bettger J, Green CL, Holmes DN, et al. Effects of virtual exercise rehabilitation in-home therapy compared with traditional care after total knee arthroplasty: VERITAS, a randomized controlled trial. J Bone Joint Surg Am. Jan 15, 2020;102(2):101-109. [doi: 10.2106/JBJS.19.00695] [Medline: 31743238]

48. Shim GY, Kim EH, Lee SJ, et al. Postoperative rehabilitation using a digital healthcare system in patients with total knee arthroplasty: a randomized controlled trial. Arch Orthop Trauma Surg. Oct 2023;143(10):6361-6370. [doi: 10.1007/s00402-023-04894-y] [Medline: 37129691]
