# Supplementary material for: Extended Reality Interventions for Osteoarthritis of the Knee and Recovery After Total Knee Arthroplasty: Systematic Review and Meta-Analyses
Source: J Med Internet Res. 2026 Sep 8;28:e84753. doi: 10.2196/84753 (PMC13555783; doi:10.2196/84753)
Supplement: Supplementary file 8 [file jmir-v28-e84753-s010.docx]

| **Author, Year**  **VR or IDR**  **Risk of Bias** | **Effect Measure** | **Intervention**  **Baseline mean (SD)**  **Follow-up mean (SD), mean change** | **Comparator**  **Baseline mean (SD)**  **Follow-up mean (SD), mean change** | **Comparison** |
| --- | --- | --- | --- | --- |
| **Pain-related functioning or interference** | | | | |
| Buyukyilmaz et al, 2024[28]  IDR  High | WOMAC-total | 47.1 (12.1)  3 wk: 41.5 (10.6), -5.6*  8 wk: 39.6 (10.3), -7.5* | 53.0 (11.2)  3 wk: 48.5 (9.2), -4.5*  8 wk: 49.9 (10.2), -3.1* | Diff ∆*:  3 wk: -1.1  8 wk: -4.4 |
| Dal Erdogan et al, 2025[30]  IDR  High | WOMAC-total | IDR+Conventional Rehabilitation  61.9 (7.9)  3 wk: 29.7 (15.4), -32.2* | Conventional Rehabilitation  65.0 (6.9)  3 wk: 35.6 (7.8), -29.4* | Diff ∆*:  3 wk: -2.8 |
|  |  |  | VRT session without rehabilitation  65.2 (11.0)  3 wk: 51.8 (11.0), -13.4* | Diff ∆*:  3 wk: -18.8 |
|  | WOMAC-function | IDR+Conventional Rehabilitation  47.3 (7.3)  3 wk: 23.4 (13.4), -23.9* | Conventional Rehabilitation  49.1 (6.4)  3 wk: 28.3 (7.8), -20.8* | Diff ∆*:  3 wk: -3.1 |
|  |  |  | VRT session without rehabilitation  50.1 (9.4)  3 wk: 41.7 (9.4), -8.4* | Diff ∆*:  3 wk: -15.5 |
|  | WOMAC-pain | IDR+Conventional Rehabilitation  10.3 (1.8)  3 wk: 3.7 (2.1), -6.6* | Conventional Rehabilitation  10.3 (1.8)  3 wk: 4.6 (2.4), -5.7* | Diff ∆*:  3 wk: -0.9 |
|  |  |  | VRT session without rehabilitation  10.7 (1.8)  3 wk: 6.8 (1.8), -3.9* | Diff ∆*:  3 wk: -2.7 |
|  | WOMAC-stiffness | IDR+Conventional Rehabilitation  3.9 (1.1)  3 wk: 2.3 (1.2), -1.6* | Conventional Rehabilitation  4.7 (1.1)  3 wk: 3.4 (1.1), -1.3* | Diff ∆*:  3 wk: -0.3 |
|  |  |  | VRT session without rehabilitation  3.8 (1.2)  3 wk: 2.8 (1.2), -1.0* | Diff ∆*:  3 wk: -0.6 |
| Elshazly et al, 2016[32]  AR  Some concerns | WOMAC-total | 71.6 (3.4)  4 wk: 39.8 (7.8), -31.9*  8 wk: 14.6 (3.1), -57.0* | Sensorimotor training (SMT)  71.7 (2.8)  4 wk: 53.3 (3.8), -18.4*  8 wk: 34.1 (3.9), -37.6* | Diff Δ*:  4 wk: -13.5  8 wk: -19.5 |
|  |  |  | Conventional exercise training (CET)  71.9 (3.05)  4 wk: 54.1 (6.1), -17.8*  8 wk: 44.8 (4.3), -27.2* | Diff Δ*:  4 wk: -14.05  8 wk: -29.9 |
| Jarungvittayakon et al, 2025[36]  IDR  Some concerns | KOOS | 63.0 (16.9)  6 wk: 75.0 (16.5), 12.0* | 65.4 (17.2)  6 wk: 69.9 (14.4), 4.5* | Diff ∆*:  6 wk: 7.5 |
| Lin et al, 2020[38]  AR  Some concerns | WOMAC-function | 505.1 (328.4)  2 wk: 520.3 (296.7),15.2*  4 wk: 510.6 (303.5), 5.5*  8 wk: 472.5 (300.1), -32.6*  16 wk: 456.4 (298.2), -48.7* | 581.0 (383.8)  2 wk: 557.1 (218.5), -23.9*  4 wk: 526.2 (314.6), -54.8*  8 wk: 458.4 (331.1), -122.6*  16 wk: 449.5 (220.2), -131.5* | Diff Δ*:  2 wk: 39.1  4 wk: 60.3  8 wk: 90.0  16 wk: 82.8 |
|  | WOMAC-pain | 161.2 (114.7)  2 wk: 160.9(109.4), -0.3*  4 wk: 155.6 (103.1), -5.6*  8 wk: 119.8 (89.5), -41.4*  16 wk: 102.3 (88.9), -58.9* | 170.2 (121.3)  2 wk: 169.2 (120.1), -1.0*  4 wk: 160.7 (118.2), -9.5*  8 wk: 148.7 (106.7), -21.5*  16 wk: 106.4 (97.3), -63.8* | Diff Δ*:  2 wk: 0.7  4 wk: 3.9  8 wk: -19.9  16 wk: 4.9 |
|  | WOMAC-stiffness | 76.9 (60.4)  2 wk: 74.3 (59.1), -2.6*  4 wk: 72.8 (56.7), -4.1*  8 wk: 69.9 (52.2), -7.0*  16 wk: 64.4 (52.1), -12.5* | 65.2 (53.7)  2 wk: 66.1 (52.4), 0.9*  4 wk: 67.2 (53.2), 2.0*  8 wk: 61.6 (51.6), -3.6*  16 wk: 63.4 (55.7), -1.8* | Diff Δ*:  2 wk: -3.5  4 wk: -6.1  8 wk: -3.4  16 wk: -10.7 |
| Lo et al, 2024  VR[39]  Some concerns | WOMAC-total | 902.9 (454.8)  6 wk: 926.7 (544.5), 23.8*  12 wk: 791.3 (425.1), -111.6* | 752.0 (549.3)  6 wk: 782 (444.7), 30.0*  12 wk: 742.4 (510.5), -9.6* | Diff ∆*:  6 wk: -6.2  12 wk: -102.0 |
|  | WOMAC-function | 622.1 (339.4)  6 wk: 653.8 (384.5), 31.7*  12 wk: 560.6 (312.7), -61.5* | 522.5 (394.0)  6 wk: 530.5 (326.0), 8.0*  12 wk: 513.7 (364.7), -8.8* | Diff ∆*:  6 wk: 23.7  12 wk: -52.7 |
|  | WOMAC-pain | 189.5 (96.5)  6 wk: 184.6 (117.5), -4.9*  12 wk: 160.2 (82.4), -29.3* | 163.9 (115.3)  6 wk: 187.5 (102.7), 23.6*  12 wk: 160.9 (112.3), -3.0* | Diff ∆*:  6 wk: -28.5  12 wk: -26.3 |
|  | WOMAC-stiffness | 91.3 (46.4)  6 wk: 87.8 (51.2), -3.5*  12 wk: 68.8 (42.3), -22.5* | 65.6 (54.5)  6 wk: 64.1 (40.3), -1.5*  12 wk: 69.0 (47.4), 3.4* | Diff ∆*:  6 wk: -2.0  12 wk: -25.9 |
| Mete et al, 2022[41]  IDR  High | WOMAC-total | Means NR  Medians (IQR):  19.7 (18.2, 21)  6 wk: 7.59 (4.95, 9.4) | Means NR  Medians (IQR):  15.1 (9.3, 18)  6 wk: 9.9 (6.4, 11.5) | Diff Δ: NC |
|  | WOMAC-function | Means NR  Medians (IQR):  6.74 (6.4, 7.2)  6 wk: 2.35 (2, 3.38) | Means NR  Medians (IQR):  5.2 (5.7, 6.4)  6 wk: 3.23 (2.45, 3.18) | Diff Δ: NC |
| Nambi et al, 2020[42]  IDR  Some concerns | WOMAC-total | 72.33 (4.2)  4 wk: 34.1 (3.4), -38.2*  8 wk: 22.4 (2.1), -49.9*  3 mo: 11.2 (2.1), -61.1* | SMT:  72.47 (4.5)  4 wk: 56.3 (3.8), -16.2*  8 wk: 32.4 (2.8), -40.1*  3 mo: 25.3 (2.1), -47.2* | Diff Δ*:  4 wk: -22.1  8 wk: -9.8  3 mo: -13.9 |
|  |  |  | CET:  71.2 (3.8)  4 wk: 62.3 (3.2),  8 wk: 52.3 (3.2), -18.9*  3 mo: 35.2 (2.8), -36.0* | Diff Δ*:  4 wk: -29.3  8 wk: -30.9  3 mo: -25.1 |
| Oliveira et al, 2024[44]  IDR  High | WOMAC-total | 36.2 (13.4)  8 wk: 21.7 (11.4), -14.5* | 38.4 (14.4)  8 wk: 24.5 (16), -13.9* | Diff ∆*:  8 wk: -0.6 |
| Ozlu et al, 2023[45]  VR  High | WOMAC-total | 31.66 (6.75)  3 wk: 23.1 (8.7), -8.6*  7 wk: 26.4 (9.4), -5.2* | 33.0 (7.86)  3 wk: 33.1 (7.9), 0.1*  7 wk: 32.9 (7.9), -0.2* | Diff Δ*:  3 wk: -8.7  7 wk: -5.0 |
| Sonmez et al, 2025[49]  IDR  Some concerns | WOMAC-function | 19.5 (5.9)  3 wk: 9.0 (5.6), -10.55* | 20.4 (4.2)  3 wk: 14.6 (4.5), -5.74* | Diff ∆*:  3 wk: -4.7 |
|  | WOMAC-pain | 6.3 (3.5)  3 wk: 3.2 (1.3), -3.05 | 7.5 (4.4)  3 wk: 5.2 (2.6), -2.3* | Diff ∆*:  3 wk: -0.8 |
| **Pain intensity or severity** | | | | |
| Carvajal-Parodi et al, 2025[29, 34]  IDR  Low | VAS | 46.7 (19.7)  3 wk: 28.7 (16.4), -18*  6 wk: 30.9 (20.8), -15.8*  10 wk: 14.0 (8.2), -32.7*  14 wk: 32.0 (23.6), -14.7* | 46.3 (24.1)  3 wk: 33.7 (25.9), -12.6*  6 wk: 40.4 (27.4), -5.9*  10 wk: 39.5 (18.3), -6.8*  14 wk: 38.5 (26.6), -7.8* | Diff ∆*:  3 wk: -5.4  6 wk: -9.9  10 wk: -25.9  14 wk: -6.9 |
| Dal Erdogan et al, 2025[30]  IDR  High | VAS-movement | IDR+Conventional Rehabilitation  6.4 (1.2)  3 wk: 2.6 (1.6), -3.8* | Conventional Rehabilitation  6.1 (1.0)  3 wk: 2.4 (1.6), -3.7* | Diff ∆*:  3 wk: -0.1 |
|  |  |  | VRT session without rehabilitation  6.3 (1.3)  3 wk: 4.4 (1.3), -1.9* | Diff ∆*:  3 wk: -1.9 |
|  | VAS-rest | IDR+Conventional Rehabilitation  6.1 (1.2)  3 wk: 1.5 (1.5), -4.6* | Conventional Rehabilitation  6.1 (1.2)  3 wk: 2.1 (1.8), -4.0* | Diff ∆*:  3 wk: -0.6 |
|  |  |  | VRT session without rehabilitation  5.9 (1.2)  3 wk: 3.7 (1.2), -2.2* | Diff ∆*:  3 wk: -2.4 |
| Elshazly et al, 2016[32]  VR  Some concerns | VAS | 6.81 (0.87)  4 wk: 3.7 (1.1), -3.1*  8 wk: 2.9 (0.97), -3.9* | SMT:  6.62 (1.17)  4 wk: 5.4 (0.96), -1.2*  8 wk: 4.5 (0.8), -2.1* | Diff Δ*:  4 wk: -1.9  8 wk: -1.8 |
|  |  |  | CET:  6.68 (0.84)  4 wk: 5.37 (0.9), -1.3*  8 wk: 4.74 (0.7), -1.9* | Diff Δ*:  4 wk: -1.8  8 wk: -1.97 |
| Jarungvittayakon et al, 2025[36]  IDR  Some concerns | VAS at rest | Means NR  Medians (IQR):  2 (0-6)  6 wk: 1 (0-5) | Means NR  Medians (IQR):  2 (0-5)  6 wk: 2 (0-5) | Diff Δ: NC |
|  | VAS in motion | 5.5 (2.0)  6 wk: 3.6 (2.1), -1.9* | 6.5 (2.4)  6 wk: 5.9 (2.2), -0.6* | Diff ∆*:  6 wk: -1.3 |
| Lin et al, 2020[38]  IDR  Some concerns | Graded Chronic Pain Scale, Overall Pain Grade | 1.5 (0.4)  2 wk: 1.3 (0.3), -0.2*  4 wk:1.2 (0.3), -0.3*  8 wk:1.2 (0.2), -0.3*  16 wk: 1.0 (0.2), -0.5* | 1.4 (0.5)  2 wk: 1.4 (0.4), 0.0*  4 wk: 1.2 (0.1), -0.2*  8 wk: 1.3 (0.3), -0.1*  16 wk: 1.1 (0.2), -0.3* | Diff Δ*:  2 wk: -0.2  4 wk: -0.1  8 wk: -0.2  16 wk: -0.2 |
|  | Graded Chronic Pain Scale, disability score | 38.6 (20.8)  2 wk: 35.7 (20.3), -2.9*  4 wk: 34.2 (21.2), -4.4*  8 wk: 33.2 (19.3), -5.4*  16 wk: 34.4 (19.2), -4.2* | 39.3 (21.5)  2 wk: 38.3 (21.7), -1.0*  4 wk: 37.6 (20.4), -1.7*  8 wk: 36.7 (19.6), -2.6*  16 wk: 35.6 (20.3), -3.7* | Diff Δ*:  2 wk: -1.9  4 wk: -2.7  8 wk: -2.8  16 wk: -0.5 |
|  | Graded Chronic Pain Scale, pain intensity | 47.8 (20.3)  2 wk: 42.5(19.5), -5.3*  4 wk: 46.2 (19.3), -1.6*  8 wk: 44.6 (20.1), -3.2*  16 wk: 43.3 (19.4), -4.5* | 48.4 (20.7)  2 wk: 49.7 (19.5), 1.3*  4 wk: 48.2 (21.2), -0.2*  8 wk: 47.3 (19.1), -1.1*  16 wk: 46.5 (18.2), -1.9* | Diff Δ*:  2 wk: -6.6  4 wk: -1.4  8 wk: -2.1  16 wk: -2.6 |
| Lo et al, 2024  VR[39]  Some concerns | NRS | 5.9 (1.9)  6 wk: 5.5 (1.7), -0.4*  12 wk: 4.8 (1.8), -1.1* | 4.8 (1.5)  6 wk: 5.1 (1.2), 0.3*  12 wk: 4.6 (2.4), -0.2* | Diff ∆*:  6 wk: -0.7  12 wk: -0.9 |
| Mete et al, 2022[41]  IDR  High | VAS at rest | Means NR  Medians (IQR):  32.2 (20.75, 40  6 wk: 10 (0, 12.5) | Means NR  Medians (IQR):  36.6 (30, 40)  6 wk: 20 (10, 20) | Diff Δ: NC |
|  | VAS with activity | Means NR  Medians (IQR):  74.2 (60, 80)  6 wk: 30 (30, 40) | Means NR  Medians (IQR):  65.2 (50, 70)  6 wk: 40 ( 30, 40) | Diff Δ: NC |
|  | VAS at night | Means NR  Medians (IQR):  43 (27.5, 40)  6 wk: 10 (0, 20) | Means NR  Medians (IQR):  44 (20, 50)  6 wk: 20 (10, 30) | Diff Δ: NC |
|  | WOMAC-pain | Means NR  Medians (IQR):  6 (5.37, 7.12)  6 wk: 2.25 (1.5, 3) | Means NR  Medians (IQR):  4.5 (4.3, 6)  6 wk: 3 (2.5, 4) | Diff Δ: NC |
| Nambi et al, 2020[42]  IDR  Some concerns | VAS | 7.2 (0.5)  4 wk: 3.3 (0.4), -3.9*  8 wk: 2.5 (0.4), -4.7*  3 mo: 0.5 (0.3), -6.7* | SMT: 7.4 (0.4)  4 wk: 5.8 (0.5), -1.6*  8 wk: 3.5 (0.5), -3.9*  3 mo: 1.5 (0.4), -5.9* | Diff Δ*:  4 wk: -2.3  8 wk: -0.8  3 mo: -0.8 |
|  |  |  | Control: 7.3 (0.4)  4 wk: 6.5 (0.5), -0.8*  8 wk: 4.2 (0.5), -3.1*  3 mo: 3.8 (0.4), -3.5* | Diff Δ*:  4 wk: -3.1  8 wk: -1.6  3 mo: -3.2 |
| Oliveira et al, 2024[44]  IDR  High | VAS | 7.5 (1.2)  8 wk: 2.7 (2.0), -4.8* | 8.0 (1.5)  8 wk: 3.0 (2.4), -5.0* | Diff ∆*:  8 wk: 0.2 |
| Ozlu et al, 2023[45]  VR  High | Visual analog scale (VAS) | 5.57 (0.88)  3 wk: 4.11 (1.34), -1.46*  7 wk: 4.05 (0.72), -1.52* | 5.78 (0.74)  3 wk: 5.05 (1.43), -0.73*  7 wk: 5.36 (0.99), -0.42* | Diff Δ*:  3 wk: -0.73  7 wk: -1.1 |
| **Adverse events** | | | | |
| Carvajal-Parodi et al, 2025[29]  IDR  Low | Adverse events (during 14 wk study period) | “No adverse events, such as falls, fainting, nausea, or severe pain, were reported.” | | NR |
| Lin et al, 2020[38]  IDR  Some concerns | Adverse effects (during 4 wks of intervention) | "No adverse effects were reported during or after treatment in either group." | | NR |
| Lo et al, 2024  VR[39]  Some concerns | Cybersickness | “Adverse events included 5 (33.3%) participants reporting  cybersickness in the VR group.” | | NR |
| Sonmez et al, 2025[49]  IDR  Some concerns | Adverse effects during 3 wk study period | "No adverse events were observed in either group during the study period, and participants were not notified of any adverse events related to the intervention." | | NR |
| **Pain Catastrophizing & Kinesiophobia** | | | | |
| Mete et al, 2022[41]  IDR  High | TSK | Means NR  Medians (IQR):  46.5 (42.7, 50)  6 wk: 39 (36.7, 40) | Means NR  Medians (IQR):  48 (45, 49.25)  6 wk: 45.5 (41, 48) | Diff Δ: NC |
| **Quality of life** | | | | |
| Elshazly et al, 2016[32]  IDR  Some concerns | CDC Health Related Quality of Life | 1.35 (0.48)  4 wk: 3.1 (0.5), 1.8*  8 wk: 4.5 (0.6), 3.2* | SMT:  1.60 (0.50)  4 wk: 2.5 (0.5), 0.9*  8 wk: 3.3 (0.5), 1.7* | Diff Δ*:  4 wk: 0.9  8 wk: 1.4 |
|  |  |  | CET:  1.6 (0.51)  4 wk: 2.2 (0.4), 0.7*  8 wk: 2.5 (0.5), 0.95* | Diff Δ*:  4 wk: 1.1  8 wk: 2.2 |
| Lin et al, 2020[38]  IDR  Some concerns | WHOQOL-BREF Physical | 58.1 (10.2)  2 wk: 58.4 (9.1), 0.3*  4 wk: 59.2 (7.4), 1.1*  8 wk: 59.3 (7.5), 1.2*  16 wk: 61.1 (6.6), 3.0* | 53.4 (12.1)  2 wk: 53.1 (11.5), -0.3*  4 wk: 53.2 (11.2), -0.2*  8 wk: 54.1 (10.3), 0.7*  16 wk: 54.5 (10.9), 1.1* | Diff Δ*:  2 wk: 0.6  4 wk: 1.3  8 wk: 0.5  16 wk: 1.9 |
|  | WHOQOL-BREF Psychological | 57.2 (10.4)  2 wk: 57.5 (10.1), 0.3*  4 wk: 58.1 (9.6), 0.9*  8 wk: 58.4 (9.7), 1.2*  16 wk: 60.0 (9.4), 2.8 | 58.7 (9.8)  2 wk: 58.9 (9.8), 0.2*  4 wk: 59.0 (9.4), 0.3*  8 wk: 59.4 (8.9), 0.7*  16 wk: 60.2 (8.8), 1.5 | Diff Δ*:  2 wk: 0.1  4 wk: 0.6  8 wk: 0.5  16 wk: 1.3 |
|  | WHOQOL-BREF, social | 57.3 (12.60)  2 wk: 57.4 (11.2), 0.1*  4 wk: 58.2 (10.8), 0.9*  8 wk: 58.4 (10.1), 1.1*  16 wk: 60.3 (9.9), 3.0* | 54.4 (12.60)  2 wk: 55.9 (13.9), 1.5*  4 wk: 55.9 (14.4), 1.5*  8 wk: 57.3 (13.7), 2.9*  16 wk: 57.4 (13.2), 3.0* | Diff Δ*:  2 wk: -1.4  4 wk: -0.6  8 wk: -1.8  16 wk: 0.0 |
|  | WHOQOL-BREF, environmental | 60.4 (9.8)  2 wk: 60.8 (9.9), 0.4*  4 wk: 61.1 (10.1), 0.7*  8 wk: 61.3 (9.2), 0.9*  16 wk: 61.9 (9.3), 1.5* | 58.9 (10.6)  2 wk: 59.2 (10.2), 0.3*  4 wk: 59.3 (9.8), 0.4*  8 wk: 59.7 (10.1), 0.8*  16 wk: 60.2 (9.6), 1.3* | Diff Δ*:  2 wk: 0.1  4 wk: 0.3  8 wk: 0.1  16 wk: 0.2 |
| Lo et al, 2024  VR[39]  Some concerns | Euro-Qol-VAS | 62.0 (19.6)  6 wk: 67.6 (18.6), 5.6*  12 wk: 71.6 (12.8), 9.6* | 67.3 (17.2)  6 wk: 70.8 (12.0), 3.5*  12 wk: 67.7 (17.4), 0.4* | Diff ∆*:  6 wk: 2.1  12 wk: 9.2 |
| **Physical performance** | | | | |
| Carvajal-Parodi et al, 2025[29]  IDR  Low | 2-min step  test (rep) | 54.8 (17.8)  3 wk: 69.8 (17.4), 15.0*  6 wk: 71.6 (16.4), 16.8*  10 wk: 77.5 (14.3), 22.7*  14 wk: 73.1 (20.4), 18.3* | 55.4 (11.6)  3 wk: 55.3 (12.6), -0.1*  6 wk: 57 (12.7), 1.6*  10 wk: 58.8 (13.4), 3.4*  14 wk: 58.6 (14.8), 3.2* | Diff ∆*:  3 wk: 15.1  6 wk: 15.2  10 wk: 19.3  14 wk: 15.1 |
|  | 30-s chair  stand (rep) | 10.2 (3.3)  3 wk: 12.4 (2.5), 2.2*  6 wk: 12.8 (2.1), 2.6*  10 wk: 13.9 (2.4), 3.7*  14 wk: 13.0 (2.8), 2.8* | 9.0 (2.3)  3 wk: 10.4 (2.5),1.4*  6 wk: 10.4 (2.3), 1.4*  10 wk: 11.1 (1.9), 2.1*  14 wk: 10.8 (1.9), 1.8* | Diff ∆*:  3 wk: 0.8  6 wk: 1.2  10 wk: 1.6  14 wk: 1.0 |
|  | TUG | 10.0 (2.3)  3 wk: 8.2 (1.6), -1.4*  6 wk: 7.5 (1.2), -2.1*  10 wk: 7.0 (0.9), -2.6*  14 wk: 7.3 (1.1), -2.3* | 10.0 (2.4)  3 wk: 9.0 (1.7), -1*  6 wk: 8.9 (1.4), -1.1*  10 wk: 9.0 (1.5), -1*  14 wk: 9.2 (2.0), -0.8* | Diff ∆*:  3 wk: -0.4  6 wk: -1  10 wk: -1.6  14 wk: -1.5 |
| Buyukyilmaz et al, 2024[28]  IDR  High | ROM-Knee flexion | 115.4 (13.1)  3 wk: 117.0 (13.1), 1.6*  8 wk: 116.0 (12.4), 0.6* | 113.5 (8.8)  3 wk: 111.3 (9.7), -2.2*  8 wk: 111.1 (13.6), -2.4* | Diff ∆*:  3 wk: 3.8  8 wk: 3.0 |
|  | Knee flexion (kg-force) | 17.4 (6.0)  3 wk: 15.2 (2.1), -2.2*  8 wk: 15.8 (2.3), -1.6* | 13.8 (3.9)  3 wk: 18.2 (6.9), 4.4*  8 wk: 15.0 (1.9), 1.2* | Diff ∆*:  3 wk: -6.6  8 wk: -2.8 |
|  | Knee Extension | 14.4 (2.9)  3 wk: 14.9 (3.5), 0.5*  8 wk: 18.4 (2.3), 4.0* | 17.0 (2.4)  3 wk: 18.3 (3.4), 1.3*  8 wk: 16.4 (2.8), -0.6* | Diff ∆*:  3 wk: -0.8  8 wk: 4.6 |
|  | 15 degree joint position sense | 4.6 (3.1)  3 wk: 3.6 (2.1), -1.0*  8 wk: 3.8 (2.7), -0.8* | 4.6 (2.8)  3 wk: 3.6 (1.6), -1.0*  8 wk: 2.8 (1.1), -1.8* | Diff ∆*:  3 wk: 0.0  8 wk: 1.0 |
|  | 45 degree joint position sense | 3.6 (2.7)  3 wk: 3.7 (2.1), 0.1*  8 wk: 2.6 (1.8), -1.0* | 4.3 (2.1)  3 wk: 3.6 (1.7), -0.7*  8 wk: 3.1 (1.4), -1.2* | Diff ∆*:  3 wk: 0.8  8 wk: 0.2 |
|  | 75 degree joint position sense | 3.5 (2.4)  3 wk: 2.6 (1.8), -0.9*  8 wk: 2.0 (1.5), -1.5* | 3.1 (2.4)  3 wk: 2.8 (2.6), -0.3*  8 wk: 1.7 (2.2), -1.4* | Diff ∆*:  3 wk: -0.6  8 wk: -0.1 |
|  | TUG | 11.9 (4.9)  3 wk: 10.9 (3.2), -1.0*  8 wk: 10.9 (3.2), -1.0* | 9.7 (2.0)  3 wk: 9.4 (2.6), -0.3*  8 wk: 8.3 (2.2), -1.4* | Diff ∆*:  3 wk: -0.7  8 wk: 0.4 |
| Elshazly et al, 2016[32]  VR  Some concerns | Position sense | 118.9 (4.60)  4 wk: 127.6 (2.06), 8.7*  8 wk: 134.0 (1.16), 15.1* | SMT:  120.5 (4.16)  4 wk: 123.5 (2.28), 3.0*  8 wk: 127.0 (1.93), 6.5* | Diff Δ*:  4 wk: 5.7  8 wk: 8.6 |
|  |  |  | CET:  120.95 (3.79)  4 wk: 123.1 (3.29), 2.15*  8 wk: 124.45 (2.96), 3.5* | Diff Δ*:  4 wk: 6.6  8 wk: 11.6 |
| Jarungvittayakon et al, 2025[36]  IDR  Some concerns | Arc of motion | 104.8 (15.6)  6 wk: 115.3 (10.6), 10.5* | 107.4 (10.2)  6 wk: 108.8 (7.9), 1.4* | Diff ∆*:  6 wk: 9.1 |
|  | ROM Extension | 10.9 (6.2)  6 wk: 7.8 (5.7), -3.1* | 9.0 (4.5)  6 wk: 5.6 (3.6), -3.4* | Diff ∆*:  6 wk: 0.3 |
|  | ROM Flexion | 115.6 (12.2)  6 wk: 123.1 (8.4), 7.5* | 116.4 (10.1)  6 wk: 114.4 (8.0), -2.0* | Diff ∆*:  6 wk: 9.5 |
|  | TUG | 8.1 (1.5)  6 wk: 7.2 (1.4), -0.9* | 8.3 (2.6)  6 wk: 8.0 (2.3), -0.3* | Diff ∆*:  6 wk: -0.6 |
| Lin et al, 2020[38]  IDR  Some concerns | Biodex Stability System, Postural Stability | 0.7 (0.5)  2 wk: 0.6 (0.4), -0.1*  4 wk: 0.6 (0.5), -0.1*  8 wk: 0.5 (0.3), -0.2*  16 wk: 0.5 (0.2), -0.2* | 0.8 (0.4)  2 wk: 0.7 (0.5), -0.1*  4 wk: 0.6 (0.4), -0.2*  8 wk: 0.5 (0.3), -0.3*  16 wk: 0.5 (0.4), -0.3* | Diff Δ*:  2 wk: 0.0  4 wk: 0.1  8 wk: 0.1  16 wk: 0.1 |
|  | Biodex Stability System, Limits of Stability | 40.2 (12.9)  2 wk: 41.0 (12.6), 0.8*  4 wk: 42.1 (12.1), 1.9*  8 wk: 43.4 (11.5), 3.2*  16 wk: 45.5 (10.7), 5.3* | 41.5 (12.7)  2 wk: 42.1 (14.8), 0.6*  4 wk: 41.9 (14.1), 0.4*  8 wk: 42.6 (13.2), 1.1*  16 wk: 44.3 (12.4), 2.8* | Diff Δ*:  2 wk: 0.2  4 wk: 1.5  8 wk: 2.1  16 wk: 2.5 |
|  | 10 meter walking time (s) | 14.1 (7.6)  2 wk: 14.7 (6.7), 0.6*  4 wk: 12.6 (5.2), -1.5*  8 wk: 12.1 (4.9), -2.0*  16 wk: 11.2 (4.1), -2.9* | 15.8 (7.3)  2 wk: 15.3 (6.4), -0.5*  4 wk:14.4 (5.9), -1.4*  8 wk: 13.9 (5.6), -1.9*  16 wk: 13.8 (4.7), -2.0* | Diff Δ*:  2 wk: 1.1  4 wk: -0.1  8 wk: -0.1  16 wk: -0.9 |
|  | Stair ascent time (s) | 18.8 (10.3)  2 wk: 17.9 (10.2), -0.9*  4 wk: 17.3 (11.8), -1.5*  8 wk: 14.9 (12.7), -3.9*  16 wk: 14.8 (11.9), -4.0* | 17.5 (12.3)  2 wk: 16.8 (12.7), -0.7*  4 wk: 15.9 (12.4), -1.6*  8 wk:15.5 (13.2), -2.0*  16 wk: 14.9 (10.8), -2.6* | Diff Δ*:  2 wk: -0.2  4 wk: 0.1  8 wk: -1.9  16 wk: -1.4 |
|  | Stair descent time (s) | 17.9 (10.8)  2 wk: 17.8 (10.7), -0.1*  4 wk: 16.3 (10.5), -1.6*  8 wk: 15.9 (12.7), -2.0*  16 wk: 14.6 (11.9), -3.3* | 15.5 (11.3)  2 wk: 15.8 (11.2), 0.3*  4 wk: 14.5 (10.4), -1.0*  8 wk: 14.3 (10.2), -1.2*  16 wk: 14.1 (9.8), -1.4* | Diff Δ*:  2 wk: -0.4  4 wk: -0.6  8 wk: -0.8  16 wk: -1.9 |
|  | Pedalo Balance Score (percentage) | Means NR  Medians (IQR):  57 (50.2, 62.2)  6 wk: 75 (65, 80) | Means NR  Medians (IQR):  70 (60, 78.5)  6 wk: 72.5 (63.7, 80) | Diff Δ: NC |
| Mete et al, 2022[41]  IDR  High | Knee Flexion ROM (°) (6 wk) | Means NR  Medians (IQR):  100 (95, 110.5)  6 wk: 115.5 (110 and 120) | Means NR  Medians (IQR):  110 (100, 120)  6 wk: 111.5 (104.7 and 120) | Diff Δ: NC |
|  | Knee proprioception at 30° (6 wk) | Means NR  Medians (IQR)  6.25 (5.75, 8)  6 wk: 3.5 (3, 4) | Means NR  Medians (IQR):  7 (6, 8)  6 wk: 6 (5, 7) | Diff Δ: NC |
|  | Knee proprioception at 60° (6 wk) | Means NR  Medians (IQR):  8 (6, 10)  6 wk: 4 (3, 5) | Means NR  Medians (IQR):  9 (7.3, 10)  6 wk: 8 (6, 9) | Diff Δ: NC |
|  | Peak Torque of knee flexion at 120° (normalized, Nm/kg) (6 wk) | Means NR  Medians (IQR):  23.5 (14, 30)  6 wk: 36.9 (26.4, 47) | Means NR  Medians (IQR):  20 (12, 26)  6 wk: 30.8 (24.3, 40.5) | Diff Δ: NC |
|  | Peak Torque of knee extension at 120° (normalized, Nm/kg) (6 wk) | Means NR  Medians (IQR):  27.9 (21.6, 39.2)  6 wk: 52.5 (43.7, 66.2) | Means NR  Medians (IQR):  25.6 (17.7, 35.8)  6 wk: 40.2 (30.6, 60) | Diff Δ: NC |
|  | Peak Torque of knee flexion at 240° (normalized, Nm/kg) (6 wk) | Means NR  Medians (IQR):  16.4 (12.4, 23.5)  6 wk: 26 (21.3, 31.4) | Means NR  Medians (IQR):  15.8 (10, 21.4)  6 wk: 22.12 (19, 28) | Diff Δ: NC |
|  | Peak Torque of knee extension at 240° (normalized, Nm/kg) (6 wk) | Means NR  Medians (IQR):  16.4 (13, 24.8)  6 wk: 36.8 (26.3, 44.2) | Means NR  Medians (IQR):  20.3 (13.4, 26.4)  6 wk:31.7 (22.5, 39) | Diff Δ: NC |
| Oliveira et al, 2024[44]  IDR  High | Anticipatory postural adjustments, latency | 0.6 (0.1)  8 wk: 0.5 (0.1), -0.1* | 0.5 (0.1)  8 wk: .05 (0.1), -0.5* | Diff ∆*:  8 wk: 0.4 |
|  | Anticipatory postural adjustments, amplitude | 0.5 (0.2)  8 wk: 0.7 (0.2), 0.2* | 0.6 (0.2)  8 wk: 0.5 (0.1), -0.1* | Diff ∆*:  8 wk: 0.3 |
|  | Anticipatory postural adjustments, time to reach maximum | 0.3 (0.0)  8 wk: 0.3 (0.1), 0.0* | 0.3 (0.0)  8 wk: 0.3 (0.0), 0.0* | Diff ∆*:  8 wk: 0.0 |
| Ozlu et al, 2023[45]  VR  High | 6-minute walk test (m) | 525.1 (70.5)  3 wk: 525.1 (70.5), 0.0*  7 wk: 526.3 (68.5), 1.1* | 530.4 (54.6)  3 wk: 531.3 (54.6), 0.9*  7 wk: 532.5 (67.3), 2.1* | Diff Δ*:  3 wk: -0.9  7 wk: -0.9 |
|  | Berg Balance Scale | 42.8 (4.8)  3 wk: 45.3 (4.2), 2.5*  7 wk: 46.0 (4.0), 3.6* | 42.9 (5.9)  3 wk: 43.5 (6.07), 0.6*  7 wk: 43.2 (6.0), 0.3* | Diff Δ*:  3 wk: 1.9  7 wk: 2.9 |
| Sonmez et al, 2025[49]  IDR  Some concerns | Proprioception (Right) | 6.3 (4.1)  3 wk: 3.0 (3.0), -3.3* | 6.8 (6.0)  3 wk: 5.4 (4.7), -1.4* | Diff ∆*:  3 wk: -1.9 |
|  | Proprioception (Left) | 6.6 (4.9)  3 wk: 2.3 (1.8), -4.3* | 6.5 (7.1)  3 wk: 6.7 (6.8), 0.2* | Diff ∆*:  3 wk: -4.5 |
|  | TUG | 12.9 (3.1)  3 wk: 8.1 (2.0), -4.8* | 14.3 (8.5)  3 wk: 12.7 (8.1), -1.6* | Diff ∆*:  3 wk: -3.2 |
| **Adherence** | | | | |
| Carvajal-Parodi et al, 2025  IDR  Low | Adherence (participants attending ≥20 sessions) | Means NR  76.6% | Means NR  73.3% | Diff Δ*: 3.3% |
| Lo et al, 2024  VR[39]  Some concerns | TLFB-E (median bout time) | Means NR  Medians (IQR):  6 wk: 710 (429.8-1275.8)  12 wk: 1390 (662-1877.8) | Means NR  Medians (IQR):  6 wk: 618.8 (347.1-1692.8)  12 wk: 1117.5 (727.8-2988) | Diff Δ: NC |

AR=augmented reality; CDC=Centers for Disease Control and Prevention; Diff Δ=difference in change scores; IDR=interactive digital rehabilitation; IQR=interquartile range; KOOS=Knee Injury and Osteoarthritis Outcome Score; min=minutes; NC=not calculable; NR=not reported; NRS=numeric rating scale; ROM=range of motion; SD=standard deviation; TSK=Tampa Scale for Kinesiophobia; TUG=Timed Up and Go test; VAS=Visual Analog Scale; VR=virtual reality; WHOQOL-BREF=World Health Organization Quality of Life Brief Version; WOMAC=Western Ontario and McMaster Universities Osteoarthritis Index

Results

28. Büyükyilmaz G, Menek B, Tarakçi D. Closed kinetic chain exercises therapy versus Wii-based Exergame therapy in the treatment of knee osteoarthritis: randomized controlled trial. Arch Health Sci Res. 2024;11(2):133-139. [doi: 10.5152/ArcHealthSciRes.2024.23139]

29. Carvajal-Parodi C, Mendoza C, Alvarez C, et al. Effectiveness of exergames on functional physical performance in older adults with knee/hip osteoarthritis: a randomized controlled trial. J Clin Med. Apr 25, 2025;14(9):2968. [doi: 10.3390/jcm14092968] [Medline: 40363999]

30. Dal Erdoğan S, Berkan F, Armağan O, Özgen M, Çiracioğlu AM, Şahi̇n Mutlu F. The effects of virtual reality-based exercise on pain and function in older adults with knee osteoarthritis: a randomized controlled study. Turk J Geriatr. 2025;28(2). [doi: 10.29400/tjgeri.2025.432]

[32. Elshazly FAA, Nambi GS, Elnegamy TE. Comparative study on virtual reality training (VRT) over sensory motor training (SMT) in unilateral chronic osteoarthritis—A randomized control trial. Int J Med Res Health Sci. 2016;5(8):7-16. URL: https://www.ijmrhs.com/](https://www.ijmrhs.com/)

34. Guede-Rojas F, Mendoza C, Rodríguez-Lagos L, et al. Effects of non-immersive virtual reality exercise on self-reported pain and mechanical hyperalgesia in older adults with knee and hip osteoarthritis: a secondary analysis of a randomized controlled trial. Medicina (B Aires). 2025;61(7):1122. [doi: 10.3390/medicina61071122]

36. Jarungvittayakon C, Sa-Ngasoongsong P, Chockchaisakul D, Bamrungchaowkasem J, Wongsak S. Effectiveness of mobile exergaming with sensor-based visual feedback as an adjunct therapy for home-based quadriceps exercise training in knee osteoarthritis: a prospective randomized controlled trial. Life (Basel). Nov 12, 2025;15(11):1738. [doi: 10.3390/life15111738] [Medline: 41302162]

38. Lin YT, Lee WC, Hsieh RL. Active video games for knee osteoarthritis improve mobility but not WOMAC score: a randomized controlled trial. Ann Phys Rehabil Med. Nov 2020;63(6):458-465. [doi: 10.1016/j.rehab.2019.11.008] [Medline: 31981832]

39. Lo HHM, Ng M, Fong PYH, et al. Examining the feasibility, acceptability, and preliminary efficacy of an immersive virtual reality-assisted lower limb strength training for knee osteoarthritis: mixed methods pilot randomized controlled trial. JMIR Serious Games. Sep 27, 2024;12:e52563. [doi: 10.2196/52563] [Medline: 39331525]

41. Mete E, Sari Z. The efficacy of exergaming in patients with knee osteoarthritis: a randomized controlled clinical trial. Physiother Res Int. Jul 2022;27(3):e1952. [doi: 10.1002/pri.1952] [Medline: 35470534]

42. Nambi G, Abdelbasset WK, Elsayed SH, Khalil MA, Alrawaili SM, Alsubaie SF. Comparative effects of virtual reality training and sensory motor training on bone morphogenic proteins and inflammatory biomarkers in post-traumatic osteoarthritis. Sci Rep. Sep 28, 2020;10(1):15864. [doi: 10.1038/s41598-020-72587-2] [Medline: 32985509]

44. Oliveira LKR, Marques AP, Andrade KFA, et al. Virtual reality in improving anticipatory postural adjustments to step initiation in individuals with knee osteoarthritis: a randomized controlled trial. Games Health J. Apr 2024;13(2):100-108. [doi: 10.1089/g4h.2023.0154] [Medline: 38436585]

45. Özlü A, Ünver G, Tuna Hİ, Menekşeoğlu AK. The effect of a virtual reality-mediated gamified rehabilitation program on pain, disability, function, and balance in knee osteoarthritis: a prospective randomized controlled study. Games Health J. Apr 2023;12(2):118-124. [doi: 10.1089/g4h.2022.0130] [Medline: 36603100]

49. Sönmez M, Avcı Ş, Şimşek F, Baygutalp F. Nonimmersive virtual reality-based exercises improve muscle excitability and balance in patients with knee osteoarthritis: a sham-controlled study. Games Health J. Dec 2025;14(6):493-503. [doi: 10.1177/2161783X251360443] [Medline: 40711924]
