# Supplementary material for: Extended Reality Interventions for Osteoarthritis of the Knee and Recovery After Total Knee Arthroplasty: Systematic Review and Meta-Analyses
Source: J Med Internet Res. 2026 Sep 8;28:e84753. doi: 10.2196/84753 (PMC13555783; doi:10.2196/84753)
Supplement: Supplementary file 9 [file jmir-v28-e84753-s011.docx]

| **Author, Year**  **VR or IDR**  **Risk of Bias** | **Effect Measure** | **Intervention**  **Baseline mean (SD)**  **Follow-up mean (SD),**  **mean change (SD)** | **Comparator**  **Baseline mean (SD)**  **Follow-up mean (SD),**  **mean change (SD)** | **Comparison** |
| --- | --- | --- | --- | --- |
| **Pain-related functioning or interference** | | | | |
| Eichler et al, 2019[31]  AR  High | WOMAC (3 mo) | 26.4 (18.5)  11.5 (12.7), -14.9 (13.6) | 24.8 (16.4)  13.9 (14.3), -10.9 (13.5) | Diff ∆*: -4.0  Stand. Diff Δ: -0.3* |
| Fuchs et al, 2022[33]  VR  High | Modified KOOS (6 mo) | 36.4 (15.1)  57.2 (27.3), 20.8* | 34.5 (17.0)  54.2 (25.4), 19.7* | Diff ∆ : 1.1*^*^* |
| Janhunen et al, 2023[35]  AR  Some concerns | OKS | 26.7 (6.7)  2 mo: 33.4 (6.3), 6.7  4 mo: 38.6 (6.1), 12.1 (7.0*) | 26.9 (6.5)  2 mo: 30.3 (5.5), 3.4  4 mo: 36.7 (6.7), 9.8 (7.0) | Diff ∆*:  2 mo: 3.3  4 mo: 2.3  Stand. Diff Δ*^*^*:  4 mo: 0.33,  p=0.27† |
| Jin et al, 2018[37]  VR  High | WOMAC | 45.03 (5.1)  1 mo: 32.0 (5.2), -13.0*  3 mo: 25.8 (4.2), -19.2*  6 mo: 21.6 (4.2), -23.5* | 44.18 (5.73)  1 mo: 35.1 (5.2), -9.1*  3 mo: 29.7 (5.6), -14.5*  6 mo: 26.3 (3.9), -17.9* | Diff ∆*:  1 mo: -3.9  3 mo: -4.7  6 mo: -5.6 |
| Mazurek et al, 2023[40]  VR  Some concerns | Barthel Index | 54.6 (16.5)  4 wk: 93.9 (6.5), 39.3* | 56.2 (17.4)  4 wk: 74.4 (16.8), 18.2* | Diff ∆*:  4 wk: 21.1 |
| Nishitha et al, 2024[43]  VR  High | WOMAC-total | 71.2 (0.8)  9 wk: 51.2 (1.3), -20.0*  12 wk: 17.2 (2.6), -54.0* | 71.0 (0.8)  9 wk: 62.1 (1.6), -8.9*  12 wk: 31.3 (1.1), -39.7* | Diff ∆*:  9 wk: -11.1  12 wk: -14.3 |
| Prvu Bettger et al, 2020[47]  AR  Some concerns | KOOS | 37.0 (12.0)  6 wk: 61.0 (11.5), 24.0  12 wk: 69.6 (12.1), 32.6 | 36.0 (13.0)  6 wk: 61.8 (13.5), 25.8  12 wk: 67.2 (14.3), 31.2 | Diff ∆*:  6 wk: -1.8  12 wk: 1.4 |
| Shim et al, 2023[48]  AR  Some concerns | WOMAC | 83.1 (13.0)  3 wk: 66.1 (12.5), -17*  12 wk: 46.5 (9.1), -36.6*  24 wk: 40.1 (7.0), -43.0* | 81.1 (14.4)  3 wk: 62.2 (15.0), -18.9*  12 wk: 45.3 (15.0), -35.8*  24 wk: 40.9 (16.0), -40.2* | Diff ∆*:  3 wk: 1.9  12 wk: -0.8  24 wk: -2.8 |
| **Pain intensity** | | | | |
| Fuchs et al, 2022[33]  VR  High | VAS (2 days) | Means NR  Median (IQR):  Before treatment: 6 (5-8), NC  After treatment: 4 (1-7), NC | Means NR  Median (IQR):  Before treatment: 6 (6-8), NC  After treatment: 5 (2-7),  NC | Diff ∆: NC |
| Janhunen et al, 2023[35]  AR  Some concerns | VAS | 57.1 (18.3)  2 mo: 30.5 (21.0), -26.6*  4 mo: 20.8 (20.3), -36.3* (24.4*) | 54.2 (21.6)  2 mo: 28.7 (20.0), -25.5*  4 mo: 27.0 (27.5), -26.7* (24.7*) | Diff ∆*:  2 mo: -1.1  4 mo: -9.1  Stand. Diff Δ*:  4 mo: -0.39,  p=0.18† |
| Jin et al, 2018[37]  VR  High | VAS | 7.4 (1.14)  3 days: 6.5 (0.9), -0.9*  5 days: 5.4 (0.8), -2.0*  7 days: 3.9 (0.6), -3.5* | 7.4 (1.3)  3 days: 6.8 (1.1), -0.6*  5 days: 6.0 (0.9), -1.5*  7 days: 4.4 (0.8), -3.0* | Diff ∆*:  3 days: -0.3  5 days: -0.5  7 days: -0.5 |
| Mazurek et al, 2023[40]  VR  Some concerns | VAS | 5.3 (2.0)  4 wk: 0.9 (1.0), -4.4* | 4.4 (2.1)  4 wk: 2.6 (1.9), -1.8* | Diff ∆*:  4 wk: -2.6 |
| Nishitha et al, 2024[43]  VR  High | NRS | 8.56 (0.5)  4 wk: 3.4 (0.5), -5.2*  12 wk: 2.5 (0.5), -6.1* | 8.44 (0.5)  4 wk: 3.8 (0.4), -4.6*  12 wk: 3.3 (0.5), -5.1* | Diff ∆*:  4 wk: -0.5  12 wk: -0.9 |
| Piqueras et al, 2013[46]  AR  High | VAS | 3.8 (2.0)  2 wk: 3.1*, -0.7 (1.4)  3 mo: 2.0*, -1.8 (2.5) | 4.3 (1.9)  2 wk: 3.7*, -0.6 (1.9)  3 mo: 2.0*, -2.3 (2.0) | Diff ∆*:  2 wk: -0.1  3 mo: 0.5  Stand. Diff Δ^*^:  2 wk: -0.1, p=0.80†  3-mo: 0.2, p=0.28† |
| Prvu Bettger, 2020[47] et al  AR  Some concerns | NRS (12 wk) | 5.2 (2.1)  2.7 (2.0), -2.5* | 5.7 (2.0)  3.0 (2.6), -2.7* | Diff ∆: 0.2* |
| Shim et al, 2023[48]  AR  Some concerns | NRS | 5.7 (2.1)  3 wk: 5.1 (2.0), -0.6*  12 wk: 2.8 (1.5), -2.9*  24 wk: 2.5 (1.6), -3.2* | 5.5 (2.2)  3 wk: 4.7 (2.0), -0.8*  12 wk: 3.3 (2.1), -2.2*  24 wk: 2.3 (2.1), -3.2* | Diff ∆*:  3 wk: 0.2  12 wk: -0.7  24 wk: 0 |
| **Adverse events** | | | | |
| Prvu Bettger et al, 2020[47]  AR  Some concerns | Number of falls, patient-reported (12 wk) | 19.4% | 14.6% | Diff ∆: 4.8%* |
| **Physical performance** | | | | |
| Eichler et al, 2019[31]  AR  High | 6-minute walk test (m) (3 mo) | 440.6 (78.2)  530.4 (79), 88.3 (57.7) | 433.3 (80.2)  513 (70.6), 79.6 (48.7) | Diff ∆: 8.7* |
|  | TUG (s) (3 mo) | 9.3 (1.8)  7.5 (1.2), –1.9 (1.5) | 9.0 (2.4)  7.5 (1.6), –1.5 (2.2) | Diff ∆: -0.4* |
|  | Stair Ascend Test (3 mo) | 8.7 (2.7)  6.2 (1.2), –2.5 (2.4) | 8.6 (4)  6.1 (1.5), –2.5 (3.0) | Diff ∆: 0* |
| Janhunen et al, 2023[35]  AR  Some concerns | TUG (s) (4 mo) | 9.4 (3.6)  2 mo: 8.2 (1.5), -1.2*  4 mo: 7.6 (1.5), -1.8* | 8.3 (1.7)  2 mo: 8.7 (1.6), 0.4*  4 mo: 7.7 (1.2), -0.6* | Diff ∆*:  2 mo: -1.6  4 mo: -1.2, p=0.04† |
|  | Active and passive knee flexion (4 mo) | 107 (18.0)  106*, -1.0 (15.2) | 107 (13.0)  100*, −7.0 (14.0) | Diff ∆*: 6.0, p=0.17† |
|  | Active and passive knee extension (4 mo) | 7.3 (7.7)  6.8*, -0.5 (7.0) | 6.6 (4.1)  6.7*, 0.1 (7.1) | Diff ∆*:  -0.6,  p=0.76† |
|  | Muscle force flexion (4 mo) | 0.6 (0.3)  0.7*, 0.1 (0.4) | 0.6 (0.2)  0.7*, 0.1 (0.1) | Diff ∆*: 0.0, p=0.88† |
|  | Muscle force extension (4 mo) | 1.2 (0.5)  1.1*, -0.1 (0.2) | 1.1 (0.4)  1.0*, −0.1 (0.3) | Diff ∆*: 0.0, p=0.85† |
|  | SPPB (4 mo) | 9.5 (1.5)  10.6*, 1.1 (1.5*) | 9.6 (1.5)  10.4*, 0.8 (1.6) | Diff ∆*: 0.3, p=0.51† |
| Jin et al, 2018[37]  VR  High | ROM (°)  (14 days) | 43.6 (3.4)  93.7 (6.6), 50.2* | 42.9 (3.6)  86.4 (5.0), 43.5* | Diff ∆*: 6.7, p=0.0†† |
| Mazurek et al, 2023[40]  VR  Some concerns | Rivermead Motor Assessment Gross-Function | 4.6 (2.4)  4 wk: 10.0 (1.0), 5.4* | 4.4 (2.6)  4 wk: 7.8 (2.5), 3.4* | Diff ∆*:  4 wk: 2.0 |
|  | Short Physical Performance Battery | 2.6 (2.3)  4 wk: 9.1 (2.4), 6.5* | 2.9 (2.4)  4 wk: 5.6 (5.6), 2.7* | Diff ∆*:  4 wk: 3.8 |
|  | Tinetti’s Short Scale | 3.3 (2.4)  4 wk: 9.6 (1.0), 6.3* | 3.6 (2.5)  4 wk: 6.5 (2.3), 2.9* | Diff ∆*:  4 wk: 3.4 |
| Nishitha et al, 2024[43]  VR  High | ROM Flexion | 12.5 (1.3)  4 wk: 62.3 (2.5), 49.8*  12 wk: 81.8 (3.5), 69.3* | 13.1 (0.8)  4 wk: 40.3 (4.1), 27.2*  12 wk: 73.1 (3.8), 60.0* | Diff ∆*:  4 wk: 22.6  12 wk: 9.3 |
| Piqueras et al, 2013[46]  AR  High | TUG (s) | 18.9 (7.34)  2 wk: NR, –3.36 (5.38)  3 mo: NR, –7.00 (6.31) | 22.8 (11.33)  2 wk: NR, 5.22 (6.25)  3 mo: NR, –10.86 (8.72) | Diff ∆*:  2 wk:-8.58  3 mo:3.86  Stand. Diff ∆*:  2 wk: -1.47  p=0.079†  3 mo: 0.51  p=0.020† |
|  | Active knee flexion | 96 (8.9)  2 wk: NR, 8.53 (6.56)  3 mo: NR, 18.16 (9.71) | 94.4 (6.9)  2 wk: NR, 7.71 (6.89)  3 mo: NR,15.63 (8.82) | Diff ∆*:  2 wk: 0.8  3 mo: 2.5  Stand. Diff ∆*:  2 wk: 0.12  p=0.298†  3 mo: 0.27  p=0.193† |
|  | Active knee extension | -2.8 (3.94)  2 wk: NR, 0.2 (2.8)  3 mo: NR, 1.3 (3.1) | -3.17 (3.5)  2 wk: NR, 0.9 (3.7)  3 mo: NR, 0.8 (3.3) | Diff ∆*:  2 wk: -0.7  3 mo: 0.5  Stand. Diff ∆*:  2 wk: -0.21  p=0.045†  3 mo: 0.15  p=0.478† |
|  | Quadriceps strength | 9.9 (3.5)  2 wk: NR, 2.98 (3.2)  3 mo: NR, 8.48 (4.96) | 9.7 (4.2)  2 wk: NR, 1.64 (2.60)  3 mo: NR, 5.89 (5.17) | Diff ∆*:  2 wk: 1.34  3 mo: 2.6  Stand. Diff ∆*:  2 wk: 0.45  p=0.011†  3 mo: 0.51  p=0.018† |
|  | Hamstring strength | 11 (3.95)  2 wk: NR, 1.17 (2.60)  3 mo: NR, 2.93 (3.33) | 10.4 (3.3)  2 wk: NR, 0.69 (2.22)  3 mo: NR, 2.26 (3.30) | Stand. Diff ∆*:  2 wk: 0.48  3 mo: 0.67  Stand. Diff ∆*:  2 wk: 0.2  p=0.278†  3 mo: 0.20  p=0.349† |
| Prvu Bettger et al, 2020[47]  AR  Some concerns | 10-m gait speed (s) (6 wk) | 1 (0.3)  1 (0.3), 0* | 1 (0.3)  1 (0.3), 0* | Diff ∆: 0* |
| Shim et al, 2023[48]  AR  Some concerns | 4-m gait speed | 0.4 (0.2)  3 wk: 0.5 (0.2), 0.1*  12 wk:0.8 (0.2), 0.4*  24 wk: 0.9 (0.2),0.5* | 0.5 (0.3)  3 wk: 0.6 (0.3), 0.1*  12 wk: 0.9 (0.1), 0.4*  24 wk: 1.0 (0.3), 0.4* | Diff ∆*:  3 wk: 0  12 wk: -0.1  24 wk: 0.1 |
|  | Berg balance scale | 29.8 (11.9)  3 wk: 34.7 (15.3), 4.9*  12 wk: 45.8 (11.9), 16.0*  24 wk: 49.3 (8.5), 19.5* | 29.7 (10.3)  3 wk: 37.6 (14.2), 7.9*  12 wk: 48.8 (11.3), 19.1*  24 wk: 47.9 (11.5), 18.2* | Diff ∆*:  3 wk: -3  12 wk: -3.1  24 wk: 1.3 |
|  | ROM | 79.0 (22.6)  3 wk: 96.6 (21.1), 17.6*  12 wk: 117.9 (11.8), 38.9*  24 wk: 120.6 (12.9), 41.6* | 82.0 (12.6)  3 wk: 106.0 (13.6), 24.0*  12 wk: 118.8 (12.8), 36.8*  24 wk: 122.3 (12.0), 40.3* | Diff ∆*:  3 wk: -6.4  12 wk: 2.1  24 wk: 1.3 |
|  | Quadriceps strength | NR  3 wk: 107.1 (35.2), NR  12 wk: 138.4 (29.7), NR  24 wk: 149.2 (37.6), NR | NR  3 wk: 98.1 (43.8), NR  12 wk: 131.1 (39.7), NR  24 wk: 146.5 (41.1), NR | Diff ∆:  3 wk: NR  12 wk: NR  24 wk: NR |
|  | Hamstring strength | NR  3 wk: 91.0 (34.1), NR  12 wk: 153.1 (34.2), NR  24 wk: 174.1 (42.0), NR | NR  3 wk: 103.2 (45.3), NR  12 wk: 146.9 (55.1), NR  24 wk: 157.7 (45.1), NR | Diff ∆:  3 wk: NR  12 wk: NR  24 wk: NR |
| **Quality of life** | | | | |
| Eichler et al, 2019[31]  AR  High | SF-36 physical component score (3 mo) | 33.8 (7.6)  44.6 (9.9), 10.7 (10.4) | 33.3 (7.9)  44.4 (8.3), 11.1 (7.2) | Diff ∆: -0.4* |
|  | SF-36 mental component score (3 mo) | 54.8 (10.6)  52.4 (10.6), –2.5 (12.4) | 53.9 (11.8)  54.1 (9.8), 0.1 (8.5) | Diff ∆: -2.6* |
| Prvu Bettger et al, 2020[47]  AR  Some concerns | PROMIS physical function (12 wk) | 12.5 (2.4)  15.3 (2.4), 2.8* | 12.3 (2.5)  14.8 (2.8), 2.5* | Diff ∆: 0.3* |
|  | PROMIS mental health (12 wk) | 15 (2.8)  16.6 (2.5), 1.6* | 14.9 (3.2)  16.1 (3.2), 1.2* | Diff ∆: 0.4* |
| Shim et al, 2023[48]  AR  Some concerns | EuroQoL-5D-5L | 0.4 (0.2)  3 wk: 0.6 (0.1), 0.2*  12 wk: 0.8 (0.1), 0.3*  24 wk: 0.8 (0.1), 0.4* | 0.5 (0.2)  3 wk: 0.6 (0.1), 0.1*  12 wk: 0.7 (0.1), 0.3*  24 wk: 0.8 (0.1), 0.3* | Diff ∆*:  3 wk: 0.02  12 wk: 0.1  24 wk: 0.1 |

AR=augmented reality; Diff Δ=difference in change scores; EuroQoL-5D=EuroQoL 5-dimension health survey; EuroQoL-5D-5L=EuroQoL 5-dimension 5-level health survey; IQR=interquartile range; KOOS=Knee Injury and Osteoarthritis Outcome Score; NC=not calculable; NR=not reported; NRS=numeric rating scale; OKS=Oxford Knee Score; PROMIS=Patient-Reported Outcomes Measurement Information System; ROM=range of motion; SD=standard deviation; SF-36=36-Item Short Form Health Survey; SPPB=Short Physical Performance Battery; TUG=Timed Up and Go test; VAS=Visual Analog Scale; VR=virtual reality; WOMAC=Western Ontario and McMaster Universities Osteoarthritis Index

References

31. Eichler S, Salzwedel A, Rabe S, et al. The effectiveness of telerehabilitation as a supplement to rehabilitation in patients after total knee or hip replacement: randomized controlled trial. JMIR Rehabil Assist Technol. Nov 7, 2019;6(2):e14236. [doi: 10.2196/14236] [Medline: 31697239]

33. Fuchs L, Kluska A, Novak D, Kosashvili Y. The influence of early virtual reality intervention on pain, anxiety, and function following primary total knee arthroplasty. Complement Ther Clin Pract. Nov 2022;49:101687. [doi: 10.1016/j.ctcp.2022.101687] [Medline: 36347152]

35. Janhunen M, Katajapuu N, Paloneva J, et al. Effects of a home-based, exergaming intervention on physical function and pain after total knee replacement in older adults: a randomised controlled trial. BMJ Open Sport Exerc Med. 2023;9(1):e001416. [doi: 10.1136/bmjsem-2022-001416] [Medline: 36896366]

[37. Jin C, Feng Y, Ni Y, Shan Z. Virtual reality intervention in postoperative rehabilitation after total knee arthroplasty: A prospective and randomized controlled clinical trial. Int J Clin Exp Med. 2018;11(6):6119-6124. URL: www.ijcem.com](www.ijcem.com)

40. Mazurek J, Cieślik B, Wrzeciono A, Gajda R, Szczepańska-Gieracha J. Immersive virtual reality therapy is supportive for orthopedic rehabilitation among the elderly: a randomized controlled trial. J Clin Med. Dec 14, 2023;12(24):7681. [doi: 10.3390/jcm12247681] [Medline: 38137750]

43. Nishitha K, Anitha A, Thaheera D. Effectiveness of virtual reality-based rehabilitation and high-intensity exercise program for total knee arthroplasty patients: a randomised controlled trial. J Clin Diagn Res. 2024;18(11). [doi: 10.7860/JCDR/2024/71122.20263]

46. Piqueras M, Marco E, Coll M, et al. Effectiveness of an interactive virtual telerehabilitation system in patients after total knee arthoplasty: a randomized controlled trial. J Rehabil Med. Apr 2013;45(4):392-396. [doi: 10.2340/16501977-1119] [Medline: 23474735]

47. Prvu Bettger J, Green CL, Holmes DN, et al. Effects of virtual exercise rehabilitation in-home therapy compared with traditional care after total knee arthroplasty: VERITAS, a randomized controlled trial. J Bone Joint Surg Am. Jan 15, 2020;102(2):101-109. [doi: 10.2106/JBJS.19.00695] [Medline: 31743238]

48. Shim GY, Kim EH, Lee SJ, et al. Postoperative rehabilitation using a digital healthcare system in patients with total knee arthroplasty: a randomized controlled trial. Arch Orthop Trauma Surg. Oct 2023;143(10):6361-6370. [doi: 10.1007/s00402-023-04894-y] [Medline: 37129691]
