# Supplementary material for: Extended Reality Interventions for Osteoarthritis of the Knee and Recovery After Total Knee Arthroplasty: Systematic Review and Meta-Analyses
Source: J Med Internet Res. 2026 Sep 8;28:e84753. doi: 10.2196/84753 (PMC13555783; doi:10.2196/84753)
Supplement: Supplementary file 11 [file jmir-v28-e84753-s013.docx]

# MCID Reference Table

| **Outcome Measure** | **Minimal Clinically Important Difference (MCID)** |
| --- | --- |
| WOMAC — Total | 12.5 (Salehi et al, 2023)[72] |
| WOMAC — Stiffness | 4.76 (Angst et al, 2018)[73] |
| WOMAC — Function | 11.25 (Angst et al, 2018) [73] |
| KOOS — ADL | 2.5 (Mills et al, 2016)[74] |
| KOOS — Quality of Life | 6.5 (Mills et al, 2016) [74] |
| Oxford Knee Score (OKS) | 6.1 (Martín-Fernández et al, 2017)[75] |
| Visual Analog Scale (VAS) | No established MCID |
| Numeric Rating Scale (NRS) | 1.0 (Salaffi et al, 2004)[76] |
| WHOQOL-BREF | No established MCID |
| CDC Health-Related Quality of Life | No established MCID |
| EuroQoL-5D | No established MCID |
| Tampa Scale of Kinesiophobia (TSK) | No established MCID |

References

72. Salehi R, Valizadeh L, Negahban H, Karimi M, Goharpey S, Shahali S. The Western Ontario and McMaster Universities Osteoarthritis, Lequesne Algofunctional index, Arthritis Impact Measurement Scale-short form, and Visual Analogue Scale in patients with knee osteoarthritis: responsiveness and minimal clinically important differences. Disability and rehabilitation. 2023;45(13):2185–91.

73. Angst F, Benz T, Lehmann S, Aeschlimann A, Angst J. Multidimensional minimal clinically important differences in knee osteoarthritis after comprehensive rehabilitation: a prospective evaluation from the Bad Zurzach Osteoarthritis Study. RMD Open. 2018;4(2).

74. Mills KA, Naylor JM, Eyles JP, Roos EM, Hunter DJ. Examining the minimal important difference of patient-reported outcome measures for individuals with knee osteoarthritis: a model using the knee injury and osteoarthritis outcome score. The Journal of rheumatology. 2016;43(2):395–404.

75. Martín-Fernández J, García-Maroto R, Sánchez-Jiménez FJ, Bau-González A, Valencia-García H, Gutiérrez-Teira B, et al. Validation of the Spanish version of the Oxford knee score and assessment of its utility to characterize quality of life of patients suffering from knee osteoarthritis: a multicentric study. Health and quality of life outcomes. 2017;15:1–11.

76. Salaffi F, Stancati A, Silvestri CA, Ciapetti A, Grassi W. Minimal clinically important changes in chronic musculoskeletal pain intensity measured on a numerical rating scale. European journal of pain. 2004;8(4):283–91.
