# Supplementary material for: Extended Reality Interventions for Osteoarthritis of the Knee and Recovery After Total Knee Arthroplasty: Systematic Review and Meta-Analyses
Source: J Med Internet Res. 2026 Sep 8;28:e84753. doi: 10.2196/84753 (PMC13555783; doi:10.2196/84753)
Supplement: Supplementary file 13 [file jmir-v28-e84753-s002.doc]

| **Population*** | Key Question 1 (KQ1): Adults (≥18 years) with chronic pain, defined as a condition that has chronic pain as a major symptom (*eg*, arthritis, fibromyalgia, phantom limb pain, diabetic neuropathy) AND/OR assessed pain of ≥90 days duration  Key Question 2 (KQ2): Adults (≥18 years) with any pain | Children (<18 years) |
| --- | --- | --- |
| **Intervention** | Extended reality (*ie*, augmented, mixed or fully immersive virtual reality) treatments with a primary aim of treating or preventing chronic pain | XR tools for providers; telehealth or virtual care that does not involve XR (only video conferencing, etc)  KQ1 only: XR interventions solely during surgery or other medical procedures |
| **Comparator** | Any | ― |
| **Outcomes** | Primary outcomes†  Pain-related functioning or interference (PRO)  Pain intensity or severity (PRO)  Pain global change (PRO)  Pain catastrophizing and kinesiophobia (PRO)  Quality of life (PRO)  Adverse events  Secondary outcomes  Opioid dose or use  Physical performance  Completion of medical treatments or adherence | ― |
| **Setting** | KQ1: Non-acute care settings (*eg*, outpatient clinic, home)  KQ2: Any | KQ1 only: Hospitalizations or emergency room visits |
| **Study**  **Design** | KQ1: RCTs with *N* ≥ 30 and follow-up ≥1 day post-treatment  KQ2: Observational cohorts and RCTs with *N* ≥ 30 and follow-up ≥ 90 days | Commentary, case series, case reports, reviews; *N* < 30  KQ1 only: observational studies |

*Notes*. *Population used in original report. The focus of this review is knee OA and TKA patients.

†To be eligible, study had to address at least 1 primary outcome.

*Abbreviations.* PRO=patient-reported outcomes; RCT=randomized controlled trial; XR=extended reality
