# Supplementary material for: Extended Reality Interventions for Osteoarthritis of the Knee and Recovery After Total Knee Arthroplasty: Systematic Review and Meta-Analyses
Source: J Med Internet Res. 2026 Sep 8;28:e84753. doi: 10.2196/84753 (PMC13555783; doi:10.2196/84753)
Supplement: Supplementary file 14 [file jmir-v28-e84753-s003.doc]

| **Search Date: 1946 to 05/01/23** |  | **Search Statement** | **Results** |
| --- | --- | --- | --- |
| **MEDLINE** | 1 | exp Chronic pain/ OR Pain.tw. | 753765 |
|  | 2 | Intractable pain/ or ((chronic or persistent or intractable or refractory or sustain*) adj3 pain).ti,ab,kf. | 100987 |
|  | 3 | exp Back pain/ or exp Neck pain/ or exp Patellofemoral Pain Syndrome/ or exp Mastodynia/ or (carpal tunnel or epicondylitis or Dupuytren's or tenosynovitis or trigger finger or "costovertebral angle pain" or mastalgia).ti,ab,kf. | 73784 |
|  | 4 | Mastodynia/ or (mastodynia? or breast pain? or mammalgia?).ti,ab,kf. | 1366 |
|  | 5 | exp Musculoskeletal pain/ or exp Arthralgia/ or exp Metatarsalgia/ or (polyarthriti* or monoarthriti* or osteoarthriti* or arthriti*).mp. | 361079 |
|  | 6 | (neuralgia or neuropathy or phantom limb or Complex Regional Pain Syndrom* or CRPS).mp. or (morton neuroma or piriformis muscle syndrome or sciatica).ti,ab,kf. | 127322 |
|  | 7 | (headache or migrain*).ti,ab,kf. | 110726 |
|  | 8 | exp Facial Pain/ or exp Glossalgia/ or ("burning mouth syndrome" or tic douloureaux).tw. | 11057 |
|  | 9 | exp Abdominal pain/ or exp Pelvic Pain/ or exp Flank Pain/ | 44672 |
|  | 10 | Cancer Pain/ or (cancer adj3 pain).ti,ab,kf. | 14262 |
|  | 11 | exp Nociceptive pain/ or exp Central Nervous System Sensitization/ or central sensiti#ation.tw. | 5757 |
|  | 12 | (somatic pain or tissue pain or nociceptive pain).ti,ab,kf. | 2072 |
|  | 13 | exp Myofascial Pain Syndromes/ | 6827 |
|  | 14 | (myofascial trigger point pain or  costen syndrome or temporomandibular joint dysfunction syndrome or tmj syndrome).ti,ab,kf. | 273 |
|  | 15 | (fibromyalgia or fibrositides or fibrositis or muscular rheumatism).mp. | 13994 |
|  | 16 | exp Muscle Spasticity/ or exp Paresis/ or (spasticity or hemiparesis).ti,ab,kf. | 35498 |
|  | 17 | Or/1-16 | 1304902 |
|  | 18 | ((Virtual or augmented or mixed) adj reality*).mp. or (VR or illusion* or ((Virtual or simulat* or immers* or 3D* or 3-D*) adj (environ* or techn*))).tw. | 48953 |
|  | 19 | exp Virtual reality exposure therapy/ or (Video gam* or exergam* or artificial intelligence or Wii or Nintendo or Kinect or Xbox or Playstation or Meta Quest or Oculus or HTC-Vive or HTC Vive or HP Reverb or Google Daydream or ((Head-mounted OR headmounted) adj display) or hippotherapy or (horse* adj2 simulat*) or cyberspace).mp. | 71578 |
|  | 20 | 18 or 19 | 116722 |
|  | 21 | 17 and 20 | 3612 |
|  | 22 | Limit to English | 3505 |
|  | 23 | Limit 22 to (books or chapter or editorial or erratum or letter or note) | 67 |
|  | 24 | 22 not 23 | 3458 |

| **Search Date: 1947 to 05/01/23** |  | **Search Statement** | **Results** |
| --- | --- | --- | --- |
| **Embase** | 1 | exp Chronic pain/ OR Pain.tw. | 1186047 |
|  | 2 | Intractable pain/ or ((chronic or persistent or intractable or refractory or sustain*) adj3 pain).ti,ab,kf. | 150686 |
|  | 3 | exp Back pain/ or exp Neck pain/ or exp Patellofemoral Pain Syndrome/ or exp Mastodynia/ or (carpal tunnel or epicondylitis or Dupuytren's or tenosynovitis or trigger finger or "costovertebral angle pain" or mastalgia).ti,ab,kf. | 197177 |
|  | 4 | Mastodynia/ or (mastodynia? or breast pain? or mammalgia?).ti,ab,kf. | 5687 |
|  | 5 | exp Musculoskeletal pain/ or exp Arthralgia/ or exp Metatarsalgia/ or (polyarthriti* or monoarthriti* or osteoarthriti* or arthriti*).mp. | 813297 |
|  | 6 | (neuralgia or neuropathy or phantom limb or Complex Regional Pain Syndrom* or CRPS).mp. or (morton neuroma or piriformis muscle syndrome or sciatica).ti,ab,kf. | 298182 |
|  | 7 | (headache or migrain*).ti,ab,kf. | 186866 |
|  | 8 | exp Facial Pain/ or exp Glossalgia/ or ("burning mouth syndrome" or tic douloureaux).tw. | 14953 |
|  | 9 | exp Abdominal pain/ or exp Pelvic Pain/ or exp Flank Pain/ | 247032 |
|  | 10 | Cancer Pain/ or (cancer adj3 pain).ti,ab,kf. | 34435 |
|  | 11 | exp Nociceptive pain/ or exp Central Nervous System Sensitization/ or central sensiti#ation.tw. | 7995 |
|  | 12 | (somatic pain or tissue pain or nociceptive pain).ti,ab,kf. | 3385 |
|  | 13 | exp Myofascial Pain Syndromes/ | 8889 |
|  | 14 | (myofascial trigger point pain or  costen syndrome or temporomandibular joint dysfunction syndrome or tmj syndrome).ti,ab,kf. | 347 |
|  | 15 | (fibromyalgia or fibrositides or fibrositis or muscular rheumatism).mp. | 27737 |
|  | 16 | exp Muscle Spasticity/ or exp Paresis/ or (spasticity or hemiparesis).ti,ab,kf. | 70069 |
|  | 17 | Or/1-16 | 2320301 |
|  | 18 | ((Virtual or augmented or mixed) adj reality*).mp. or (VR or illusion* or ((Virtual or simulat* or immers* or 3D* or 3-D*) adj (environ* or techn*))).tw. | 72207 |
|  | 19 | exp Virtual reality exposure therapy/ or (Video gam* or exergam* or artificial intelligence or Wii or Nintendo or Kinect or Xbox or Playstation or Meta Quest or Oculus or HTC-Vive or HTC Vive or HP Reverb or Google Daydream or ((Head-mounted OR headmounted) adj display) or hippotherapy or (horse* adj2 simulat*) or cyberspace).mp. | 86157 |
|  | 20 | 18 or 19 | 152523 |
|  | 21 | 17 and 20 | 6428 |
|  | 22 | Limit to English | 6209 |
|  | 23 | Limit 22 to (books or chapter or editorial or erratum or letter or note) | 259 |
|  | 24 | 22 not 23 | 5950 |

| **Search Date: 1987 to 05/30/23** |  | **Search Statement** | **Results** |
| --- | --- | --- | --- |
| **PsycInfo** | 1 | pain.tw. | 99143 |
|  | 2 | chronic pain/ or back pain/ or myofascial pain/ or (patellofemoral syndrome or carpal tunnel or epicondylitis or Dupuytren or tenosynovitis or trigger finger).tw | 19010 |
|  | 3 | (mastodynia or mammalgia or mastalgia).tw. | 22 |
|  | 4 | Exp Arthritis/ or (arthralgia or metatarsalgia or arthriti* or polyarthriti* or monoarthriti* or osteoarthriti*).tw. | 7939 |
|  | 5 | exp Headache/ or headache.tw. or migraine.tw. | 21361 |
|  | 6 | exp Neuralgia/ or exp Phantom Limbs/ or exp Neuropathy/ or (neuralgia or neuropathy or phantom limb or complex regional pain syndrom* or CRPS or morton neuroma or piriformis muscle syndrome or sciatica).tw | 11356 |
|  | 7 | (Glossalgia or "burning mouth syndrome" or tic douloureaux).tw. | 144 |
|  | 8 | “central nervous system sensitization”.tw. | 14 |
|  | 9 | exp Somatoform Pain Disorder/ or exp Myofascial Pain/ or tmj syndrome.tw. | 1157 |
|  | 10 | exp Fibromyalgia/ or (fibromyalgia or fibrositides or fibrositis or muscular rheumatism).tw. | 3799 |
|  | 11 | exp General Paresis/ or exp Hemiparesis/ or (muscle spasticity or hemiparesis).tw | 2033 |
|  | 12 | Or/ 1-11 | 127765 |
|  | 13 | virtual reality/ or virtual environment/ or virtual reality exposure therapy/ | 11428 |
|  | 14 | computer games/ or (Video gam* or exergam* or artificial intelligence or Wii or Nintendo or Kinect or Xbox or Playstation or Meta Quest or Oculus or HTC-Vive or HTC Vive or HP Reverb or Google Daydream or hippotherapy or ("Head-mounted display" or "headmounted display") or horse* simulat*).tw. or cyberspace.tw. | 21693 |
|  | 15 | 13 or 14 | 31648 |
|  | 16 | 12 and 15 | 608 |
|  | 17 | Limit to English | 585 |

| **Search Date: 1971 to 05/25/23** |  | **Search Statement** | **Results** |
| --- | --- | --- | --- |
| **CINAHL** | 1 | (MH "Chronic pain") OR (TI Pain OR AB Pain) |  |
|  | 2 | (MH "Back pain+") OR (MH "Neck pain") OR (MH "Musculoskeletal pain+") OR (MH “Muscle pain”) OR (MH Arthralgia+) OR (MH Arthritis+) OR (MH “Knee pain”) OR (MH "Patellofemoral Pain Syndrome") OR (MH “Elbow pain”) OR (MH “Heel pain”) OR (MH Metatarsalgia) OR (MH Tendinopathy+) |  |
|  | 3 | TI ("carpal tunnel" OR “tarsal tunnel” OR “Meralgia Paresthetica” OR epicondylitis OR Dupuytr* OR tenosynovitis OR "trigger finger" OR mastalgia# OR mastodynia# OR mammalgia# OR polyarthriti* OR monoarthriti* OR osteoarthriti* OR arthriti*) OR AB ("carpal tunnel" OR “tarsal tunnel” OR “Meralgia Paresthetica” OR epicondylitis OR Dupuytr* OR tenosynovitis OR "trigger finger" OR mastalgia# OR mastodynia# OR mammalgia# OR polyarthriti* OR monoarthriti* OR osteoarthriti* OR arthriti*) |  |
|  | 4 | (MH “Phantom pain”) OR (MH “Neuralgia+”) OR (MH “Referred pain”) OR (MH "Complex Regional Pain Syndromes+”) OR TI (neuralgia OR neuropathy OR "phantom limb" OR "Complex Regional Pain Syndrom*" OR CRPS OR "morton neuroma" OR "piriformis muscle syndrome" OR sciatica) OR AB (neuralgia OR neuropathy OR "phantom limb" OR "Complex Regional Pain Syndrom*" OR CRPS OR "morton neuroma" OR "piriformis muscle syndrome" OR sciatica) |  |
|  | 5 | (MH Headache+) OR TI( headache OR migrain*) OR AB (headache OR migrain*) |  |
|  | 6 | (MH "Facial Pain+") OR TI "burning mouth syndrome" OR AB "burning mouth syndrome" OR TI "tic douloureaux" OR AB "tic douloureaux" |  |
|  | 7 | (MH "Abdominal pain+") OR (MH "Pelvic Pain+") OR (MH "Cancer Pain") OR (MH "Nociceptive pain+") OR TI "central sensiti*" OR AB "central sensiti*" |  |
|  | 8 | (MH "Myofascial Pain Syndromes+") OR TI "myofascial trigger point" OR AB "myofascial trigger point" |  |
|  | 9 | MH “Temporomandibular Joint Syndrome” OR TI ("costen syndrome" OR “temporomandibular joint dysfunction” or TMJ) OR AB ("costen syndrome" OR “temporomandibular joint dysfunction” or TMJ) |  |
|  | 10 | MH fibromyalgia OR TI (fibromyalgia OR fibrositis OR "muscular rheumatism") OR AB (fibromyalgia OR fibrositis OR "muscular rheumatism") |  |
|  | 11 | (MH "Muscle Spasticity") OR TI (spasticity OR hemiparesis) or AB (spasticity OR hemiparesis) |  |
|  | 12 | S1 OR S2 OR S3 OR S4 OR S5 OR S6 OR S7 OR S8 OR S9 OR S10 OR S11 |  |
|  | 13 | (MH “Virtual reality+”) OR (MH “Augmented reality”) OR TI (((Virtual OR augmented OR mixed) W1 reality*) OR VR OR illusion* OR cyberspace) OR AB (((Virtual OR augmented OR mixed) W1 reality*) OR VR OR illusion* OR cyberspace) OR TI ((Virtual OR simulat* OR immers* OR 3D* OR 3-D*) W1 (environ* OR techn*)) OR AB ((Virtual OR simulat* OR immers* OR 3D* OR 3-D*) W1 (environ* OR techn*)) |  |
|  | 14 | (MH "Virtual reality exposure therapy") OR (MH “Video games+”) OR MH “Equine-assisted therapy” OR TI ("Video gam*" OR exergam* OR "artificial intelligence" OR Wii OR Nintendo OR Kinect OR Xbox OR Playstation OR "Meta Quest" OR Oculus OR HTC-Vive OR "HTC Vive" OR "HP Reverb" OR "Google Daydream" OR ((Head-mounted OR headmounted) W1 display) OR hippotherapy OR (horse* N2 simulat*)) OR AB ("Video gam*" OR exergam* OR "artificial intelligence" OR Wii OR Nintendo OR Kinect OR Xbox OR Playstation OR "Meta Quest" OR Oculus OR HTC-Vive OR "HTC Vive" OR "HP Reverb" OR "Google Daydream" OR ((Head-mounted OR headmounted) W1 display) OR hippotherapy OR (horse* N2 simulat*)) |  |
|  | 15 | S13 OR S14 |  |
|  | 16 | S12 AND S15 |  |
| ***Total*** | | | 20,727 |
| ***Total after deduplication*** | | | **10,932** |
